# Mechanistic basis for multidrug resistance and collateral drug sensitivity conferred to the malaria parasite by polymorphisms in PfMDR1 and PfCRT

**DOI:** 10.1371/journal.pbio.3001616

**Published:** 2022-05-04

**Authors:** Sarah Heckmatt Shafik, Sashika Natasha Richards, Ben Corry, Rowena Elizabeth Martin

**Affiliations:** Research School of Biology, The Australian National University, Canberra, Australian Capital Territory, Australia; The Pennsylvania State University, UNITED STATES

## Abstract

Polymorphisms in the *Plasmodium falciparum* multidrug resistance protein 1 (*pfmdr1*) gene and the *Plasmodium falciparum* chloroquine resistance transporter (*pfcrt*) gene alter the malaria parasite’s susceptibility to most of the current antimalarial drugs. However, the precise mechanisms by which PfMDR1 contributes to multidrug resistance have not yet been fully elucidated, nor is it understood why polymorphisms in *pfmdr1* and *pfcrt* that cause chloroquine resistance simultaneously increase the parasite’s susceptibility to lumefantrine and mefloquine—a phenomenon known as collateral drug sensitivity. Here, we present a robust expression system for PfMDR1 in *Xenopus* oocytes that enables direct and high-resolution biochemical characterizations of the protein. We show that wild-type PfMDR1 transports diverse pharmacons, including lumefantrine, mefloquine, dihydroartemisinin, piperaquine, amodiaquine, methylene blue, and chloroquine (but not the antiviral drug amantadine). Field-derived mutant isoforms of PfMDR1 differ from the wild-type protein, and each other, in their capacities to transport these drugs, indicating that PfMDR1-induced changes in the distribution of drugs between the parasite’s digestive vacuole (DV) and the cytosol are a key driver of both antimalarial resistance and the variability between multidrug resistance phenotypes. Of note, the PfMDR1 isoforms prevalent in chloroquine-resistant isolates exhibit reduced capacities for chloroquine, lumefantrine, and mefloquine transport. We observe the opposite relationship between chloroquine resistance-conferring mutations in PfCRT and drug transport activity. Using our established assays for characterizing PfCRT in the *Xenopus* oocyte system and in live parasite assays, we demonstrate that these PfCRT isoforms transport all 3 drugs, whereas wild-type PfCRT does not. We present a mechanistic model for collateral drug sensitivity in which mutant isoforms of PfMDR1 and PfCRT cause chloroquine, lumefantrine, and mefloquine to remain in the cytosol instead of sequestering within the DV. This change in drug distribution increases the access of lumefantrine and mefloquine to their primary targets (thought to be located outside of the DV), while simultaneously decreasing chloroquine’s access to its target within the DV. The mechanistic insights presented here provide a basis for developing approaches that extend the useful life span of antimalarials by exploiting the opposing selection forces they exert upon PfCRT and PfMDR1.

## Introduction

Elucidating the molecular mechanisms that underpin the malaria parasite’s acquisition of multidrug resistance is crucial to the ongoing efforts to control and eliminate malaria. Polymorphisms in 2 genes have been associated with many of the multidrug resistance phenotypes identified in field isolates of the parasite—the *Plasmodium falciparum* multidrug resistance protein 1 (*pfmdr1*) gene and the *Plasmodium falciparum* chloroquine resistance transporter (*pfcrt*) gene. Mutations in PfMDR1 and PfCRT, and/or variations in *pfmdr1* copy number, can alter the parasite’s susceptibility to diverse pharmacons, including most of the current antimalarial drugs and a number of the antimalarial candidates in the development pipeline [1]. Moreover, certain field isoforms of PfMDR1 and PfCRT increase the parasite’s susceptibility to lumefantrine, mefloquine, and dihydroartemisinin while simultaneously increasing resistance to chloroquine and amodiaquine [2–4]. However, the molecular basis for this pattern of collateral drug sensitivity—whereby resistance to one antimalarial drug causes sensitivity to another—remains unresolved.

PfMDR1 and PfCRT reside in the membrane of the parasite’s digestive vacuole (DV), the lysosomal-type compartment (pH 5.0 to 5.5) in which many antimalarials accumulate, act, and/or are activated. Both proteins are therefore ideally positioned to modulate the parasite’s susceptibility to many different drugs. In addition to their roles in mediating multidrug resistance, PfMDR1 and PfCRT are essential for parasite survival and are thus themselves potential drug targets [1,5]. Heterologous expression systems have been invaluable in characterizing the natural function of PfCRT as well as its roles in multidrug resistance [6–14]. PfCRT normally mediates the H^+^-dependent transport of hemoglobin-derived peptides from the DV into the parasite’s cytosol [10], but the mutations associated with drug resistance (which typically include K76T with either N75E or N326D [7,15–17]) alter its substrate range to include many other compounds, such as chloroquine, quinine, quinacrine, and methylene blue as well as the antiviral drug amantadine [6–9,18–22]. This enables the mutant isoforms of PfCRT to transport these drugs from the DV (where they would normally accumulate) back into the parasite’s cytosol.

Considerably less is understood about the function(s) of PfMDR1. The *pfmdr1* polymorphisms linked to multidrug resistance and collateral drug sensitivity include N86Y and N1042D. These mutations are associated with reductions in the parasite’s susceptibility to chloroquine (N86Y) [23–28] or quinine (N1042D) [29] as well as concomitant increases in the parasite’s sensitivity to lumefantrine and mefloquine [3,4,23,24,29–35]. However, it is not clear how these mutations, or any other polymorphisms in *pfmdr1*, alter the parasite’s susceptibility to drugs. PfMDR1 is a putative ATP-binding cassette (ABC) transporter (i.e., a pump that expends ATP to move solutes against their electrochemical gradients) and a homolog of the human P-glycoprotein (P-gp), which mediates multidrug resistance in cancer cells. The location of PfMDR1’s ATPase domains at the cytosolic face of the membrane indicates that it transports solutes into the DV [36]. Attempts to characterize PfMDR1 in heterologous expression systems have not provided robust results and/or direct measurements of its transport activity [1]. For example, an attempt to express PfMDR1 in *Xenopus* oocytes resulted in a poor transport signal-to-background ratio [37], and other efforts did not make direct measurements of drug transport and thus only made inferences about PfMDR1’s function from ATPase activity [38–41] (and one study was subsequently retracted [42]).

We sought to develop a reliable system for the heterologous expression of PfMDR1 that enables detailed examinations of its transport properties and thus a resolution of its roles in multidrug resistance and collateral drug sensitivity. Using *Xenopus* oocytes, we achieved a robust expression system that provides a very high transport signal-to-background ratio and which allows direct and high-resolution measurements of drug transport via PfMDR1. Our findings show that wild-type PfMDR1 transports structurally diverse antimalarial drugs, including lumefantrine, dihydroartemisinin, piperaquine, and methylene blue, as well as known substrates of human P-gp (e.g., vinblastine and rhodamine B [43–45]). We provide extensive validation of our system by showing, for example, (1) time and ATP dependence of PfMDR1-mediated transport; (2) inhibition of PfMDR1 by inhibitors of human P-gp; and (3) an absence of an interaction with amantadine (which also does not interact with human P-gp [46–48]).

In all bar one case, the introduction of field-derived mutations into PfMDR1 reduces the protein’s drug transport activity, and we also observe differences in transport capacities between the mutant isoforms as well as between drugs for a given isoform. The single exception is a mutant isoform that exhibits enhanced transport, relative to wild-type PfMDR1, of the antimalarials quinine and quinidine. By generating a homology model of PfMDR1, we highlight the interactions between quinine and the binding cavity of the transporter and propose that this creates an alternative binding site specific for quinine and quinidine.

Sequestration of a drug away from its primary target is a commonly observed resistance mechanism, and our finding of diverse transport capacities across multiple field isoforms of PfMDR1 suggests that the redistribution of drugs between the DV and the cytosol is a key mechanism by which polymorphisms in *pfmdr1* contribute to multidrug resistance phenotypes. We extend these mechanistic insights by delineating the roles of PfMDR1 and PfCRT in the collateral drug sensitivity patterns observed for lumefantrine, mefloquine, and chloroquine in the malaria parasite. We demonstrate that wild-type PfMDR1 has a greater capacity for the transport of these drugs than the mutant PfMDR1 isoforms. We observe the opposite trend for PfCRT: The mutations that confer chloroquine transport activity upon PfCRT also allow the protein to transport lumefantrine and mefloquine, whereas the wild-type protein lacks these activities. Hence, when present together, mutant isoforms of PfMDR1 and PfCRT change the distribution of these 3 drugs, such that they remain in the cytosol rather than accumulating within the DV. This evidently increases the access of lumefantrine and mefloquine to their primary targets (mefloquine is thought to act in the parasite cytosol [49,50] and the target(s) of lumefantrine are currently unknown) and thereby enhances their antimalarial activities. By contrast, the killing effect of chloroquine, which is primarily exerted against the detoxification of heme within the DV [51,52], is greatly diminished. Together, our datasets provide significant new mechanistic insights into how PfMDR1 and PfCRT contribute to multidrug resistance phenotypes.

## Results

### Expression and functional characterization of human P-gp and PfMDR1 in *Xenopus* oocytes

**Fig 1:**
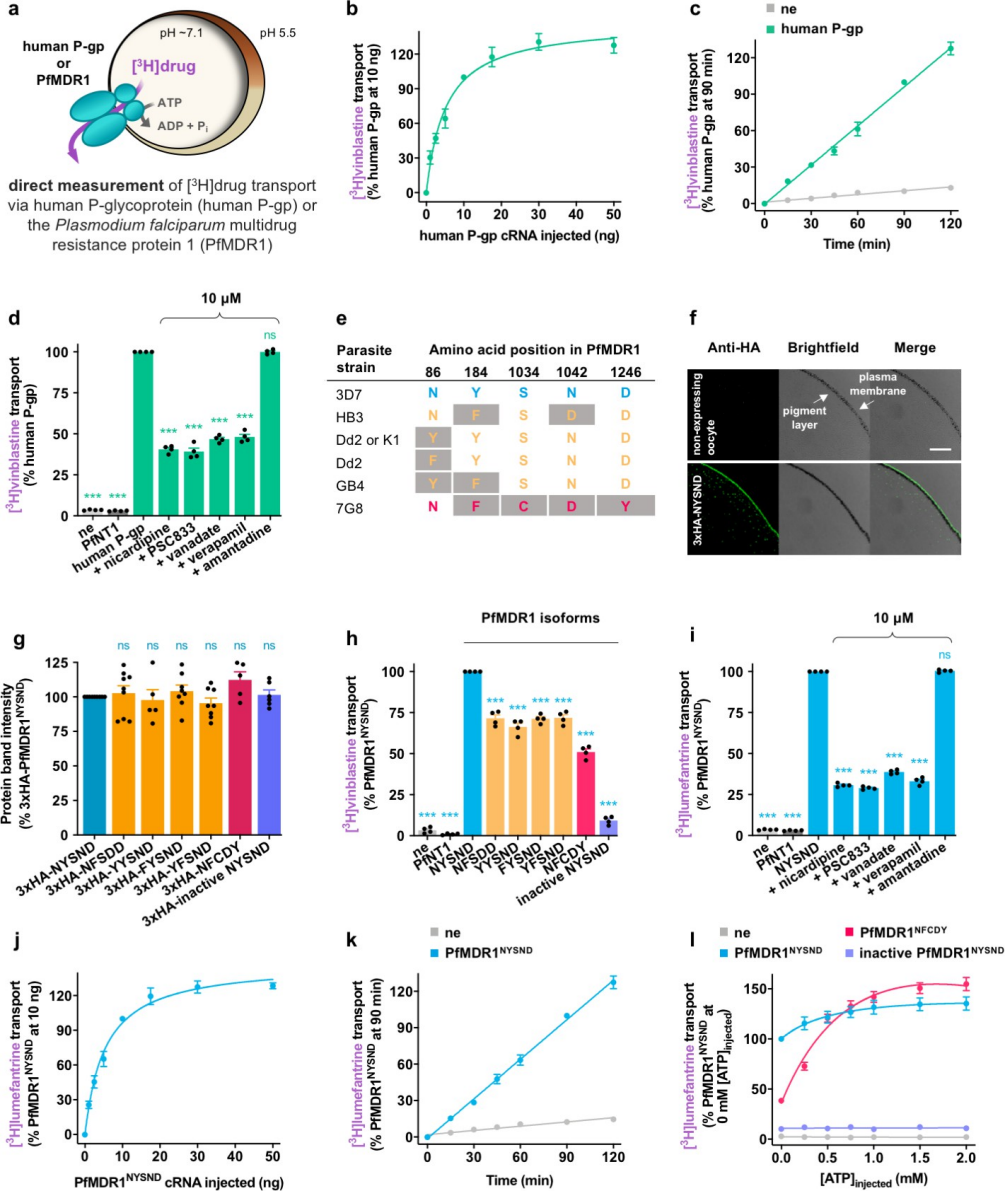
Heterologous expression of functional human P-gp and PfMDR1 in *Xenopus* oocytes. **(a)** Schematic showing the transport of a [^3^H]drug via human P-gp or PfMDR1 in the *Xenopus* oocyte system. **(b)** The relationship between the quantity of human P-gp cRNA injected into the oocyte and the level of human P-gp–mediated [^3^H]vinblastine transport measured. **(c)** The transport of [^3^H]vinblastine via human P-gp was approximately linear with time for at least 2 hours. **(d)** The human P-gp–mediated efflux of [^3^H]vinblastine from oocytes was reduced by known inhibitors of the transporter (nicardipine, PSC8333, vanadate, and verapamil) and was unaffected by amantadine (a drug that does not interact with human P-gp). **(e)** The field isoforms of PfMDR1 characterized in this study. Residues that differ from the wild-type amino acid sequence are shaded gray. **(f)** Immunofluorescence microscopy images confirmed that PfMDR1 localized to the oocyte plasma membrane. The expression of 3xHA-tagged PfMDR1^NYSND^ resulted in a fluorescent band external to the pigment layer, indicating that the protein was expressed at the oocyte surface. The band was not present in ne. The length of the scale bar is 50 μm. Images showing the presence of the other HA-tagged PfMDR1 isoforms at the oocyte surface are shown in S4 Fig. **(g)** Semiquantification of PfMDR1 protein levels in the membranes of oocytes expressing different 3xHA-PfMDR1 isoforms indicated that the 7 isoforms were expressed at similar levels. **(h)** The transport of [^3^H]vinblastine via PfMDR1. **(i)** The efflux of [^3^H]lumefantrine from oocytes expressing PfMDR1^NYSND^ was reduced by nicardipine, PSC8333, vanadate, and verapamil and was unaffected by amantadine. **(j)** The relationship between the quantity of PfMDR1^NYSND^ cRNA microinjected into the oocyte and the level of PfMDR1^NYSND^-mediated [^3^H]lumefantrine transport measured. **(k)** The transport of [^3^H]lumefantrine via PfMDR1^NYSND^ was approximately linear with time for at least 2 hours. **(l)** Microinjection of additional ATP into the oocyte greatly stimulated [^3^H]lumefantrine transport via PfMDR1^NFCDY^. The data are the mean of *n* = 4 to 9 independent experiments, each yielding similar results and overlaid as individual data points in panels **d**, **g**, **h**, and **i**, and the error is the SEM. Where not visible, the error bars fall within the symbols. The asterisks denote a significant difference from human P-gp (panel **d**), 3xHA-PfMDR1^NYSND^ (panel **g**), or PfMDR1^NYSND^ (panels **h** and **i**); ***P < 0.001, ns, not significant (1-way ANOVA). The data underlying this figure is supplied in S3 Data. ne, nonexpressing oocytes; PfMDR1, *Plasmodium falciparum* multidrug resistance protein 1; P-gp, P-glycoprotein.

Since human transporters are relatively straightforward to express in oocytes [53], human P-gp was first expressed in *Xenopus* oocytes to optimize the expression conditions and to develop a transport assay. The optimized methods were then applied to PfMDR1. Direct measurements of transport via human P-gp were undertaken by microinjecting a tritiated version of vinblastine ([^3^H]vinblastine; a known substrate of human P-gp) into the oocyte and measuring its efflux (Fig 1A). Given that the relationship between the quantity of cRNA microinjected into the oocyte and the level of [^3^H]vinblastine transport measured was approximately linear between 2.5 and 10 ng (Fig 1B), all subsequent experiments used oocytes microinjected with 10 ng of human P-gp cRNA. Next, we determined that the transport of [^3^H]vinblastine via human P-gp was approximately linear with time for at least 2 hours (Fig 1C). Hence, all of the subsequent transport measurements were made at 1.5 hours. To confirm that the [^3^H]vinblastine transport detected in these assays was mediated by human P-gp (and was not due to the nonspecific leakage of [^3^H]vinblastine from the oocyte), compounds known to interact with the transporter (nicardipine [46,54], PSC833 [55,56], vanadate [57,58], and verapamil [46,59]) were shown to inhibit [^3^H]vinblastine efflux (Fig 1D and S2 Fig). Furthermore, [^3^H]vinblastine transport was unaffected by amantadine—a drug that human P-gp does not interact with [46–48] (Fig 1D). Finally, we showed that other known substrates of human P-gp, such as rhodamine B, quinacrine, and methylene blue, are also transported by human P-gp in our oocyte system (S1 Data). The negative controls used in these experiments were nonexpressing oocytes (ne) and those expressing an unrelated transporter, the *Plasmodium falciparum* nucleoside transporter 1 (PfNT1; S1 Fig) [60,61]. The low level leakage of [^3^H]vinblastine from the negative control oocytes is most likely due to the simple diffusion of the neutral species of the drug (Fig 1C and 1D). Together, these datasets confirmed that we had achieved the functional expression of human P-gp in *Xenopus* oocytes, with the optimization process yielding relatively high rates of transport for vinblastine (304 ± 10 fmol/oocyte/h) and for other known substrates of human P-gp (S1 Data), as well as a high transport signal-to-background ratio (approximately 28-fold).

The optimized method and assays were then applied to wild-type PfMDR1 (PfMDR1^NYSND^), 5 field mutant isoforms of PfMDR1 (PfMDR1^NFSDD^, PfMDR1^YYSND^, PfMDR1^FYSND^, PfMDR1^YFSND^, and PfMDR1^NFCDY^), and a catalytically inactive version of PfMDR1^NYSND^ (Fig 1E, S1 Table, S3 Fig, and S1 File). The latter contains mutations in the protein’s nucleotide-binding domains (NBDs) that impede ATP hydrolysis. Immunofluorescence assays confirmed the presence of hemagglutinin (HA)-tagged versions of each isoform at the oocyte plasma membrane (Fig 1F and S4 Fig). Furthermore, PfMDR1 was shown to adopt the same orientation in the oocyte plasma membrane as it does in the membrane of the parasite’s DV [36], with its amino terminus and carboxyl terminus located in the cytosol (S4 Fig). Semiquantitative western blot analyses and densitometric evaluations of total protein confirmed that each of the PfMDR1 isoforms were expressed at comparable levels in the oocyte membrane (Fig 1G, S5 Fig, and S1 Text). Hence, any differences in drug transport activity between these PfMDR1 isoforms can be attributed to differences in their transport properties (and the mutations they carry), rather than to differences in expression levels.

All 6 field isoforms of PfMDR1 transported [^3^H]vinblastine, albeit to varying degrees (Fig 1H). The catalytically inactive version of PfMDR1^NYSND^ exhibited a very low level of [^3^H]vinblastine transport activity, consistent with the mutations in the NBDs hampering ATP hydrolysis. The ability of PfMDR1^NYSND^ to transport [^3^H]lumefantrine was then tested in the absence or presence of human P-gp inhibitors (Fig 1I). The PfMDR1^NYSND^-mediated transport of [^3^H]lumefantrine was inhibited by nicardipine, PSC833, vanadate, and verapamil, but was unaffected by amantadine (Fig 1I and S2 Fig). The relationship between the quantity of PfMDR1^NYSND^ cRNA microinjected into the oocyte and the level of [^3^H]lumefantrine transport achieved was very similar to that obtained for [^3^H]vinblastine transport via human P-gp (with 10 ng of PfMDR1^NYSND^ cRNA falling within the linear range) (Fig 1J) and the transport of [^3^H]lumefantrine via PfMDR1^NYSND^ was also approximately linear with time for at least 2 hours (Fig 1K). Furthermore, as was the case for human P-gp, a high rate of vinblastine transport (275 ± 3 fmol/oocyte/h) and a high transport signal-to-background ratio (approximately 29-fold) were obtained for PfMDR1^NYSND^. The microinjection of ATP into the oocyte greatly stimulated [^3^H]lumefantrine transport via PfMDR1^NFCDY^ but had a milder effect on PfMDR1^NYSND^ (Fig 1L). This is consistent with previous reports that D1246Y (located in NBD 2 of PfMDR1^NFCDY^) impedes ATP hydrolysis [39] and that PfMDR1^NFCDY^ has a lower basal ATPase activity than the wild-type protein [39,40].

### Field isoforms of PfMDR1 differ significantly in their capacities for antimalarial drug transport

**Fig 2:**
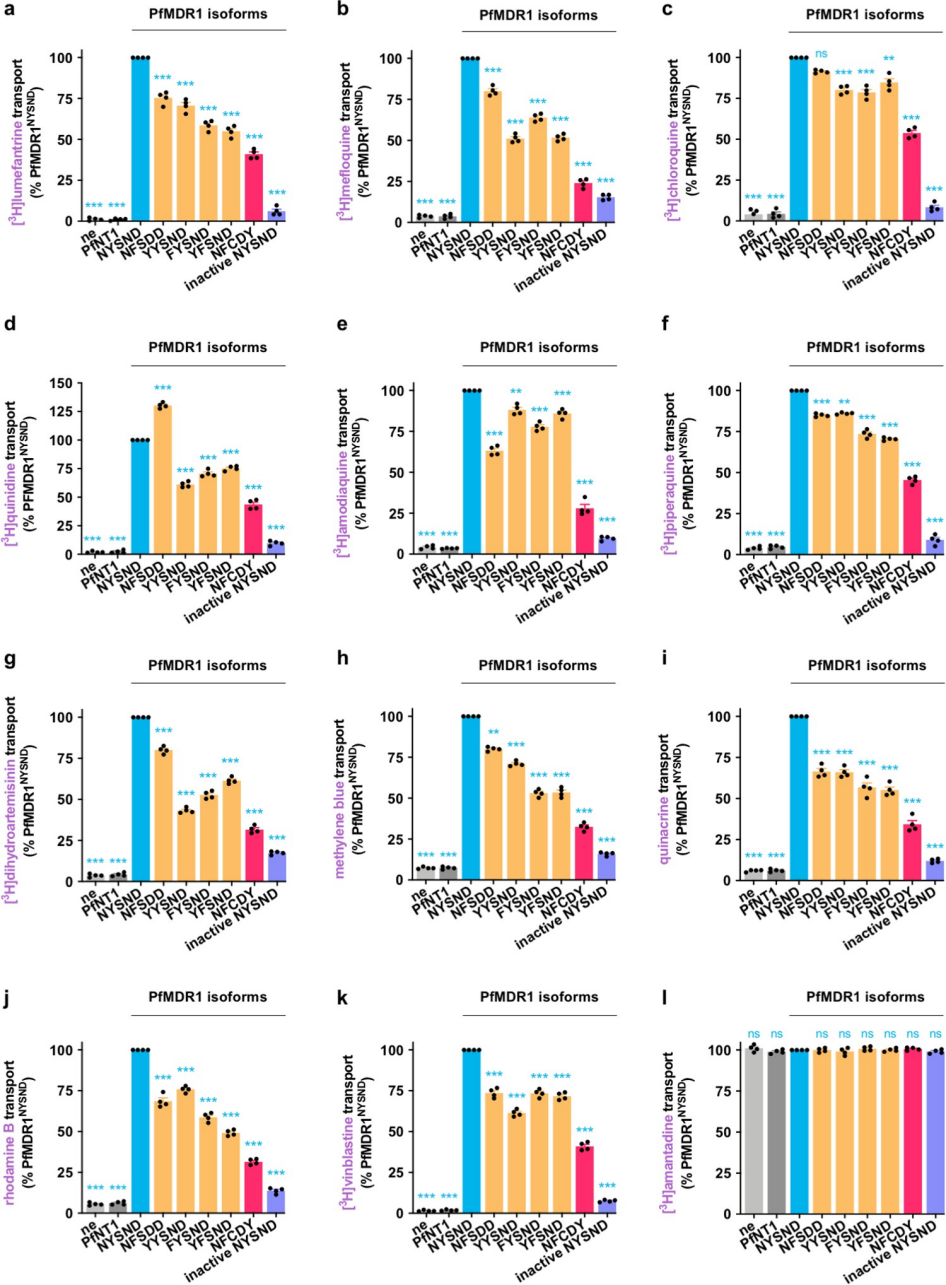
Field isoforms of PfMDR1 differ significantly in their capacities for antimalarial drug transport. **(a–i)** Field isoforms of PfMDR1 transported the antimalarial drugs [^3^H]lumefantrine (a), [^3^H]mefloquine (b), [^3^H]chloroquine (c), [^3^H]quinidine (d), [^3^H]amodiaquine (e), [^3^H]piperaquine (f), [^3^H]dihydroartemisinin (g), methylene blue (h), and quinacrine (i). **(j–l)** All of the field isoforms of PfMDR1 also transported the human P-gp substrates rhodamine B (j) and [^3^H]vinblastine (k), but none transported [^3^H]amantadine (l). The transport of methylene blue, quinacrine, and rhodamine B was detected using the intrinsic fluorescence of these compounds and a fluorescence-based transport assay (see S2 Text). The data are the mean of *n* = 4 independent experiments (each yielding similar results and overlaid as individual data points), and the error is the SEM. The asterisks denote a significant difference from PfMDR1^NYSND^; *P < 0.05, **P < 0.01, ***P < 0.001, ns, not significant (1-way ANOVA). The data underlying this figure is supplied in S3 Data. ne, nonexpressing oocytes; PfMDR1, *Plasmodium falciparum* multidrug resistance protein 1.

**Fig 3:**
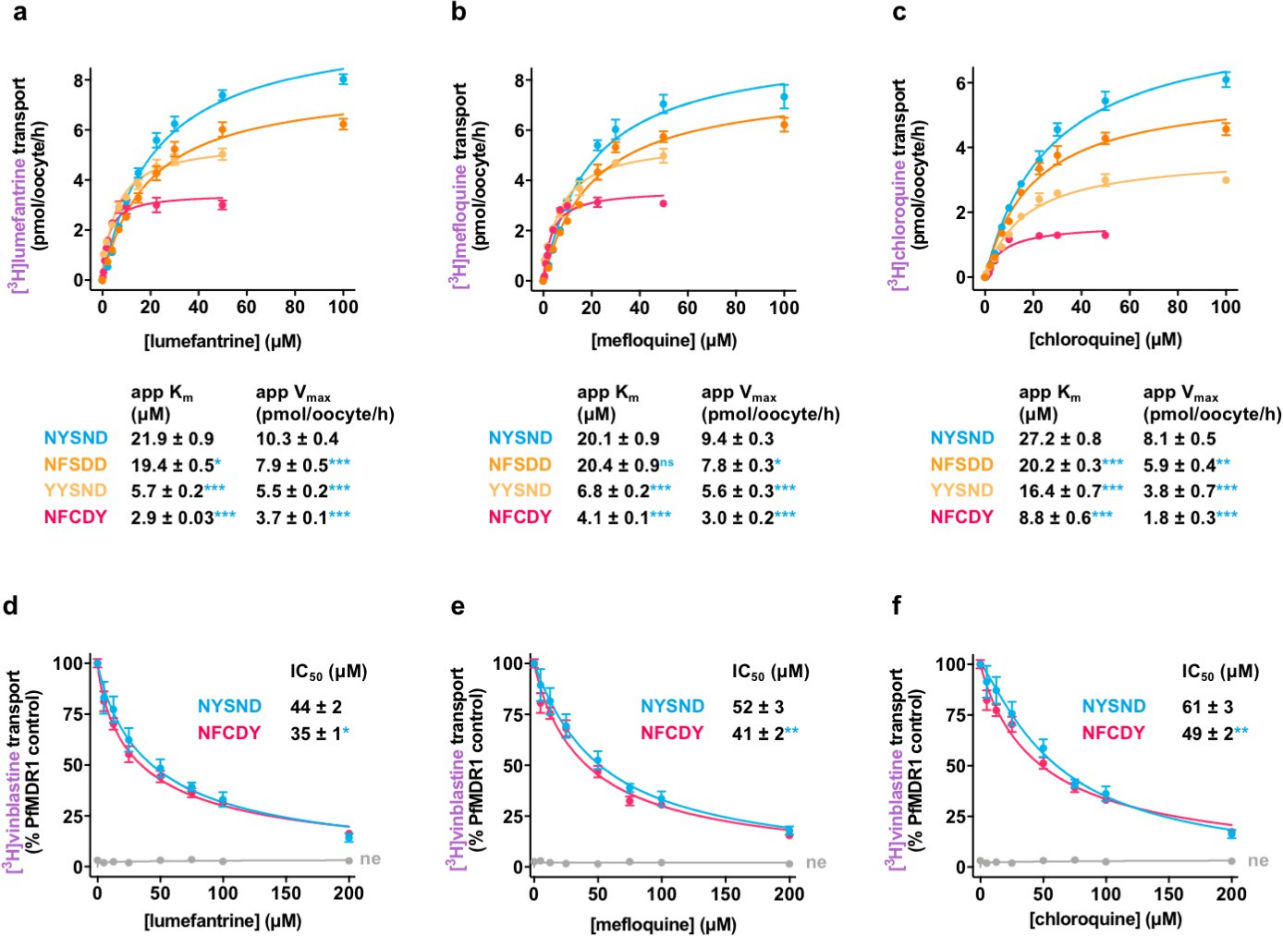
Characterization of lumefantrine, mefloquine, and chloroquine transport via PfMDR1. **(a–c)** There were significant differences in the apparent kinetic parameters of lumefantrine (a), mefloquine (b), and chloroquine (c) transport between field isoforms of PfMDR1. The concentration dependence of PfMDR1-mediated drug transport was calculated by subtracting the leakage from ne from that of oocytes expressing a PfMDR1 isoform at each drug concentration. **(d–f)** The transport of [^3^H]vinblastine via PfMDR1 was inhibited by lumefantrine (d), [^3^H]mefloquine (e), and [^3^H]chloroquine (f). The data are the mean of *n* = 4 independent experiments (each yielding similar results), and the error is the SEM. The asterisks denote a significant difference from PfMDR1^NYSND^; *P < 0.05, **P < 0.01, ***P < 0.001, ns nonsignificant (1-way ANOVA). The data underlying this figure is supplied in S3 Data. ne, nonexpressing oocytes; PfMDR1, *Plasmodium falciparum* multidrug resistance protein 1.

**Fig 4:**
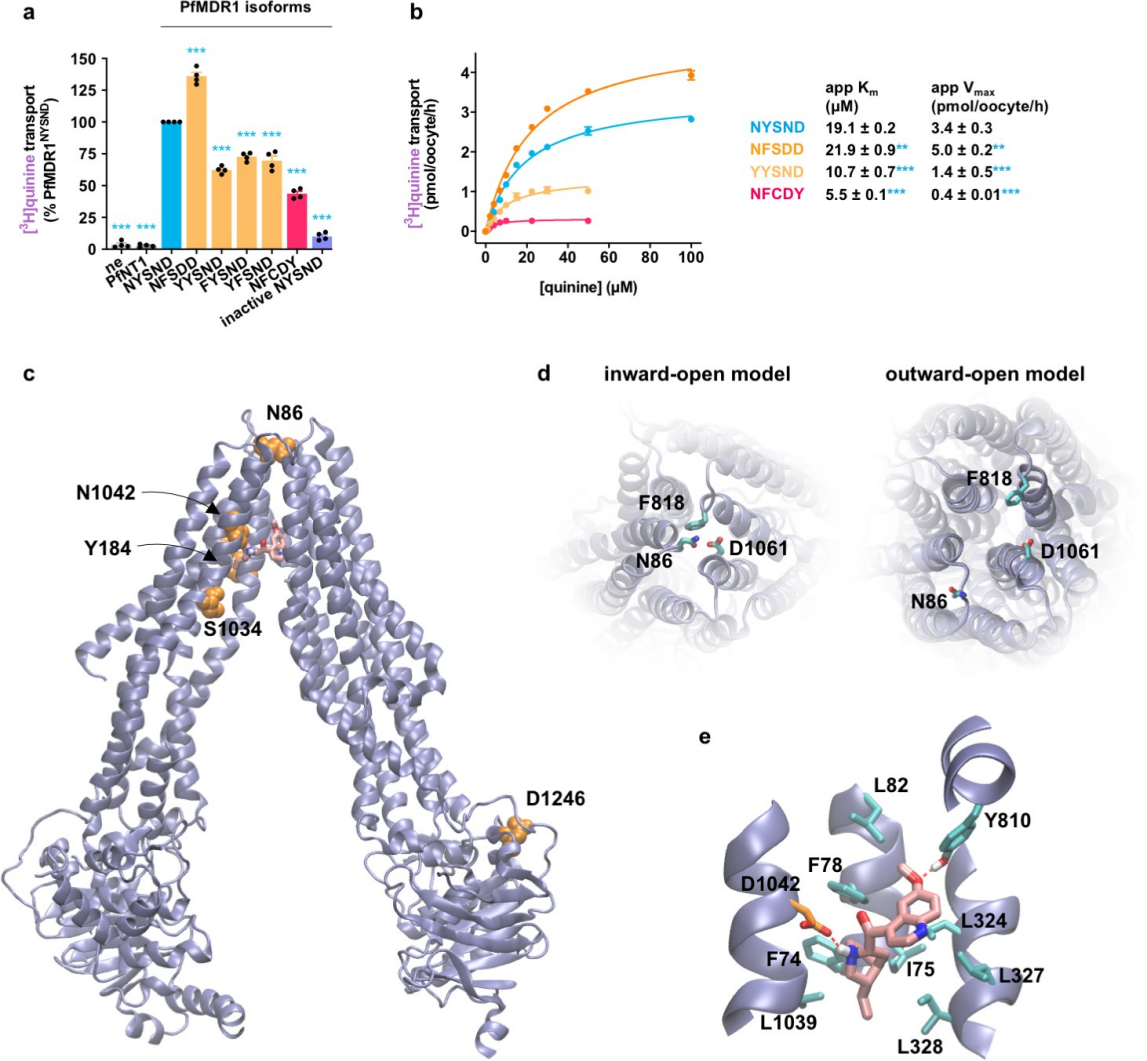
Quinine transport via PfMDR1. **(a)** Field isoforms of PfMDR1 transported [^3^H]quinine, with PfMDR1^NFSDD^ exhibiting the highest level of quinine transport activity. **(b)** There were significant differences in the apparent kinetic parameters for quinine transport via field isoforms of PfMDR1. The concentration dependence of PfMDR1-mediated quinine transport was calculated by subtracting the leakage from ne from that of oocytes expressing a PfMDR1 isoform at each drug concentration. The data are the mean of *n* = 4 independent experiments (each yielding similar results and overlaid as individual data points in panel **a**), and the error is the SEM. The asterisks denote a significant difference from PfMDR1^NYSND^; **P < 0.01 and ***P < 0.001 (1-way ANOVA). The data underlying panels **a** and **b** of this figure is supplied in S3 Data. **(c)** Inward-open homology model of PfMDR1 based on the *C*. *elegans* P-gp crystal structure (PDB 4F4C) showing the location of the mutations found in the 5 field PfMDR1 isoforms characterized in this study (orange). A putative binding pose of quinine in the central cavity is shown (pink). **(d)** A comparison of the inward-open model and outward-open models (based on the crystal structure of human P-gp; PDB 6C0V) as viewed from the side of the protein facing into the DV lumen. The N86Y mutation is part of a cluster of 3 residues forming the extracellular gate of the transporter (the participating residues are shown). These 3 residues are in close proximity in the inward-open state but move apart in the outward-open conformation. **(e)** Putative quinine binding site in PfMDR1^NFSDD^. Amino acids interacting with quinine are indicated (apart from L71, I1071, and F1072 that are removed to clearly view the binding pose). Atoms are shaded as follows: carbon in quinine, pink; carbon in the 1042D residue, orange; nitrogen, blue; oxygen, red; hydrogen, white. ne, nonexpressing oocytes; PfMDR1, *Plasmodium falciparum* multidrug resistance protein 1; P-gp, P-glycoprotein.

Our achievement of a robust expression system for PfMDR1 enabled direct characterizations of its function and of how this varies between different isoforms of the protein. We found that the 6 field isoforms of PfMDR1 exhibited varying capacities for the transport of a broad range of drugs and related compounds (Figs 2–4, S1 Data, and S2 Text). All of the antimalarial drugs tested—lumefantrine, mefloquine, chloroquine, quinine, quinidine, amodiaquine, piperaquine, dihydroartemisinin, methylene blue, and quinacrine—were shown to be substrates of the PfMDR1 isoforms included for study. The PfMDR1 isoforms also transported vinblastine and rhodamine B (known substrates of human P-gp), but none of the proteins transported amantadine (Fig 2 and S1 Data). The rates of antimalarial drug transport via PfMDR1^NYSND^ (in fmol/oocyte/h) were as follows: lumefantrine (731 ± 22), mefloquine (657 ± 23), amodiaquine (552 ± 11), chloroquine (531 ± 18), piperaquine (491 ± 17), quinidine (439 ± 11), quinine (432 ± 13), quinacrine (356 ± 11), methylene blue (333 ± 10), and dihydroartemisinin (293 ± 10). The low level of drug efflux observed from the negative control oocytes was most likely due to the simple diffusion of the neutral drug species.

In almost all cases, the introduction of mutations into PfMDR1^NYSND^ caused a significant reduction in drug transport activity. The most striking example of this observation was PfMDR1^NFCDY^, which had the lowest level of transport activity for all of the compounds tested (Figs 2–4 and S1 Data). The rates of antimalarial drug transport via PfMDR1^NFCDY^ (in fmol/oocyte/h) and the corresponding fold change relative to drug transport via PfMDR1^NYSND^ were as follows: lumefantrine (299 ± 23, approximately 0.4-fold), chloroquine (286 ± 11, approximately 0.5-fold), piperaquine (223 ± 9, approximately 0.5-fold), quinidine (191 ± 8, approximately 0.4-fold), quinine (188 ± 10, approximately 0.4-fold), mefloquine (175 ± 7, approximately 0.3-fold), amodiaquine (155 ± 9, approximately 0.3-fold), quinacrine (121 ± 4, approximately 0.3-fold), methylene blue (108 ± 3, approximately 0.3-fold), and dihydroartemisinin (92 ± 3, approximately 0.3-fold).

No 2 drugs produced the same profile of transport activities across the 6 field isoforms of PfMDR1. There were, however, strong similarities between some of the datasets (see S3 Text for an extended analysis). For example, commonalities were evident within the following groups (1) lumefantrine and mefloquine; (2) chloroquine and piperaquine; (3) mefloquine, quinine, quinidine, and dihydroartemisinin; and (4) lumefantrine, methylene blue, and quinacrine (Figs 2 and 4, and S3 Text).

### Characterization of lumefantrine, mefloquine, and chloroquine transport via PfMDR1

Having identified that the 6 field isoforms of PfMDR1 exhibit different capacities for the transport of lumefantrine, mefloquine, and chloroquine (Fig 2A–2C), we characterized the interactions of these 3 drugs with the transporter in more detail. A kinetic analysis of the PfMDR1-mediated transport of lumefantrine, mefloquine, and chloroquine revealed that the apparent Michaelis constant (app K_m_) and the apparent maximal velocity (app V_max_) values decreased in the order PfMDR1^NYSND^ ≥ PfMDR1^NFSDD^ > PfMDR1^YYSND^ > PfMDR1^NFCDY^ (Fig 3A–3C). Hence, PfMDR1^NFCDY^ has both the lowest app K_m_ and the lowest app V_max_ for all 3 antimalarials. PfMDR1^NFCDY^ is the only isoform included in this study that contains D1246Y. This mutation is located upstream of the Q-loop within NBD 2, which is thought to be involved in coordinating Mg^2+^ for ATP hydrolysis and/or in facilitating conformational changes of the NBDs in human P-gp [62–65]. Thus, it is possible that D1246Y disrupts ATP hydrolysis and/or the coupling of ATP hydrolysis to drug transport in PfMDR1, and that this contributes to the lower drug transport activity exhibited by PfMDR1^NFCDY^.

A subset of experiments measuring the abilities of lumefantrine, mefloquine, and chloroquine to inhibit [^3^H]vinblastine transport via PfMDR1^NYSND^ and PfMDR1^NFCDY^ was performed to ascertain whether these drugs are potent inhibitors of the transporter. None of the antimalarials were potent inhibitors of transport via either isoform of the protein, with all 3 drugs producing IC_50_s that are 20- to 11-fold higher than those obtained with nicardipine (Fig 3D–3F and S2 Fig). Moreover, in all cases, the antimalarials were slightly more effective in inhibiting transport via the mutant field isoform. This is not the trend that would be expected if these drugs exerted an antimalarial effect against wild-type PfMDR1 (e.g., by blocking the transport of its (currently unknown) natural substrates) that is alleviated by the introduction of mutations into the transporter.

### Quinine transport via PfMDR1

Although the addition of mutations to PfMDR1^NYSND^ reduced drug transport activity in most cases, there were 2 exceptions to this trend. The introduction of the Y184F and N1042D mutations—yielding PfMDR1^NFSDD^—increased the protein’s capacity for the transport of both quinine and its stereoisomer quinidine (Figs 2D and 4A). A kinetic analysis of quinine transport via PfMDR1 revealed several valuable insights (Fig 4B). First, the range of app K_m_ values obtained for quinine transport via the different PfMDR1 isoforms (5.5 ± 0.1 μM to 21.9 ± 0.9 μM) was similar to those observed for the transport of lumefantrine, mefloquine, and chloroquine (e.g., the lumefantrine app K_m_ values ranged from 2.9 ± 0.03 μM to 21.9 ± 0.9 μM). By contrast, the app V_max_ values for quinine transport (0.4 ± 0.01 to 5.0 ± 0.2 pmol/oocyte/h) were lower than those obtained for the other 3 drugs (e.g., the lumefantrine app V_max_ values were 3.7 ± 0.1 to 10.3 ± 0.4 pmol/oocyte/h). In general terms, these observations indicate that quinine occupies the transporter’s binding cavity for a much longer period than lumefantrine, mefloquine, or chloroquine. It is possible that in the parasite, this relatively low rate of quinine translocation impedes the transport of the natural substrates of PfMDR1.

The second key observation from the kinetic analysis of quinine transport was the finding that PfMDR1^NFSDD^ has a higher app V_max_ (5.0 ± 0.2 pmol/oocyte/h versus 3.4 ± 0.3 pmol/oocyte/h) as well as a slightly higher app K_m_ (21.9 ± 0.9 μM versus 19.1 ± 0.2 μM) than the wild-type protein (Fig 4A and 4B). We sought a structural explanation for this unusual result by generating a homology model of PfMDR1 to determine how quinine interacts with the transporter’s binding cavity.

Two homology models of PfMDR1 have previously been generated using either the bacterial MsbA protein or mouse P-gp as the template sequences [66,67]. However, the low sequence identity between PfMDR1 and these sequences (26% to 29%) reduces the versatility of these homology models. We identified the recently elucidated structures of *Caenorhabditis elegans* P-gp [68] and human P-gp [69], which have slightly higher sequence identity (33%), as better templates for inward-open and outward-open homology models of PfMDR1 (S1 File), although structural predictions should still be made with caution at this level of sequence identity. The locations of the 5 field mutations are depicted in the inward-open model of PfMDR1 (Fig 4C). Consistent with previous reports, both S1034 and N1042 line the central cavity (where they could interact with substrates), Y184 faces into the membrane, and D1246 is located in NBD 2 of the protein [66,67]. However, contrary to previous models that suggested that N86 forms part of the substrate-binding cavity [66,67], our inward-open model proposes that N86 is located on the side of the protein that faces into the DV lumen, forming part of the gate and obscuring the central cavity of the protein from this compartment (Fig 4D). A comparison with the outward-open model shows that the residues in this gate (N86, F818, and D1061) move far apart to expose the substrate-binding cavity to the DV lumen (Fig 4D). Thus, it is plausible that mutation of N86 could alter these interactions and consequently stabilize the inward-open conformation.

Since the Y184F mutation in PfMDR1^NFSDD^ protrudes into the membrane (rather than into the binding cavity), it is unlikely to be involved in coordinating quinine. Thus, it is more likely that the N1042D mutation is the cause of the enhanced quinine transport activity exhibited by PfMDR1^NFSDD^. To investigate this hypothesis, we used molecular dynamics simulations of PfMDR1 containing N1042D to examine the likely binding poses of quinine in the binding cavity. Seven simulations were performed, each with a different random starting position and orientation of quinine inside the substrate-binding cavity. In 4 of the 7 simulations, quinine quickly moved to interact with 1042D (within 20 ns; S6 Fig). This interaction with the negatively charged carboxylate oxygen of the aspartate residue occurred through the protonated nitrogen on the cyclic amine ring of quinine. Indeed, quinine adopted a very stable position in a predominantly hydrophobic pocket in which 3 of the 4 polar atoms on the molecule found hydrogen bonding partners on the protein (stabilized by the nitrogen on the cyclic amine ring, as well as the central hydroxyl group on the quinine molecule forming hydrogen bonds with 1042D, in addition to the ether oxygen of quinine forming a hydrogen bond with Y810; Fig 4E). Thus, the stability of the putative quinine binding site (as determined in our simulations) could plausibly represent an alternative binding site for the drug in PfMDR1^NFSDD^ that is not present in the other isoforms. This could alter the rate of quinine transport by influencing the rate of the protein conformational change or, perhaps less likely, ligand unbinding.

### Correlations between rates of drug transport via PfMDR1 in *Xenopus* oocytes and in vitro drug resistance indices

**Fig 5:**
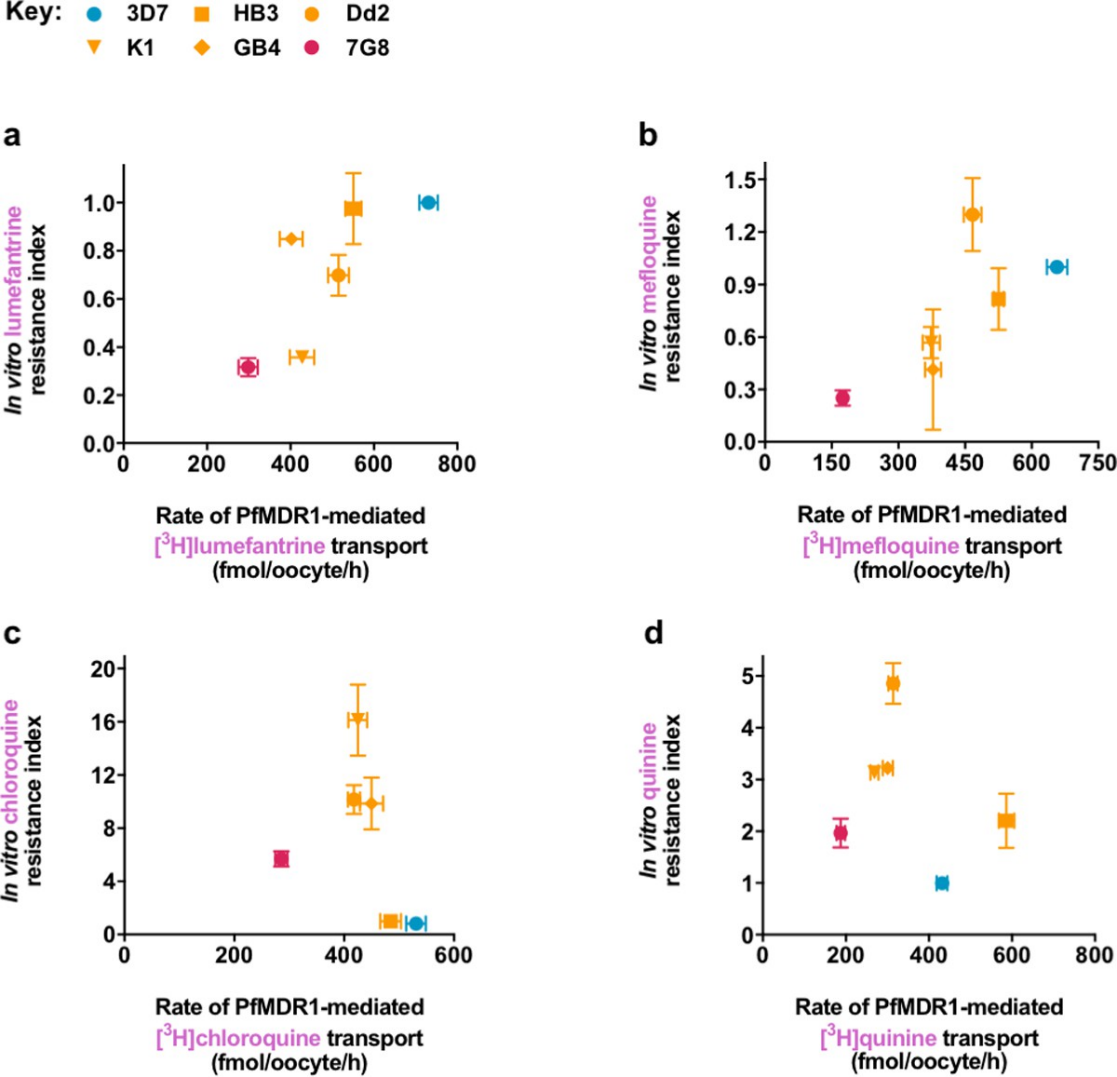
Correlations between rates of drug transport via PfMDR1 in *Xenopus* oocytes and in vitro parasite drug resistance indices. The rates of PfMDR1-mediated transport of lumefantrine, mefloquine, chloroquine, and quinine (Figs 2 and 4, and S1 Data) were plotted against the in vitro resistance index for the relevant drug and parasite strain (S3 Table). Where not shown, error bars fall within the symbols. **(a)** A positive correlation was observed between the rate of lumefantrine transport via PfMDR1 and the in vitro lumefantrine response (Pearson correlation coefficient = 0.76, r^2^ = 0.58, *P* = 0.078). **(b)** A positive correlation was observed between the rate of mefloquine transport via PfMDR1 and the in vitro mefloquine response (Pearson correlation coefficient = 0.75, r^2^ = 0.56, *P* = 0.008). The data point for Dd2 is an outlier and its removal significantly improved the correlation (Pearson correlation coefficient = 0.97, r^2^ = 0.94, *P* = 0.006). Dd2 parasites typically harbor 2 to 4 copies of PfMDR1 and have been reported to express greater levels of PfMDR1 than the other parasite strains included in the analysis [85,87]. Given that *pfmdr1* amplification has been associated with mefloquine resistance [30–32,88,89], it is possible that the overexpression of PfMDR1 in Dd2 parasites imparts a higher level of resistance to this strain (see S3 Text for an extended analysis). **(c)** An inverse correlation was observed between the rate of chloroquine transport via PfMDR1 and the in vitro chloroquine response. The removal of the 7G8 data point (see S3 Text for an extended analysis) resulted in a stronger correlation between the rate of chloroquine transport via PfMDR1 and the parasite’s chloroquine response in vitro (Pearson correlation coefficient = −0.86, r^2^ = 0.74, *P* = 0.054). **(d)** There was no correlation between the rate of quinine transport via PfMDR1 and the in vitro quinine response (see S4 Text for an extended analysis). The data for the in vitro resistance indices are the mean of *n* = 2 to 18 published studies, and the data for the PfMDR1-mediated drug transport rates are the mean of *n* = 4 independent experiments. The error is the SEM except for the in vitro resistance indices that were calculated from 2 studies, in which case the error is the range/2. The data underlying this figure is supplied in S3 Data. PfMDR1, *Plasmodium falciparum* multidrug resistance protein 1.

We used the PfMDR1-mediated rates of lumefantrine, mefloquine, chloroquine, and quinine transport obtained in the *Xenopus* oocyte system (S1 Data) to determine relationships between the drug transport capacities of the different PfMDR1 field isoforms and the in vitro drug response of parasite strains that express these proteins. In vitro drug resistance indices were calculated using IC_50_ values collated from the literature [16,23,24,26,70–86] (S3 Table) and then plotted against the PfMDR1-mediated transport rate for the relevant drug and parasite strain. We found strong positive correlations for lumefantrine (Pearson correlation coefficient = 0.76; Fig 5A and S3 Text) and mefloquine (Pearson correlation coefficient = 0.75; Fig 5B and S3 Text), and a strong negative correlation for chloroquine (Pearson correlation coefficient = −0.86; Fig 5C and S3 Text). No correlation was found between the rate of quinine transport via PfMDR1 and the in vitro quinine response (Fig 5D and S4 Text).

### Transport of lumefantrine and mefloquine via PfCRT in *Xenopus* oocytes and in situ

**Fig 6:**
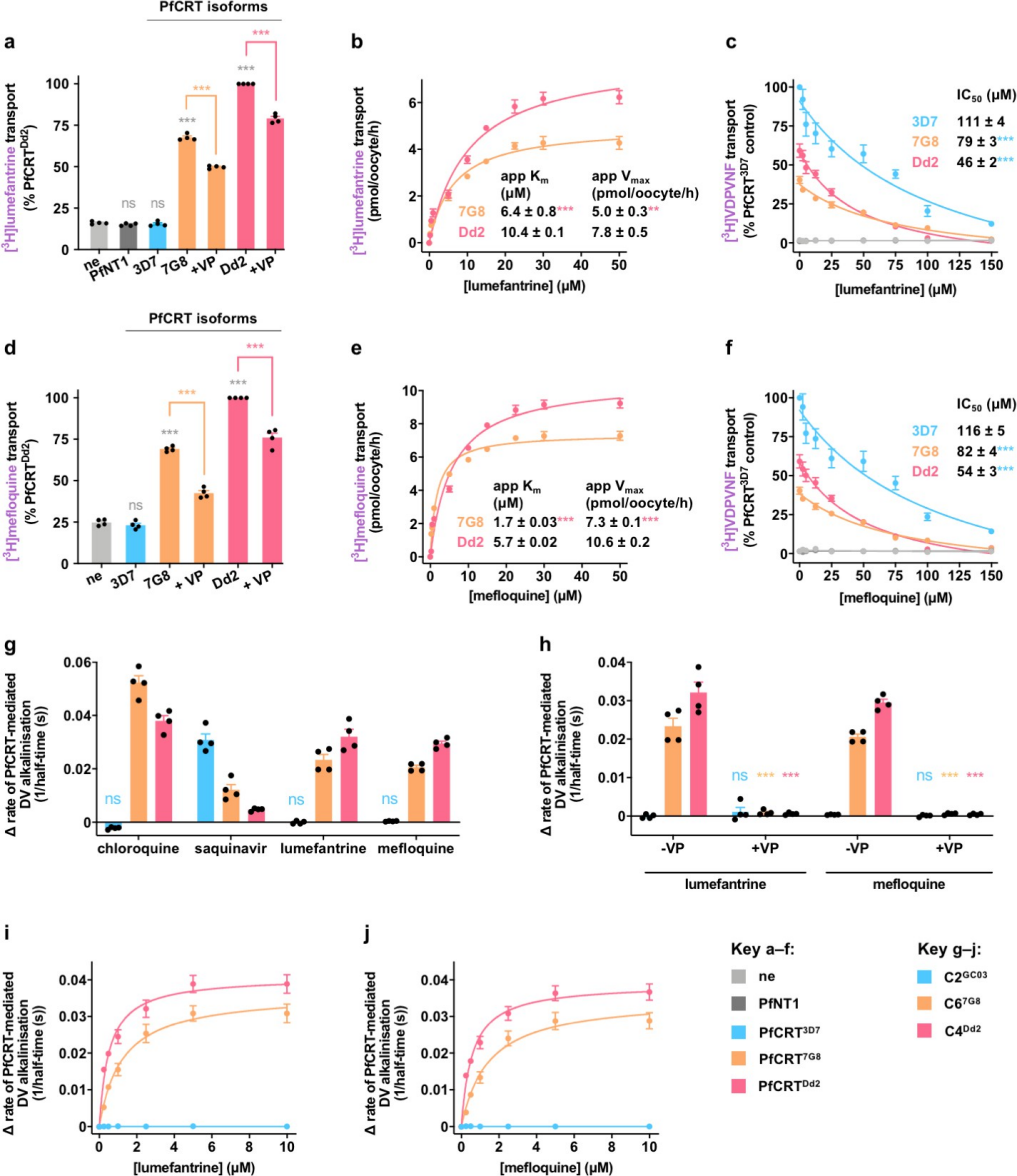
Transport of lumefantrine and mefloquine via PfCRT in *Xenopus* oocytes and in situ. **(a, d)** The transport of [^3^H]lumefantrine (a) and [^3^H]mefloquine (d) via PfCRT^Dd2^ and PfCRT^7G8^ was reduced by verapamil (VP; 100 μM). The asterisks denote a significant difference from the ne (gray asterisks), the PfCRT^7G8^ control (orange asterisks), or the PfCRT^Dd2^ control (red asterisks). **(b, e)** The transport of lumefantrine (b) and mefloquine (e) via PfCRT^Dd2^ and PfCRT^7G8^ was saturable. The asterisks denote a significant difference from PfCRT^Dd2^ (red asterisks). **(c, f)** The effects of unlabeled lumefantrine (c) and unlabeled mefloquine (f) on [^3^H]VDPVNF transport via PfCRT^3D7^, PfCRT^Dd2^, and PfCRT^7G8^. The asterisks denote a significant difference from PfCRT^3D7^ (blue asterisks). **(g)** Lumefantrine and mefloquine (2.5 μM) increased the rate of DV alkalinization in the chloroquine-resistant C6^7G8^ and C4^Dd2^ parasite lines but not in the chloroquine-sensitive C2^GC03^ line. Unless labeled ns, *P* < 0.05 relative to the absence of a test solute. **(h)** The increase in the rate of DV alkalinization caused by lumefantrine and mefloquine was inhibited by VP (50 μM). The asterisks denote a significant difference from the relevant C2^GC03^ (blue asterisks), C6^7G8^ (orange asterisks), or C4^Dd2^ (red asterisks) treatments in the absence of verapamil. **(i, j)** Lumefantrine (i) and mefloquine (j) increased the rate of DV alkalinization in a concentration-dependent manner. The data are the mean of *n* = 4 independent experiments, each yielding similar results and overlaid as individual data points in panels **a**, **d**, **g**, and **h**, and the error is the SEM. Where not visible, the error bars fall within the symbols. **P < 0.01, ***P < 0.001, ns, not significant (1-way ANOVA). The data underlying this figure is supplied in S3 Data. ne, nonexpressing oocytes; PfCRT, *Plasmodium falciparum* chloroquine resistance transporter.

We sought to elucidate the role of PfCRT in modulating the parasite’s response to lumefantrine and mefloquine by measuring its ability to transport these drugs in *Xenopus* oocytes (see S2 Table and S3 Fig for the positions of the mutations in the PfCRT isoforms studied here). Wild-type PfCRT (PfCRT^3D7^) did not transport lumefantrine or mefloquine, whereas 2 chloroquine resistance-conferring isoforms of PfCRT (PfCRT^7G8^ and PfCRT^Dd2^) exhibited significant transport of both drugs (Fig 6A and 6D and S7 Fig). The transport of lumefantrine and mefloquine via PfCRT^7G8^ and PfCRT^Dd2^ was inhibited by compounds known to interact with the transporter, including verapamil [6,18], chlorpheniramine [90], and saquinavir [10,91], but was unaffected by histidine and the dipeptide LH (neither of which interact with PfCRT [10,91]) (Fig 6A and 6D, and S7 Fig). Lumefantrine and mefloquine transport via PfCRT^7G8^ and PfCRT^Dd2^ was saturable, with PfCRT^Dd2^ having a slightly lower affinity and a higher capacity for drug transport relative to PfCRT^7G8^ (Fig 6B and 6E). When compared with chloroquine transport via PfCRT^Dd2^ (K_m_ = 232 ± 11 μM; V_max_ = 61 ± 6 pmol/oocyte/h [7]), the transport of lumefantrine and mefloquine is a high-affinity, low-capacity process.

Lumefantrine and mefloquine were shown to inhibit the transport of a natural substrate of PfCRT (the hemoglobin-derived hexapeptide VDPVNF [10]) via all 3 isoforms (Fig 6C and 6F). However, all of the resulting IC_50_s were above 45 μM, which is substantially higher than the IC_50_s previously obtained for the quinine dimer “Q_2_C” (IC_50_s = 0.01 to 0.6 μM)—a drug that has been established as exerting an antiplasmodial effect via its potent inhibition of PfCRT’s natural function [10,92]. The lumefantrine and mefloquine IC_50_s are also significantly higher than those observed for 2 other inhibitors of PfCRT—verapamil (IC_50_s = 6.8 to 17.5 μM) and chlorpheniramine (IC_50_s = 5.0 to 9.8 μM)—but are very similar to the IC_50_s obtained with chloroquine (e.g., 110 ± 5.9 μM against PfCRT^3D7^ and 53 ± 2.8 μM against PfCRT^Dd2^) [10]. It seems unlikely, therefore, that lumefantrine and mefloquine possess significant anti-PfCRT activity.

We then tested whether PfCRT^7G8^ and PfCRT^Dd2^ transport lumefantrine and mefloquine within their native environment in the DV membrane of live parasites. The transport of lumefantrine and mefloquine via PfCRT was measured in situ using the “H^+^-efflux assay” with a set of *P*. *falciparum* transfectants—the C2^GC03^, C6^7G8^, and C4^Dd2^ lines—that are isogenic except for the *pfcrt* allele. These lines express either PfCRT^3D7^ (C2^GC03^), PfCRT^7G8^ (C6^7G8^), or PfCRT^Dd2^ (C4^Dd2^) [17]. Lumefantrine and mefloquine enter the DV via simple diffusion of the neutral species and also via PfMDR1 (Figs 2 and 3), where they become protonated in the acidic lumen. The H^+^-efflux assay uses a fluorescent pH-sensitive probe to provide an indirect method of detecting the efflux of protonated drugs from the DV via PfCRT, which manifests as an increase in the rate of DV alkalinization. The positive controls were chloroquine and saquinavir (a peptide mimic that is a substrate of all 3 PfCRT isoforms [10]). Consistent with previous reports [9,10,20–22], chloroquine increased the rate of DV alkalinization in the C6^7G8^ and C4^Dd2^ lines and was without effect in the C2^GC03^ line, whereas saquinavir increased the rate of DV alkalinization in all 3 parasite lines (Fig 6G). We found that lumefantrine and mefloquine induced a H^+^ leak in the C6^7G8^ and C4^Dd2^ lines, but not in the C2^GC03^ line. These observations are consistent with both drugs being substrates of PfCRT^7G8^ and PfCRT^Dd2^, but not PfCRT^3D7^ (Fig 6G–6J). Furthermore, verapamil inhibited the lumefantrine- and mefloquine-induced H^+^ leaks (Fig 6H), providing further evidence that the drug-induced efflux of H^+^ from the DV was mediated by PfCRT^7G8^ and PfCRT^Dd2^. Both drugs increased the rate of DV alkalinization in a concentration-dependent manner, with PfCRT^Dd2^ again exhibiting a higher capacity for lumefantrine and mefloquine transport than PfCRT^7G8^ (Fig 6I and 6J). Together, these findings support the datasets obtained with the *Xenopus* oocyte system by providing an in situ demonstration that lumefantrine and mefloquine accumulate within the DV and that chloroquine resistance-conferring isoforms of PfCRT transport both of these drugs back out into the parasite’s cytosol.

If lumefantrine and mefloquine exert their primary antimalarial activities on targets outside of the DV, the PfCRT-mediated efflux of these drugs from the DV of the C6^7G8^ and C4^Dd2^ lines should heighten parasite sensitivity to lumefantrine and mefloquine, and verapamil should reduce this effect. We tested this hypothesis by measuring the susceptibility of the isogenic parasite lines to lumefantrine, mefloquine, and chloroquine in the presence or absence of verapamil. The resulting IC_50_s confirmed that the C6^7G8^ and C4^Dd2^ lines are more susceptible to lumefantrine and mefloquine than the C2^GC03^ parasites (Table 1), a finding that is consistent with previous reports [2,17]. Furthermore, verapamil decreased the sensitivity of the C6^7G8^ and C4^Dd2^ lines to lumefantrine and mefloquine, while simultaneously increasing their sensitivity to chloroquine. Together, our in situ datasets show that chloroquine resistance-conferring isoforms of PfCRT can mediate the transport of lumefantrine and mefloquine from the DV into the parasite cytosol and that this “gain of transport function” underlies the heightened sensitivity of chloroquine-resistant parasites to lumefantrine and mefloquine.

**Table 1:** In vitro antiplasmodial activities of chloroquine, lumefantrine, and mefloquine against *P*. *falciparum pfcrt* transfectant lines.

Parasite line	IC_50_ (nM)^a^					
	Chloroquine		Lumefantrine		Mefloquine	
	Control	+ verapamil	Control	+ verapamil	Control	+ verapamil
C2^GC03^	25 ± 1.1	26 ± 1.7	71 ± 3.5	68 ± 2.5	52 ± 1.7	50 ± 2.7
C6^7G8^	84 ± 3.5^b^	27 ± 1.0^c^	42 ± 1.6^b^	69 ± 1.8^c^	29 ± 1.2^b^	53 ± 2.1^c^
C4^Dd2^	132 ± 4.9^b^	28 ± 1.1^c^	45 ± 1.8^b^	67 ± 1.9^c^	32 ± 1.8^b^	50 ± 1.9^c^

^a^The IC_50_ values are the mean ± SEM of 4 independent experiments (performed on different days), within which measurements were averaged from 3 replicates.

^b^The *P* values determined from a 1-way ANOVA were less than 0.001 for comparisons with the C2^GC03^ line, within a control treatment.

^c^The *P* values determined from a 1-way ANOVA were less than 0.001 for comparisons between the control treatment and the relevant + verapamil treatment within the same line.

The data underlying this figure is supplied in S3 Data.

## Discussion

The roles of PfMDR1 in the phenomenon of antimalarial drug resistance have remained unclear for several decades, despite it being one of the key determinants of multidrug resistance in the parasite. Our development of a robust expression system for PfMDR1 in *Xenopus* oocytes has enabled direct and detailed characterizations of its capacity for drug transport, and of how this varies between different field isoforms of the transporter. Using this system, we have provided new fundamental insights into the function of PfMDR1, such as its ability to transport a wide range of structurally diverse drugs that includes most of the antimalarials currently in use. We found that the mutant isoforms of PfMDR1 differed from the wild-type protein, and each other, in their capacities to transport these drugs. Furthermore, in almost all cases, the introduction of mutations into PfMDR1 decreased its capacity for drug transport. In the parasite, the reduced capacities of the mutant isoforms for drug transport will result in less of the drug entering and accumulating within the DV, and thus a greater proportion of the drug will remain in the cytosol. By contrast, the overexpression of PfMDR1 (which increases the level of PfMDR1 at the DV membrane [93,94]) will heighten the rate of drug transport into the DV, thereby increasing the sequestration of drugs within this organelle. These findings suggest that changes in PfMDR1 contribute to multidrug resistance phenotypes primarily by altering the distribution of drugs between the parasite cytosol and the DV.

We delved into this possibility further by examining the mechanistic basis for the patterns of collateral drug sensitivity induced by polymorphisms in *pfmdr1* and *pfcrt*. We focused upon characterizing the interactions of chloroquine, lumefantrine, and mefloquine with PfMDR1, and of lumefantrine and mefloquine with PfCRT (given that chloroquine transport via PfCRT has already been extensively studied in the *Xenopus* oocyte system and in situ [6,7,9,20,21]). Kinetic analyses confirmed that wild-type PfMDR1 possesses the highest capacity for the transport of lumefantrine, mefloquine, and chloroquine and that all of the mutant isoforms included for study exhibit reduced capacities for the transport of these drugs. In all cases, the PfMDR1^NFCDY^ isoform (carried by the chloroquine-resistant strain 7G8) exhibited the lowest capacity for drug transport. We observed a very different relationship between mutations in PfCRT and the protein’s capacity for drug transport. Wild-type PfCRT was found to lack the ability to transport lumefantrine or mefloquine in both the *Xenopus* oocyte system and a complementary in situ assay. By contrast, mutant isoforms of PfCRT (carried by the chloroquine-resistant strains 7G8 and Dd2) displayed significant capacities for the transport of these 2 drugs. That said, relative to chloroquine transport via PfCRT^Dd2^ [7], the mutant proteins are high-affinity, low-capacity transporters of lumefantrine and mefloquine. In the parasite, the mutant transporters will mediate the efflux of chloroquine, lumefantrine, or mefloquine from the DV back into the parasite’s cytosol, thereby decreasing the concentration of these drugs within the DV. By contrast, little or no drug will exit the DV of parasites carrying wild-type PfCRT, which means that chloroquine, lumefantrine, and mefloquine will continue to sequester within this compartment.

Given that PfMDR1 and PfCRT are potential drug targets themselves [1,95], we investigated whether they might be potently blocked by lumefantrine, mefloquine, or chloroquine. However, all 3 antimalarials were relatively poor inhibitors of transport via PfMDR1 and all 3 drugs also lacked potency against PfCRT; chloroquine [10], lumefantrine, and mefloquine were (similarly) weak inhibitors of the transport of a natural substrate via PfCRT. It is therefore unlikely that the natural functions of PfMDR1 and PfCRT are targeted to a significant extent by chloroquine, lumefantrine, or mefloquine.

Having obtained rates for the transport of lumefantrine, mefloquine, and chloroquine via 6 field isoforms of PfMDR1, we sought to identify relationships between capacities for drug transport and the in vitro drug responses of parasites carrying these PfMDR1 isoforms. We observed positive correlations between the rates of lumefantrine and mefloquine transport via PfMDR1 and the corresponding in vitro drug responses (see S3 Text for an extended discussion). For example, PfMDR1 isoforms with relatively high capacities for mefloquine transport tended to be present in the parasites with relatively high mefloquine IC_50_s (and thus increased resistance to the drug), whereas isoforms with low capacities for mefloquine transport tended to be present in the parasites with low mefloquine IC_50_s (and thus increased sensitivity to the drug). We observed the opposite relationship for chloroquine, with the chloroquine transport capacities of the PfMDR1 field isoforms correlating negatively with the corresponding in vitro chloroquine responses (see S3 Text for an extended discussion). These analyses identify the rate of drug transport via PfMDR1 as being a key contributor to the collateral drug sensitivity patterns observed for lumefantrine, mefloquine, and chloroquine in field isolates and laboratory-adapted strains. In parasites carrying a mutant PfMDR1 isoform, the decrease in the rate of drug transport into the DV will lessen the accumulation of lumefantrine, mefloquine, and chloroquine within this organelle and cause a concomitant increase in the cytosolic concentrations of these 3 drugs. By contrast, the overexpression of PfMDR1 would increase the rate of drug transport into the DV, leading to the heightened sequestration of lumefantrine, mefloquine, and chloroquine within the DV and a reduction in the cytosolic concentrations of these drugs. These findings, together with our previous demonstration of a positive correlation between the chloroquine transport capacities of field isoforms of PfCRT and the in vitro chloroquine responses of the corresponding parasites [7], reveal that the redistribution of drugs between the DV and the cytosol is the key mechanism by which polymorphisms in *pfmdr1* and *pfcrt* cause collateral drug sensitivity.

**Fig 7:**
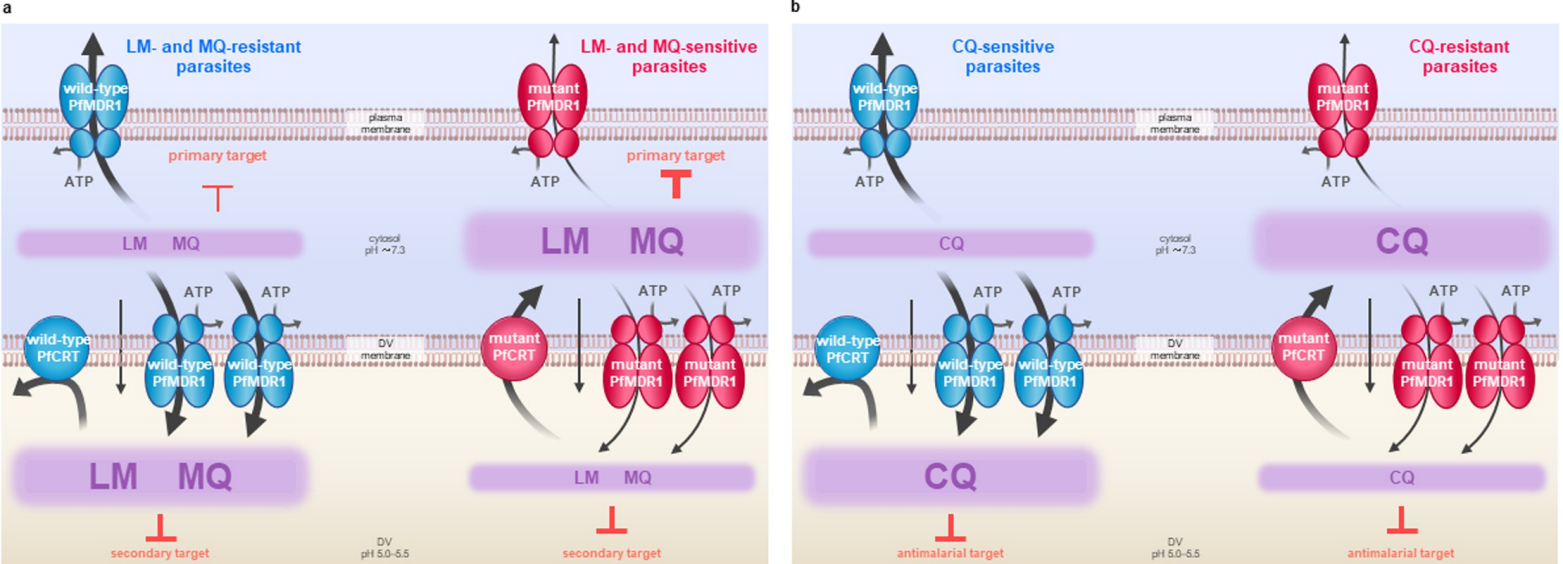
Roles of PfMDR1 and PfCRT in the parasite’s susceptibility to lumefantrine, mefloquine, and chloroquine. Mechanistic explanations for how polymorphisms in *pfmdr1* and *pfcrt* alter the parasite’s response to lumefantrine and mefloquine **(a)** and chloroquine **(b)**. Lumefantrine, mefloquine, and chloroquine are weak bases that enter the DV via 2 main routes: (1) simple diffusion of the neutral species across the membrane and subsequent protonation within the acidic DV lumen; and (2) ATP-driven import via PfMDR1. Wild-type PfMDR1 has a high capacity for drug transport and this activity, together with the inability of wild-type PfCRT to efflux lumefantrine, mefloquine, or chloroquine from the DV, causes these drugs to sequester within the DV. Overexpression of PfMDR1 results in a further increase in the rate of drug transport from the cytosol into the DV and thus greater accumulation of lumefantrine, mefloquine, and chloroquine in the DV (as well as concomitant reductions in their cytosolic concentrations). In parasites carrying a mutant PfMDR1 isoform (and/or only one *pfmdr1* copy) as well as a mutant isoform of PfCRT, there is a marked reduction in the DV accumulation of lumefantrine, mefloquine, and chloroquine as a result of (1) a decrease in the rate of drug import via PfMDR1; and (2) the PfCRT-mediated efflux of drugs from the DV back into the cytosol. The reduction in the concentration of chloroquine at its primary site of action allows the parasite to evade its killing effects, thereby causing chloroquine resistance. On the other hand, the concomitant increases in the cytosolic drug concentrations render these parasites more sensitive to lumefantrine and mefloquine, indicating that the primary targets of both drugs are located outside of the DV. CQ, chloroquine; DV, digestive vacuole; LM, lumefantrine; MQ, mefloquine; PfCRT, *Plasmodium falciparum* chloroquine resistance transporter; PfMDR1, *Plasmodium falciparum* multidrug resistance protein 1.

The detailed biochemical characterizations of PfMDR1 and PfCRT we have presented here enable the construction of a mechanistic model for collateral drug sensitivity in the malaria parasite (Fig 7 and S3 Text). Lumefantrine, mefloquine, and chloroquine enter the DV, and accumulate therein, via 2 main routes: (1) simple diffusion of the neutral species across the membrane and subsequent protonation within the acidic DV lumen; and (2) ATP-driven import via PfMDR1. Wild-type PfMDR1 has a relatively high capacity for drug transport and this activity, together with the inability of wild-type PfCRT to efflux lumefantrine, mefloquine, or chloroquine from the DV, causes these drugs to accumulate to high levels within the DV. Overexpression of PfMDR1 results in a further increase in the rate of drug transport from the cytosol into the DV and thus greater sequestration of lumefantrine, mefloquine, and chloroquine in the DV (as well as concomitant reductions in their cytosolic concentrations). Hence, parasites carrying wild-type *pfmdr1* (and/or multiple copies of *pfmdr1*) as well as wild-type *pfcrt* are chloroquine-sensitive because chloroquine accumulates to high levels at its primary site of antimalarial action—i.e., the detoxification of heme within the DV [96–98]. Given that these parasites exhibit reduced, rather than increased, susceptibilities to lumefantrine and mefloquine, our model indicates that these 2 drugs exert their primary antimalarial effects outside of the DV (mefloquine is thought to target the parasite’s cytoplasmic 80S ribosome [50] and the primary target of lumefantrine remains unknown). In parasites carrying a mutant PfMDR1 isoform (and/or only one *pfmdr1* copy) as well as a mutant isoform of PfCRT, there is a marked reduction in the DV accumulation of lumefantrine, mefloquine, and chloroquine as a result of (1) a decrease in the rate of drug import via PfMDR1; and (2) the PfCRT-mediated efflux of drugs from the DV back into the cytosol. The reduction in the concentration of chloroquine at its primary site of action allows the parasite to evade its killing effects, thereby causing chloroquine resistance. On the other hand, the concomitant increases in the cytosolic drug concentrations render these parasites more sensitive to lumefantrine and mefloquine. The mechanistic model we have presented here concurs with previously proposed hypotheses of an interplay between PfMDR1 and PfCRT in modulating the parasite’s susceptibility to these drugs [2–4,15,99,100]. Our model also suggests that the targets previously proposed for mefloquine and lumefantrine inside the DV (such as the detoxification of heme) play secondary roles, or perhaps no role, in the antimalarial activities of these drugs.

Our datasets also provide insights into why some pairings of PfMDR1 and PfCRT isoforms result in parasites with unexpectedly low or high susceptibilities to chloroquine [7]. Mutations in *pfcrt* are the primary determinant of chloroquine resistance and, according to the model we have proposed, the extent to which polymorphisms in *pfmdr1* contribute to a given chloroquine resistance phenotype will depend on several factors. These include (1) the rate of chloroquine efflux via the mutant PfCRT isoform expressed by the parasite; (2) the rate of chloroquine entry into the DV via simple diffusion (which is dependent on the concentration of chloroquine in the parasite cytosol and, in turn, the extracellular concentration of chloroquine); (3) the rate of chloroquine import via the PfMDR1 isoform(s) expressed by the parasite; and (4) the level of PfMDR1 expression at the DV membrane. For example, the combination of a mutant PfCRT isoform that mediates a high rate of chloroquine efflux (e.g., PfCRT^Dd2^) and a mutant PfMDR1 isoform with a decreased capacity for chloroquine import could be expected to achieve a somewhat lower DV concentration of the drug (and thus perhaps a higher level of resistance) than would be brought about by the transport activity of PfCRT alone. However, the ability of PfMDR1 to influence the parasite’s susceptibility to chloroquine is perhaps more evident when the mutant PfCRT has a relatively low capacity for chloroquine efflux (e.g., PfCRT^7G8^). We have previously reported that 7G8 parasites exhibit a greater level of chloroquine resistance than what would be predicted given the low level of chloroquine transport mediated by PfCRT^7G8^ [7]. Here, our finding that PfMDR1^NFCDY^ (the isoform carried by 7G8 parasites) has a very low capacity for chloroquine transport provides a mechanistic explanation for this observation; the marked reduction in the rate of chloroquine import via PfMDR1^NFCDY^ helps offset the relatively low rate of chloroquine efflux via PfCRT^7G8^, resulting in a lower DV concentration of chloroquine (and thus higher level of resistance) than would be achieved by PfCRT^7G8^ with wild-type PfMDR1 (or even with one of the other mutant PfMDR1 isoforms). Hence, our datasets suggest that PfMDR1^NFCDY^ has the potential to contribute to chloroquine resistance phenotypes to a greater extent than other PfMDR1 isoforms as a consequence of its very low capacity for chloroquine transport.

Several lines of evidence indicate that mutant isoforms of PfCRT play a key role in conferring quinine resistance [9,18,22,37,92], and we have previously used the *Xenopus* oocyte system to show that mutant isoforms of PfCRT have the ability to transport quinine out of the DV, whereas the wild-type protein lacks this activity [9,18] (see S4 Text for an extended analysis). Collectively, these findings indicate that PfCRT’s role in quinine resistance is similar to its role in chloroquine resistance (Fig 7). That is, the efflux of quinine from the DV into the parasite cytosol via a mutant PfCRT isoform reduces the concentration of the drug at its DV target (the inhibition of heme detoxification and/or another aspect of hemoglobin digestion [101]), thereby reducing the susceptibility of the parasite to quinine. It might be predicted, therefore, that the role of PfMDR1 would likewise be similar in both chloroquine and quinine resistance. Indeed, our datasets showed that most of the mutant PfMDR1 isoforms have low capacities for quinine transport relative to the wild-type protein, and our observations are consistent with previous findings of associations between mutations in PfMDR1 and decreases in the parasite’s susceptibility to quinine [70,102]. However, our analysis of the relationship between the quinine transport capacities of PfMDR1 and the quinine resistance indices for the corresponding parasites did not identify a correlation between these 2 factors. This is perhaps a reflection of a complex and multifactorial pathway to quinine resistance that may sometimes involve polymorphisms in *pfmdr1* and other times not. Indeed, when considered in context with a large body of previous research (see S4 Text for an extended discussion), our findings indicate that the role of PfMDR1 in quinine resistance is more complex and nuanced than simply either causing a decrease or an increase in the accumulation of the drug within the DV (i.e., the roles we have proposed for PfMDR1 in chloroquine resistance and mefloquine/lumefantrine resistance, respectively). Our analyses instead suggest that the complex and variable relationship between quinine susceptibility and polymorphisms in *pfcrt* and *pfmdr1* may center on 2 key factors: (1) the net flux of quinine across the DV membrane; and (2) the rate at which PfMDR1’s natural substrates are transported into the DV (see S4 Text for an extended discussion).

The robust system we have established for the study of PfMDR1 in *Xenopus* oocytes provides the long-awaited means to undertake direct and detailed characterizations of its function and to make comparisons between different PfMDR1 isoforms and across different drugs. Our findings have delivered fundamental insights into PfMDR1 that have led to a greater understanding of its role in the phenomenon of multidrug resistance as well as the formulation of a mechanistic model for PfMDR1’s contribution to collateral drug sensitivity in the malaria parasite. Together, the work presented here provides a valuable molecular basis for the rational design of approaches that maintain and extend the useful life span of current antimalarials by exploiting the opposing selection forces they exert upon PfCRT and PfMDR1. Looking ahead, our systems could be employed to address unresolved questions about PfMDR1 and PfCRT, such as the identity of the natural substrate(s) of PfMDR1, the functions and roles of the new PfMDR1 and PfCRT isoforms that continue to emerge in the field in response to changes in drug pressure, and the nature of the interactions of PfMDR1 and PfCRT with candidate antimalarials in the development pipeline.

## Methods

### *Xenopus laevis* frogs

Ethical approval of the work performed with female *X*. *laevis* frogs was obtained from the Australian National University Animal Experimentation Ethics Committee (Animal Ethics Protocol Numbers A2013/13 and A2019/26) in accordance with the Australian Code of Practice for the Care and Use of Animals for Scientific Purposes. The frogs were purchased from Nasco USA (catalog no. LM00535M) and were housed in the *Xenopus* Frog Facility (at the Australian National University Research School of Biology) in compliance with the relevant institutional and Australian Government regulations. *X*. *laevis* frogs are fully aquatic animals held in large tanks (8 to 20 frogs per tank). The water is percolated and filtered in a closed system, and the water temperature is maintained at 19 to 20°C. The pH, hardness, ammonia, and other water parameters were checked daily and kept at levels reported to be necessary for the production of healthy oocytes [103,104].

### Preparation of coding sequences and cRNA synthesis

The coding sequence of human P-gp was purchased from GenScript (catalog no. OHu23307; NM_000927; New Jersey USA) and inserted into the pGEM-He-Juel oocyte expression vector [105]. The PfMDR1^NYSND^ coding sequence was codon-harmonized to facilitate the correct folding, and thus the functional expression, of the transporter in *Xenopus* oocytes. This codon-harmonized sequence was custom synthesized and then inserted into the pGEM-He-Juel oocyte expression vector by GenScript. The remaining isoforms of PfMDR1, as well as the version of PfMDR1^NYSND^ carrying a double carboxyl-terminal HA tag (PfMDR1^NYSND^-2xHA), were generated from the PfMDR1^NYSND^ template using site-directed mutagenesis [7] and the primers listed in S4 Table. The resulting PfMDR1 coding sequences were verified by sequencing (undertaken by the ACRF Biomolecular Resource Facility, Australian National University). Amino-terminally HA-tagged versions of these PfMDR1 variants, as well as the version of PfMDR1^NYSND^ that carries a triple HA tag at the carboxyl terminus (PfMDR1^NYSND^-3xHA), were generated by GenScript.

The oocyte expression vectors containing PfNT1, PfCRT^3D7^, PfCRT^Dd2^, or PfCRT^7G8^ were made previously [6,7,106]. The PfCRT coding sequences were codon-harmonized and encode a version of PfCRT that is free of endosomal–lysosomal trafficking motifs, thereby resulting in the expression of the transporter at the plasma membrane of *Xenopus* oocytes [6,7].

The plasmids containing human P-gp, PfMDR1, PfCRT, or PfNT1 coding sequences were linearized with SalI (Thermo Fisher Scientific, Massachusetts, USA; catalog no. ER0642), and 5′-capped cRNA was synthesized using the mMESSAGE mMACHINE T7 transcription kit (Ambion, Texas, USA; catalog no. AM1344). The in vitro transcription reactions were conducted as per the manufacturer’s instructions with the following minor modification for the synthesis of human P-gp and PfMDR1 cRNA: in a 40 μL reaction, 500 ng of linearized template plasmid was used, and 30 nmol GTP was added halfway into the 2-hour incubation. The resulting cRNA samples were purified with the MEGAclear kit (Ambion, catalog no. AM1908) and adjusted to the desired concentration using RNase-free elution buffer. The quality of the cRNA was assessed via agarose gel electrophoresis.

### Isolation and microinjection of *Xenopus* oocytes

Adult female frogs were anaesthetized in a solution of ethyl 3-aminobenzoate methanesulfonate salt (Sigma-Aldrich, Missouri, USA; catalog no. A5040) and 1 mM NaHCO_3_ (Sigma-Aldrich, catalog no. S5761) [8]. Sections of the ovary were removed from the frog using surgical grade forceps and scissors. The ovary sections were cut into pieces containing approximately 20 oocytes, transferred to a 100 mL Erlenmeyer flask, and then gently rinsed 5 times with “calcium-free oocyte ringer” buffer (OR2^−^; 82.5 mM NaCl, 2.5 mM KCl, 1 mM MgCl_2_, 1 mM Na_2_HPO_4_, 5 mM HEPES; pH 7.8). The collagenous membrane encasing the oocytes was degraded by incubating the ovary sections in 25 mL of OR2^−^ buffer supplemented with 0.5 M Na_2_HPO_4_ (Sigma-Aldrich, catalog no. 342483), bovine serum albumin (250 mg/mL; Sigma-Aldrich, catalog no. A7030), and collagenase B (Roche, Basel, Switzerland; catalog no. 11088831001) for 12 to 13 hours at 16°C on an orbital shaker. The speed of the orbital shaker was adjusted according to the volume of ovary sections in the flask, such that the ovary sections were circulating gently around the flask (shaker speeds that are too low will result in incomplete defolliculation, whereas speeds that are too high cause damage to the plasma membrane of the oocytes). The amount of collagenase B added to the solution was dependent on the amount of collagen surrounding the ovary sections, which was assessed by visual inspection under a dissecting microscope. The collagenase-treated oocytes were washed 10 times with OR2^−^ buffer, 5 times with “calcium-containing oocyte ringer” (OR2^+^) buffer (OR2^−^ buffer supplemented with 1 mM CaCl_2_ and 50 μg/mL penicillin and streptomycin), and then once with L-15 (Leibovitz) media with L-glutamine (used at 0.5×; Sigma-Aldrich, catalog no. L4386) supplemented with 10 mM HEPES and 50 μg/mL penicillin and streptomycin. Stage V and VI oocytes were microinjected with cRNA encoding a PfMDR1 isoform (10 ng per oocyte), a PfCRT isoform (20 ng per oocyte), human P-gp (10 ng per oocyte, these oocytes served as a positive control), or PfNT1 (20 ng per oocyte, these oocytes served as a negative control). In a subset of experiments that were performed to optimize the amount of cRNA required for human P-gp or PfMDR1 expression, oocytes were microinjected with cRNA amounts ranging from 2.5 to 50 ng. The oocytes were stored at 16 to 18°C in L-15 media supplemented with 10 mM HEPES and 50 μg/mL penicillin and streptomycin.

### Radiolabeled and unlabeled drugs used in the *Xenopus* oocyte transport assays

The radiolabeled drugs were purchased from either Pharmaron (Beijing, China; [^3^H]chloroquine, 27 Ci/mmol; [^3^H]dihydroartemisinin, 14 Ci/mmol), American Radiolabeled Chemicals (Missouri, USA; [^3^H]lumefantrine, 10 Ci/mmol; [^3^H]mefloquine, 20 Ci/mmol; [^3^H]amodiaquine, 15 Ci/mmol; [^3^H]piperaquine, 15 Ci/mmol; [^3^H]quinine, 20 Ci/mmol; [^3^H]quinidine, 20 Ci/mmol; [^3^H]vinblastine sulfate, 20 Ci/mmol), Moravek (California, USA; [^3^H]amantadine, 137 mCi/mmol), Cambridge Research Biochemicals (Billingham, UK; [^3^H]VDPVNF, 20 Ci/mmol), or PerkinElmer (Massachusetts, USA; [^3^H]hypoxanthine, 13.3 Ci/mmol). The structures and protonation states of these drugs are shown in S8 Fig and S2 Data, respectively. The unlabeled compounds were purchased from either Sigma-Aldrich (lumefantrine, mefloquine, chloroquine, quinine, quinacrine, rhodamine B, methylene blue, amantadine, nicardipine, vanadate, verapamil, PSC833, chlorpheniramine, saquinavir, histidine, and iron chelator IV) or GenScript (the peptides LH and VDPVNF). The structures of a subset of these compounds are shown in S8 Fig.

### Measurements of drug transport via human P-gp and PfMDR1 in *Xenopus* oocytes

The human P-gp and PfMDR1 transport assays were performed 1 day post-cRNA injection and, unless specified otherwise, were conducted over 1.5 hours at 27.5°C in ND96 buffer (96 mM NaCl, 2 mM KCl, 1 mM MgCl_2_, 1.8 mM CaCl_2_, 10 mM MES, and 10 mM Tris-base) that had been adjusted to pH 5.5 to mimic the pH of the DV lumen [107,108]. Since the ATPase domains of ABC transporters (such as PfMDR1 and human P-gp) are located in the cell’s cytosol [36], both PfMDR1 and human P-gp are predicted to orientate in the oocyte plasma membrane with their ATPase domains in the oocyte cytosol. An established efflux assay [10] was therefore used to microinject the radiolabeled drug under study into the oocyte and measure its efflux via human P-gp or PfMDR1. In this assay, the transport of the substrate via human P-gp from the oocyte cytosol into the extracellular solution is analogous to the P-gp–mediated transport of a substrate from the cytosol of a human cell into the extracellular space. Likewise, for PfMDR1 the direction of transport is analogous to the PfMDR1-mediated transport of a substrate from the parasite’s cytosol into the DV (S9 Fig). In all cases, 4 independent experiments were performed (on different days and using oocytes from different frogs) and within each experiment measurements were made from 10 oocytes per treatment.

The oocytes were microinjected with 35 nL of ND96 buffer (pH 8.0) supplemented with the [^3^H]drug under study. The estimated intracellular concentrations (calculated on the basis that the aqueous volume of stage V andVI oocytes is 400 nL [109]) were as follows: [^3^H]vinblastine sulfate, 4.4 μM; [^3^H]lumefantrine, 2.2 μM; [^3^H]chloroquine, 5.9 μM; [^3^H]quinine, 4.4 μM; [^3^H] mefloquine, 4.4 μM; [^3^H]amodiaquine, 3.1 μM; [^3^H]piperaquine, 3.1 μM; [^3^H]dihydroartemisinin, 3.1 μM; [^3^H]quinidine, 4.4 μM; [^3^H]amantadine, 30 μM. In the experiments measuring [^3^H]dihydroartemisinin transport, 1 mM iron chelator IV was also microinjected into the oocyte to bind any free iron present in the oocyte (free iron could activate dihydroartemisinin, which, in turn, could damage the oocytes and thus interfere with measurements of transport; S10 Fig). Ten oocytes that had resealed at the injection site were transferred to a 5 mL polystyrene round-bottom tube (Corning, New York, USA; catalog no. 352008) and washed once with 3.5 mL of ND96 buffer (pH 5.5) before being transferred to separate wells of a white 96-well plate (PerkinElmer, catalog no. 6005299). This treatment determined the amount of radioactivity within the oocytes at the beginning of the assay (T = 0). Another 10 resealed oocytes were transferred to a 5-mL tube, washed once with 3.5 mL of ND96 buffer (pH 5.5), and suspended in 100 μL of ND96 buffer (pH 5.5) to commence the assay. The incubation was terminated by removing the reaction buffer with a pipette and washing the oocytes twice with 3.5 mL of ice-cold ND96 buffer (pH 5.5). Each oocyte was transferred to a separate well of a white 96-well plate, lysed by an overnight incubation at room temperature with 20 μL of 10% (w/v) SDS, and then combined with 150 μL of MicroScint-40 microscintillant (PerkinElmer, catalog no. 6013641). The plate was covered with a TopSeal-A (PerkinElmer, catalog no. 6050185), and the radioactivity within each well was measured with a MicroBeta^2^ microplate liquid scintillation analyzer (PerkinElmer, catalog no. 2450–0010). Drug transport was calculated by subtracting the amount of radioactivity (measured in counts per min) present within the oocytes at the end of the incubation from that measured in the oocytes sampled immediately before the commencement of the assay.

The transport of methylene blue, quinacrine, and rhodamine B via PfMDR1 was measured using the intrinsic fluorescence of these compounds and a fluorescence-based transport assay. The oocytes were microinjected with 35 nL of ND96 buffer (pH 8.0) supplemented with the fluorescent compound under study. The estimated intracellular concentration (calculated on the basis that the aqueous volume of stage V and VI oocytes is 400 nL [109]) for quinacrine, rhodamine B, or methylene blue was 4 μM. Of the oocytes that had resealed at the injection site, 10 were transferred to a 5-mL polystyrene round bottom tube and washed once with 3.5 mL of ND96 buffer (pH 5.5) before being transferred to separate wells of a clear 96-well plate. This treatment determined the amount of fluorescence within the oocytes at the beginning of the assay (T = 0). Another 10 resealed oocytes were transferred to a 5-mL tube, washed once with 3.5 mL of ND96 buffer (pH 5.5), and suspended in 100 μL of ND96 buffer (pH 5.5) to commence the assay. The incubation was terminated by removing the reaction buffer with a pipette and washing the oocytes twice with 3.5 mL of ice-cold ND96 buffer (pH 5.5). Each oocyte was transferred to a separate well of a clear 96-well plate. In addition, the autofluorescence of oocytes that had not been microinjected with a drug was measured by transferring 10 oocytes to a 5-mL tube and washing them 3 times with 3.5 mL of ND96 buffer (pH 5.5) before transferring them to separate wells of a clear 96-well plate. The oocytes were lysed by an overnight incubation at room temperature with 20 μL of 10% (w/v) SDS. The fluorescence intensity in each well was measured using an Infinite M1000 PRO plate reader (Tecan, Mannedorf, Switzerland; catalog no. 30064852) at the following excitation and emission wavelengths: 440 nm and 510 nm for quinacrine; 546 nm and 568 nm for rhodamine B; 668 nm and 682 nm for methylene blue. Drug transport was calculated by first subtracting the autofluorescence measured from all treatments, and then subtracting the fluorescence present within the oocytes at the end of the incubation from that measured in the oocytes sampled immediately before the commencement of the assay.

A subset of assays measured the ability of unlabeled compounds to *cis*-inhibit [^3^H]drug transport via human P-gp and PfMDR1. In these experiments, the oocytes were microinjected with 35 nL of the relevant [^3^H]drug solution supplemented with the unlabeled test compound at a concentration that would achieve the desired intracellular concentration (0.1 to 30 μM). The test solutes included known inhibitors of human P-gp (nicardipine [46,54], PSC833 [55,56], vanadate [57,58], and verapamil [46,59]), and a compound that does not interact with human P-gp (the antiviral drug amantadine [46–48]). The abilities of unlabeled lumefantrine, mefloquine, and chloroquine (intracellular concentrations of 1 to 200 μM) to *cis*-inhibit [^3^H]vinblastine transport via PfMDR1 were also assessed. The IC_50_ value of each inhibitor was determined in GraphPad Prism Version 8 by a least squares fit of the equation y = y_min_ + [(y_max_ − y_min_)/(1 + ([test compound]/IC_50_)^*c*^] to the data, where y is P-gp–or PfMDR1-mediated drug transport, y_min_ and y_max_ are the minimum and maximum values of y, and *c* is a fitted constant.

The ATP dependence of lumefantrine transport via PfMDR1 was determined by microinjecting the oocytes with [^3^H]lumefantrine suspended in 35 nL of ND96 buffer (pH 8.0) containing different concentrations of ATP. Unfertilized stage V and VI oocytes typically contain approximately 2.3 mM ATP [110,111] and microinjection of the different ATP solutions is calculated to have raised the intracellular concentration by 0 to 2 mM.

Analyses of the kinetics of drug transport via PfMDR1 were undertaken by microinjecting the oocytes with ND96 buffer (pH 8.0) supplemented with the [^3^H]drug under study and different concentrations of the unlabeled drug (to achieve intracellular concentrations between 0 and 100 μM). The kinetic parameters for drug transport via different isoforms of PfMDR1 were determined in GraphPad Prism Version 8 by a least squares fit of the Michaelis–Menten equation to the data.

The experiments measuring drug transport via human P-gp or PfMDR1 included ne and those expressing PfNT1 [60,61] (an unrelated transporter) as negative controls. The latter control verified that the integrity of the oocyte plasma membrane had not been compromised (i.e., that the membrane was not leaky) from the combined effects of the heterologous expression of a foreign protein and the second injection event. To confirm that PfNT1 was expressed at the oocyte surface, the uptake of [^3^H]hypoxanthine (a known substrate of PfNT1) was measured in ne and those expressing PfNT1 on day 1 post-cRNA injection. Briefly, 10 oocytes were washed twice with 3.5 mL of ND96 buffer (pH 6.0), and the residual solution was removed by pipette. The assay commenced with the addition of 100 μL ND96 buffer (pH 6.0) containing 0.38 μM [^3^H]hypoxanthine and the incubation period was 30 minutes. The assay was terminated by removing the reaction buffer and washing the oocytes twice with 3.5 mL of ice-cold ND96 buffer (pH 6.0). Each oocyte was transferred to a well of a white 96-well plate and the samples were processed, and the radioactivity measured, as described above.

### Measurements of drug transport via PfCRT in *Xenopus* oocytes

Unless specified otherwise, the PfCRT transport assays were performed 2 to 3 days post-cRNA injection and were conducted over 1.5 hours at 27.5°C. The orientation of PfCRT in the parasite’s DV membrane is such that its amino terminus and carboxyl terminus extend into the cytosol, and hence, the orientation of the protein is predicted to be the same in the oocyte plasma membrane. Hence, the transport of a substrate via PfCRT from the acidic extracellular solution into the oocyte cytosol [6] is analogous to the PfCRT-mediated efflux of a substrate out of the parasite’s acidic DV and into the cytosol [6] (S9 Fig). This is the direction in which mefloquine transport was measured in the PfCRT oocyte assays. Briefly, 10 oocytes were washed twice with 3.5 mL of ND96 buffer (pH 5.0), and the residual solution was removed by pipette. The assay commenced with the addition of 100 μL ND96 buffer (pH 5.0) containing 0.125 μM [^3^H]mefloquine and 0.5 μM unlabeled mefloquine and, where specified, 100 μM of either a known inhibitor of the transporter (verapamil [6,18], chlorpheniramine [90], or saquinavir [10,91]) or a compound that does not interact with PfCRT (histidine or the dipeptide LH [9,10]). Four independent experiments were performed (on different days and using oocytes from different frogs), and within each experiment measurements were made from 10 oocytes per treatment.

Analyses of the kinetics of mefloquine transport via PfCRT were undertaken by measuring the uptake of [^3^H]mefloquine (0.125 μM) in the presence of 0 to 50 μM unlabeled mefloquine. In all cases, the incubation was terminated by removing the reaction buffer and washing the oocytes twice with 3.5 mL of ice-cold ND96 buffer (pH 5.0). Each oocyte was transferred to a well of a white 96-well plate and the samples were processed, and the radioactivity measured, as described above. The component of transport attributable to PfCRT (i.e., PfCRT-mediated transport) was calculated by subtracting the level of [^3^H]mefloquine accumulation detected in the negative control oocytes (ne) from that measured in oocytes expressing PfCRT^3D7^, PfCRT^7G8^, or PfCRT^Dd2^. The kinetic parameters for mefloquine transport via PfCRT were determined in GraphPad Prism Version 8 by a least squares fit of the Michaelis–Menten equation to the data.

We have recently identified the native substrates of PfCRT as being host-derived peptides of 4 to 11 amino acid residues in length [10]. Moreover, we found that in addition to being H^+^-dependent, peptide transport via PfCRT requires a second cosubstrate that remains to be identified, but which is naturally present in the *Xenopus* oocyte. The PfCRT-mediated transport of the hemoglobin peptide VDPVNF was therefore quantified by microinjecting [^3^H]VDPVNF into the oocytes and measuring its efflux from the oocyte [10]. Moreover, after preliminary experiments revealed that [^3^H]lumefantrine is likewise reliant on the presence of the second cosubstrate for transport via PfCRT ([^3^H]lumefantrine uptake from the extracellular solution could not be detected in PfCRT-expressing oocytes), the transport of this drug was also measured using the efflux assay. Lumefantrine transport was measured by microinjecting oocytes with 25 nL of ND96 buffer (pH 5.5) supplemented with [^3^H]lumefantrine and unlabeled lumefantrine (to achieve estimated intracellular concentrations of 1.6 μM and 1 μM, respectively). The measurements of [^3^H]VDPVNF and [^3^H]lumefantrine transport via PfCRT were conducted as described above for the PfMDR1 efflux assays. Four independent experiments were performed (on different days and using oocytes from different frogs), and within each experiment measurements were made from 10 oocytes per treatment.

Analyses of the kinetics of lumefantrine transport via PfCRT were undertaken by microinjecting the oocytes with [^3^H]lumefantrine supplemented with different concentrations of unlabeled lumefantrine (to achieve estimated intracellular concentrations between 0 and 50 μM). The kinetic parameters for lumefantrine transport via PfCRT were determined in GraphPad Prism Version 8 by a least squares fit of the Michaelis–Menten equation to the data.

The ability of unlabeled verapamil, chlorpheniramine, saquinavir, histidine, or LH to *cis*-inhibit [^3^H]lumefantrine transport was assessed by supplementing the solution containing [^3^H]lumefantrine and unlabeled lumefantrine with the unlabeled test compound (to achieve an intracellular concentration of 100 μM).

The ability of unlabeled mefloquine or lumefantrine to *cis*-inhibit [^3^H]VDPVNF transport was assessed by microinjecting oocytes with 25 nL of ND96 buffer (pH 5.5) supplemented with [^3^H]VDPVNF and unlabeled VDPVNF (to achieve estimated intracellular concentrations of 1.4 μM and 200 μM, respectively) as well as the unlabeled drug at a concentration that would achieve the desired intracellular concentration (10 to 250 μM). The IC_50_ values were determined in GraphPad Prism Version 8 by a least squares fit of the equation y = y_min_ + [(y_max_ − y_min_)/(1 + ([test compound]/IC_50_)^*c*^] to the data, where y is PfCRT-mediated VDPVNF transport, y_min_ and y_max_ are the minimum and maximum values of y, and *c* is a fitted constant.

### Western blot analyses

Semiquantitative measurements of the level of HA-tagged PfMDR1 protein in the oocyte membrane 1 day post-cRNA injection were performed using an established method for obtaining preparations of oocyte membrane proteins [7]. The pellet containing the membrane protein fraction was solubilized in a solution comprising 10 mM dithiothreitol, 1.5% (v/v) SDS, 10% (v/v) β-mercaptoethanol, 9 mM Tris-HCl (pH 7.6), 4.5 mM NaCl, 0.45% (v/v) Triton X-100, and 27.5% (v/v) NuPage LDS sample buffer (Life Technologies, California, USA; catalog no. NP0007). The proteins present in the oocyte membrane preparations were separated in a NuPage 3 to 8% Tris-Acetate polyacrylamide gel (Thermo Fisher Scientific, catalog no. EA03752) using Tris-Acetate SDS running buffer, and then transferred to a 0.45 μM nitrocellulose blotting membrane (Bio-Rad, California, USA; catalog no. 1620115). The membranes were probed with mouse anti-HA antibody (1:5,000; Sigma-Aldrich, catalog no. H9658) followed by horseradish peroxidase-conjugated horse anti-mouse antibody (1:3,000; Cell Signaling Technologies, Massachusetts, USA; catalog. no 7076S). The band for each PfMDR1 isoform was detected with SuperSignal West Pico Chemiluminescent reagent (Thermo Fisher Scientific, catalog no. 34578), quantified using the ImageJ software [112], and expressed as a percentage of the band intensity measured for PfMDR1^NYSND^. Total protein staining was used to evaluate sample loading and efficiency of transfer [7,113]. Briefly, the nitrocellulose membranes were stained with Ponceau S for 5 minutes, then de-stained using ultrapure water. The stained protein bands were visualized using the Bio-Rad ChemiDoc MP Imaging System, and densitometric analyses were performed in Image Lab version 6.0.1 software (Bio-Rad) to quantify the summed intensity of all of the protein bands between 70 and 180 kDa (the region in which PfMDR1 is detected) within each lane. At least 4 independent experiments were performed (using oocytes from different frogs), and within each experiment, measurements were averaged from 2 separate replicates.

### Immunofluorescence assays

An immunofluorescence assay was used to localize HA-tagged PfMDR1 in oocytes 1 to 2 days post-cRNA injection using a method adapted from Richards and colleagues [9]. Unless otherwise specified, all incubation and wash steps were conducted at room temperature with gentle shaking or rotation. All incubations were performed using a volume of 500 μL, and all wash steps were performed using a volume of 1 mL. Six oocytes of each oocyte type were fixed with 4% (v/v) paraformaldehyde in PBS for 30 minutes. Oocytes were then washed 3 times for 10 minutes each in PBS and then permeabilized by incubation in 100% methanol at −20°C for 20 minutes without shaking. Another three 10-minute washes in PBS were performed before the oocytes were incubated for 2 hours in a blocking solution (4% (w/v) BSA, 2% (v/v) normal goat serum, and 0.1% (v/v) Triton X-100 in PBS). The solution was replaced with a second blocking solution (4% (w/v) BSA and 2% (v/v) normal goat serum in PBS) and incubated overnight at 4°C. The following day, the oocytes were incubated for an additional 4 hours at room temperature in the same blocking solution before incubation in a solution containing mouse anti-HA antibody (1:100; in a solution of PBS containing 1.5% (w/v) BSA and 0.01% (v/v) Triton X-100; Sigma-Aldrich, catalog no. H9568) for 4 hours at room temperature and then overnight at 4°C. The following day, the oocytes were washed 3 times for 10 minutes each in PBS supplemented with 1.5% (w/v) BSA. All of the remaining steps were performed in the dark at room temperature. The oocytes were incubated for 4 hours in a solution containing the Alexa Fluor 488 donkey anti-mouse antibody (1:500; in a solution of PBS containing 4% (w/v) BSA and 2% (v/v) normal goat serum; Molecular Probes, Oregon, USA; catalog no. A21202). The oocytes were then washed 3 times for 10 minutes each in PBS, fixed with 3.7% (v/v) paraformaldehyde in PBS for 30 minutes, and washed twice for 15 minutes each in PBS. The oocytes were dehydrated by a series of 15-minute incubations in solutions of increasing ethanol content: 30% (v/v) ethanol in PBS, 50% (v/v) ethanol in PBS, 70% (v/v) ethanol in ultrapure water, 90% (v/v) ethanol in ultrapure water, and 100% ethanol (3 incubations in 100% ethanol were performed). The dehydrated oocytes were embedded in acrylic resin using the Technovit 7100 plastic embedding system (Kulzer, Indiana, USA; catalog no. 64709003). Slices of 2 μm were obtained using a microtome with a glass knife, and the slices were dried on microscope slides. A drop of ProLong Gold Antifade Mountant (Life Technologies, catalog no. P36934) was placed on a #1.5 coverslip, which was then placed over the oocyte slices on each slide and sealed using clear nail polish. Images were captured using a Leica Sp5 inverted confocal laser microscope (Leica Microsystems, Wetzlar, Germany) using a 63× objective. Excitation was achieved with a 488-nm argon laser, and the emissions were captured using a 500- to 550-nm filter. Images were acquired using the Leica Application Suite Advanced Fluorescence Version 3.6 software (Leica Microsystems). At least 2 independent experiments were performed (on oocytes from different frogs) for each oocyte type, within which slices were examined from at least 5 oocytes.

### Orientation of PfMDR1 in the plasma membrane of *Xenopus* oocytes

To determine the orientation of PfMDR1 at the surface of *Xenopus* oocytes, an immunofluorescence assay was developed and performed on oocytes 1 day post-cRNA injection. This method used live oocytes that were not fixed or permeabilized, thus preventing the penetration of antibody into the oocyte cytosol. Hence, under these conditions, the antibody can only bind to cognate extracellular epitopes. All incubation steps were performed at 16 to 18°C in a volume of 500 μL with gentle shaking. The experiment included the following oocyte types: ne (the negative control), oocytes expressing HA_EL2_-AmDAT (the positive control), and oocytes expressing either 3xHA-PfMDR1^NYSND^ or PfMDR1^NYSND^-3xHA. Six oocytes from each type were incubated for 6 hours in a blocking solution (3% (w/v) BSA and 1% (v/v) normal goat serum in OR2^+^ buffer). This was followed by a 2-hour incubation with the mouse anti-HA antibody (1:250 in a solution of 1.5% (w/v) BSA in OR2^+^ buffer; Sigma-Aldrich catalog no. H9568). The samples were washed 3 times for 10 minutes each with OR2^+^ buffer and then incubated for 2 hours with the Alexa Fluor 488 donkey anti-mouse antibody (1:500 in a solution of 1.5% (w/v) BSA in OR2^+^ buffer; Molecular Probes, catalog no. A21202). The oocytes were then rinsed 3 times (10 minutes each) with OR2^+^ buffer. Imaging was performed using a Leica M205 FA fluorescence stereomicroscope and Leica Application Suite Version 4.12 software (Leica Microsystems). Excitation was achieved using a 488 nm laser and the emissions were captured using a 500- to 550-nm filter. At least 2 independent experiments were performed (using oocytes from different frogs) for each oocyte type.

### Culture of parasitized erythrocytes

The use of human blood for this work was approved by the ANU Human Research Ethics Committee (Human Ethics Approval Number 2017/351). The asexual intraerythrocytic stages of 3 *P*. *falciparum pfcrt* transfectant lines and 3 *P*. *falciparum* strains were studied. The 3 *pfcrt* transfectant lines were the chloroquine-sensitive line C2^GC03^ (a recombinant control that retains the wild-type *pfcrt* allele, PfCRT^3D7^) and the chloroquine-resistant lines C6^7G8^ and C4^Dd2^ (in which the wild-type *pfcrt* allele of the “GC03” line has been replaced with the mutant allele from the 7G8 or Dd2 strain, respectively [17]). A PCR error in the generation of the C6^7G8^ line introduced an additional mutation (I351M) [22], which is not found in the sequence of PfCRT^7G8^. The strains included 3D7 and HB3, which are chloroquine-sensitive parasites that carry PfCRT^3D7^, but express either PfMDR1^NYSND^ or PfMDR1^NFSDD^, respectively. The chloroquine-resistant strain was Dd2, which expresses PfCRT^Dd2^ and PfMDR1^FYSND^ and/or PfMDR1^YYSND^. The parasite cultures were maintained at 4% hematocrit and synchronized with 5% (w/v) sorbitol [114]. The *pfcrt* transfectant lines were maintained in the presence of the selection agents blasticidin (5 μM; Sigma-Aldrich, catalog no. 30891) and WR99210 (5 nM; MedChemExpress, catalog no. HY-116387). These selection agents were not present during the experiments.

Two cell line authentication methods were used to verify the identities of the 6 parasite cultures. The first evaluated the chloroquine resistance phenotype of each culture by performing proliferation assays, thereby yielding chloroquine IC_50_s for each parasite line or strain. The results were verified by comparing them to previously published data [9,10,20,21] (see the *P*. *falciparum* proliferation assays section for details). Mycoplasma detection assays were also performed periodically on each of the 6 parasite cultures via PCR with a primer mix that amplifies ribosomal DNA from various mycoplasma strains [115]. No mycoplasma infections were detected in any of the parasite cultures throughout the course of this study.

### *P*. *falciparum* H^+^-efflux assays

Parasite cultures were enriched for trophozoite-stage parasites by magnetic separation and were cultured with uninfected erythrocytes loaded with the membrane-impermeant pH-sensitive fluorescent indicator fluorescein-dextran (10,000 MW; Life Technologies, catalog no. D1821). This dye is taken up into the parasite’s DV as they grow inside the erythrocyte. Following a complete asexual cycle (approximately 48 hours) mature trophozoite-stage parasites were isolated from erythrocytes using 0.05% (w/v) saponin and suspended in a saline solution (125 mM NaCl, 5 mM KCl, 1 mM MgCl_2_, 20 mM glucose, and 25 mM HEPES; pH 7.1) at a density of 1 × 10^7^ cells/mL. The fluorometry experiments were performed as outlined previously [21]. Briefly, the pH of the DV was monitored at 37°C using a Cary Eclipse fluorescence spectrophotometer (Agilent Technologies, California, USA) (excitation wavelengths 490 nm and 450 nm; emission wavelength 520 nm). The drugs were added to the parasite suspension (2.5 μM of either chloroquine, saquinavir, mefloquine, or lumefantrine), and the solution was incubated for 4 minutes (chloroquine, saquinavir, and mefloquine) or 8 minutes (lumefantrine) to allow the drugs to accumulate to a high concentration within the DV. This was followed by the addition of the V-type H^+^-ATPase inhibitor concanamycin A (100 nM; Sigma-Aldrich, catalog no. 27689). Half-times for the rate of DV alkalinization were determined in GraphPad Prism Version 8 by a least squares fit of the equation F = F_0_ + F_max_/[1 + (t/t_1/2_)^*c*^] where F is the fluorescence ratio, F_0_ is the initial fluorescence ratio (averaged over 20 seconds immediately prior to opening the chamber of the fluorometer and adding concanamycin A), t is time, t_1/2_ is the half-time for DV alkalinization, F_max_ is the maximal change in fluorescence ratio, and *c* is a fitted constant [20]. The rate of PfCRT-mediated DV alkalinization was calculated by subtracting the rate of DV alkalinization of the solvent control from that of each treatment within each parasite line. In all cases, 4 independent experiments were performed on different days.

### *P*. *falciparum* proliferation assays

The parasite proliferation assays were performed in clear 96-well plates using a fluorescent DNA-intercalating dye as described previously [116,117]. Briefly, synchronous ring-stage parasite cultures (approximately 1% hematocrit and 1% parasitemia) were incubated with increasing concentrations of quinine or chloroquine for 72 hours at 37°C and under reduced O_2_ conditions. The assay was terminated by freezing and thawing the samples, after which 100 μL from each well was transferred to another 96-well plate. This was followed by the addition of 100 μL of SYBR Safe DNA Gel Stain (0.2 μL/mL; Molecular Probes, catalog no. S33102) in a lysis buffer (20 mM Tris-HCl, 5 mM EDTA, 0.008% (w/v) saponin, and 0.08% (v/v) Triton X-100; pH 7.5) to each well. Fluorescence was measured using a Tecan Infinite M1000 PRO microplate reader (excitation wavelength 490 nm; emission wavelength 520 nm). For each 96-well plate, the fluorescence values for the wells containing the highest concentration of the compound were averaged, and this value was then subtracted from the fluorescence values obtained for each well. The level of parasite proliferation in the presence of each compound concentration was expressed as a percentage of the proliferation measured in the absence of the compound. The IC_50_s were determined in GraphPad Prism Version 8 by a least squares fit of the equation y = *a*/[1 + ([compound]/IC_50_)^*c*^] to the data, where y is the percent parasite proliferation, *a* is the maximum change in the percent parasite proliferation, and *c* is a fitted constant. In all cases, 4 independent experiments were performed (on different days), and within each experiment, measurements were averaged from 3 replicates.

### Construction of the MDR1 alignment and generation of the PfMDR1 homology model

Proteins belonging to the MDR1 family were retrieved from the NCBI database using a Basic Local Alignment Search Tool (BLAST) search with PfMDR1 (PF3D7_0523000) as the query sequence. A representative selection of 61 of these proteins, including those for which structures have been determined, were aligned using the ClustalW program [118], and the resulting alignment was manually edited in MacVector Version 12.7.5. Generating an alignment that includes a large number of related proteins is likely to enhance confidence in the alignment of PfMDR1 to a template structure compared with using a simple pairwise alignment. Using this large multiple sequence alignment, we identified 2 proteins related to PfMDR1 with known structures and significant sequence identity: *C*. *elegans* P-gp in the inward-open state [68] (PDB accession code 4F4C, 32.6% sequence identity) and human P-gp in the outward-open state [69] (PDB accession code 6C0V, 33.2% sequence identity). Using the automodel protocol of MODELLER Version 9.17 [119], 100 models of PfMDR1 were built from each template, and the best model was chosen according to the lowest discrete optimized protein energy (DOPE) [120] value calculated by MODELLER Version 9.17. Molecular figures were made using the Visual Molecular Dynamics (VMD) modeling program [121].

Molecular dynamics simulations of quinine in the binding pocket of the inward open PfMDR1 homology model were conducted using the NAMD Version 2.13 simulation code [122]. First, CGENFF force field [123] parameters for quinine were generated using the automated ParamChem CGENFF tool [124,125]. Seven independent simulations were conducted, with random starting positions and orientations of quinine within the large aqueous cavity of PfMDR1. Within the structure of PfMDR1, atoms further than 17 Å from the center of the binding pocket (defined by the midpoint of the backbones of L327 and F1072 that are on opposing sides of the cavity) were harmonically restrained while atoms within this range were not restrained. Protein restraints were decreased from 1 kcal/mol/Å^2^ to 0.001 kcal/mol/Å^2^ over 8 steps (values were 1, 0.5, 0.2, 0.1, 0.05, 0.02, 0.01, and 0.001) with the system energy minimized for 100 steps before 100 ps of dynamics at each step. Once this equilibration phase was complete, simulations were continued for 30 ns with the final harmonic restraint on the protein maintained. The central cavity of PfMDR1 was solvated with TIP3P water molecules that were restrained to stay within a sphere (radius of 20 Å) at the center of the binding pocket, using a spherical harmonic boundary potential applied to the water molecule’s oxygen atoms with a force constant of 20 kcal/mol/Å^2^. The CHARMM36 force field with CMAP correction [126] was used for the protein, and the simulations were performed at a temperature of 300 K using a Langevin thermostat. All bonds to hydrogen were kept fixed, allowing the use of 2 fs timesteps.

### Correlations between in vitro drug resistance indices and the rate of drug transport via PfMDR1

IC_50_s for lumefantrine, mefloquine, chloroquine, and quinine were collated from the relevant literature (see S3 Table for references). The inclusion criteria that were applied to previously published studies were: (1) that an IC_50_ for one or more of the parasite strains of interest (i.e., HB3, Dd2, K1, GB4, or 7G8) be reported, and (2) that the IC_50_ for the 3D7 control strain be performed pairwise. For lumefantrine, mefloquine, and quinine, 4 to 10 studies fitted these criteria and were thus incorporated into the analyses. Several of the studies that measured chloroquine IC_50_s did not include pairwise measurements with the 3D7; instead, pairwise measurements with either HB3 or GC03 parasites were made (both of these strains express PfCRT^3D7^ and PfMDR1^NFSDD^, and their chloroquine IC_50_s are typically very similar to 3D7 in pairwise studies). Since these studies measured chloroquine IC_50_s for infrequently investigated parasite strains such as GB4, it was important to include them in our analyses. Therefore, the inclusion criteria for the chloroquine studies were altered to include those that had measured chloroquine IC_50_s in either HB3 or GC03 parasites as the control strains, regardless of whether the 3D7 strain was included. The in vitro resistance indices for each study were calculated by dividing the IC_50_ of each strain by the IC_50_ of the control strain (3D7 for lumefantrine, mefloquine, and quinine; HB3 or GC03 for chloroquine). The indices across multiple studies were then averaged and the standard error of the mean calculated (or the range/2 for resistance indices that were calculated from only 2 studies). For each drug, the resistance indices were plotted against the experimentally determined transport activities of the PfMDR1 isoforms in *Xenopus* oocytes (S1 Data).

### Quantification and statistical analyses

The statistical tests, data collection, and analyses were performed using ImageJ Version 1.8.0, GraphPad Prism Version 8, Leica Application Suite Advanced Fluorescence software Version 3.6, Leica Application Suite software Version 4.12, Image Lab version 6.0.1 software (Bio-Rad), MODELLER Version 9.17, VMD, NAMD Version 2.13, and MarvinSketch software Version 18.10. Statistical comparisons were made using 1-way ANOVAs in conjunction with Tukey multiple comparisons test. All errors cited in the text and shown in the figures represent the SEM or the range/2. Where not shown, error bars fall within the symbols. Significance was defined as *P* < 0.05. The key statistical details for each dataset are included in the figure legends.

## Supporting information

S1 Fig PfNT1 transports [^3^H]hypoxanthine when expressed in *Xenopus* oocytes. Oocytes expressing PfNT1 were used as a negative control in the experiments measuring [^3^H]drug efflux from oocytes expressing human P-gp or PfMDR1 and in the experiments measuring [^3^H]lumefantrine or [^3^H]VDPVNF efflux from oocytes expressing PfCRT. This control demonstrated that the heterologous expression of a transporter in *Xenopus* oocytes does not affect the ability of the oocyte membrane to reseal following the microinjection of a [^3^H]drug or [^3^H]VDPVNF. To confirm that PfNT1 was expressed and functional at the oocyte plasma membrane, the uptake of hypoxanthine (a known substrate of PfNT1) was measured in oocytes expressing the transporter, as well as in nonexpressing (ne) oocytes. The low level of [^3^H]hypoxanthine accumulation in ne is due to the simple diffusion of the unprotonated species. The data are the mean of *n* = 4 independent experiments, each yielding similar results and overlaid as individual data points, and the error is the SEM. The asterisks denote a significant difference from ne; ***P < 0.001 (1-way ANOVA). The data underlying this figure is supplied in S3 Data. ne, nonexpressing oocytes; PfCRT, *Plasmodium falciparum* chloroquine resistance transporter; PfNT1, *Plasmodium falciparum* nucleoside transporter 1; PfMDR1, *Plasmodium falciparum* multidrug resistance protein 1; P-gp, P-glycoprotein.
(TIF)

S2 Fig Known inhibitors of human P-gp inhibit the transport of lumefantrine via PfMDR1. **(a)** Schematic showing the *cis*-inhibition of [^3^H]drug transport via human P-gp or PfMDR1 by known P-gp inhibitors in the *Xenopus* oocyte system. **(b)** The IC_50_ values of nicardipine, PSC833, vanadate, and verapamil against [^3^H]vinblastine transport via human P-gp and [^3^H]lumefantrine transport via PfMDR1. The data are the mean of *n* = 4 independent experiments, and the error is the SEM. The data underlying this figure is supplied in S3 Data. PfMDR1, *Plasmodium falciparum* multidrug resistance protein 1; P-gp, P-glycoprotein.
(TIF)

S3 Fig Predicted topologies of PfMDR1 and PfCRT. **(a)** PfMDR1 consists of 1,419 amino acid residues that are arranged into 12 TMDs and 2 NBDs. The length of the loops shown surrounding the NBDs are approximate. **(b)** PfCRT consists of 424 amino acid residues that are arranged into 10 TMDs. All loops are representative of the true loop length. The positions of the mutations in the isoforms of PfMDR1 and PfCRT used in this study are indicated by pink circles and the box attached to each polymorphic residue lists the (non-wild type) amino acid(s) that occur at that position. NBD, nucleotide-binding domain; PfCRT, *Plasmodium falciparum* chloroquine resistance transporter; PfMDR1, *Plasmodium falciparum* multidrug resistance protein 1; TMD, transmembrane domain.
(TIF)

S4 Fig PfMDR1 is expressed at the oocyte surface and is orientated with its termini in the cytosol. **(a)** Immunofluorescence microscopy images confirmed that the expression of each of the 3xHA-tagged PfMDR1 isoforms resulted in a fluorescent band external to the pigment layer, indicating that the proteins were expressed in the oocyte plasma membrane. The band was not present in ne. Oocytes expressing a HA-tagged version of the *Apis mellifera* dopamine transporter (HA_EL2_-AmDAT) at the plasma membrane serve as a positive control [113]. The length of the scale bar is 50 μm. The images are representative of at least 2 independent experiments (performed using oocytes from different frogs), within which images were obtained from a minimum of 3 oocytes per oocyte type. **(b)** Immunofluorescence microscopy was used to determine the orientation of PfMDR1 in the plasma membrane of live oocytes using an anti-HA antibody and a fluorescent secondary antibody. The anti-HA antibody will bind to extracellular HA-tags and cannot access those that are intracellular. Hence, only proteins with extracellular HA-tags will be detected by the anti-HA antibody. The fluorescent signal in oocytes expressing HA_EL2_-AmDAT is at its strongest at the periphery of the oocyte, indicating that AmDAT is orientated in the oocyte membrane such that the HA-tagged loop is extracellular. There was no fluorescent signal in the ne (the negative control) or in oocytes expressing 3xHA-PfMDR1^NYSND^ or PfMDR1^NYSND^-3xHA (see S1 Text). This indicates that the amino terminus and carboxyl terminus of PfMDR1 are located in the cytosol of the oocyte. The length of the scale bar is 1 mm. The images are representative of 3 independent experiments, within which images were obtained from a minimum of 3 oocytes per oocyte type. HA, hemagglutinin; ne, nonexpressing oocytes; PfMDR1, *Plasmodium falciparum* multidrug resistance protein 1.
(TIFF)

S5 Fig Measurement of protein levels and [^3^H]lumefantrine transport activities of HA-tagged PfMDR1 isoforms in *Xenopus* oocytes. **(a)** Analysis of the PfMDR1 levels in different dilutions of a membrane protein preparation from 3xHA-PfMDR1^NYSND^-expressing oocytes revealed a sigmoidal relationship between the relative quantity of membrane protein and the intensity of the corresponding PfMDR1 band (r^2^ = 0.99). **(b)** A sigmoidal relationship was observed between the intensity of the 3xHA-PfMDR1^NYSND^ protein band and the amount of 3xHA-PfMDR1^NYSND^ cRNA microinjected into oocytes (r^2^ = 0.99). The relationship is approximately linear between 2.5 and 10 ng of cRNA. **(c)** A sigmoidal relationship was observed between the level of [^3^H]lumefantrine transport and the quantity of 3xHA-PfMDR1^NYSND^ cRNA microinjected into the oocyte (r^2^ = 0.99). This relationship is linear between 1 and 10 ng of cRNA, with [^3^H]lumefantrine transport saturating above this range. **(d)** Combining the plots in panels **b** and **c** revealed that in oocytes microinjected with 2.5 to 10 ng of 3xHA-PfMDR1^3D7^ cRNA, the intensity of the 3xHA-PfMDR1^NYSND^ protein band strongly correlates with the level of 3xHA-PfMDR1^NYSND^-mediated [^3^H]lumefantrine transport (r^2^ = 0.9904). **(e)** Semiquantification of PfMDR1 protein levels in the membranes of oocytes expressing either 3xHA-PfMDR1^NYSND^, PfMDR1^NYSND^-2xHA, or PfMDR1^NYSND^-3xHA revealed that, relative to 3xHA-PfMDR1^NYSND^, the addition of HA-tags to the carboxyl terminus of the protein significantly reduced PfMDR1 levels. **(f)** Oocytes expressing PfMDR1^NYSND^-2xHA or PfMDR1^NYSND^-3xHA also displayed significantly lower levels of [^3^H]lumefantrine transport relative to those expressing 3xHA-PfMDR1^NYSND^. **(g)** Densitometric analyses of total protein indicated that there were no significant differences between sample lanes in the western blot experiments that gave rise to Fig 1G. In each experiment, the protein on the nitrocellulose membrane was stained with Ponceau S and the intensity between 70 and 180 kDa was measured within each lane. **(h)** 3xHA-PfMDR1^NYSND^ and 3xHA-PfMDR1^NFCDY^ had slightly reduced lumefantrine transport activities relative to the respective untagged proteins. The data are the mean of 4 to 9 independent experiments (each yielding similar results and overlaid as individual data points in panels **e**–**h**), and the error is the SEM. Where not visible, the error bars fall within the symbols. The asterisks denote a significant difference from the 3xHA-PfMDR1^NYSND^ control or the PfMDR1^NYSND^ control; *P < 0.05, ***P < 0.001, ns, not significant (1-way ANOVA). The data underlying this figure is supplied in S3 Data. ne, nonexpressing oocytes; PfMDR1, *Plasmodium falciparum* multidrug resistance protein 1.
(TIFF)

S6 Fig Interaction between quinine and the 1042D residue in PfMDR1^NFSDD^. The movement of the quinine molecule during the 7 molecular dynamics simulations is indicated by the distance between the protonated cyclic amine ring of quinine and the carboxylate oxygen atom on the 1042D side chain over the duration of the simulation. Seven simulations were performed, each represented by a different colored line. Quinine moves close to D1042 in 4 of the 7 simulations (denoted by the yellow, blue, navy, and green lines), forming stable, long-lasting hydrogen bonds in 2 of these simulations (denoted by the navy and green lines). PfMDR1, *Plasmodium falciparum* multidrug resistance protein 1.
(TIF)

S7 Fig Transport of lumefantrine and mefloquine via PfCRT in *Xenopus* oocytes. **(a, c)** The transport of [^3^H]lumefantrine (a) and [^3^H]mefloquine (c) via PfCRT^Dd2^ was approximately linear with time for at least 2 hours. **(b, d)** The transport of [^3^H]lumefantrine (b) and [^3^H]mefloquine (d) via PfCRT^Dd2^ and PfCRT^7G8^ was reduced by known inhibitors of the transporter (verapamil, chlorpheniramine, and saquinavir) and was unaffected by histidine and LH (metabolites that do not interact with PfCRT). The data are the mean of *n* = 4 independent experiments, each yielding similar results and overlaid as individual data points in panels **b** and **d**, and the error is the SEM. Where not visible, the error bars fall within the symbols. The asterisks denote a significant difference from the PfCRT^Dd2^ control (red asterisks) or the PfCRT^7G8^ control (orange asterisks): **P < 0.01, ***P < 0.001 (1-way ANOVA). The data underlying this figure is supplied in S3 Data. PfCRT, *Plasmodium falciparum* chloroquine resistance transporter.
(TIF)

S8 Fig Structures of a subset of compounds used in this study. The pK_a_ values for the protonatable nitrogen(s) within each compound are indicated. The structures and pK_a_ values were collated from the literature [127] or generated in the MarvinSketch software (ChemAxon). pK_a_, the negative logarithm to the base 10 of the acid dissociation constant.
(TIF)

S9 Fig Direction of drug transport via PfMDR1 and PfCRT in situ versus in *Xenopus* oocytes. PfMDR1 orientates in the DV membrane such that its amino terminus and carboxyl terminus, as well as its NBDs, are located in the parasite cytosol [36]. It was expected that PfMDR1 would adopt the same orientation in the oocyte plasma membrane (i.e., with its termini and NBDs extending into the oocyte cytosol) due to the “positive inside rule” [128,129]. This orientation was confirmed using immunofluorescence microscopy and live oocytes expressing HA-tagged versions of PfMDR1 (S4 Fig). PfCRT also orientates in the DV membrane with its amino terminus and carboxyl terminus in the parasite cytosol [130] and is predicted, on the basis of the “positive inside rule,” to adopt the same orientation in the oocyte plasma membrane (i.e., with its amino terminus and carboxyl terminus in the oocyte cytosol). **(a)** In the malaria parasite, PfMDR1 mediates the transport of drugs from the parasite cytosol (pH approximately 7.3) into the acidic environment of the DV (pH 5.0 to 5.5), where the weak base drugs will become protonated [94]. In the *Xenopus* oocyte system, the drug under study is microinjected into the oocyte cytosol (pH approximately 7.2) and is transported via PfMDR1 into the extracellular solution (pH 5.5). Thus, in both cases, the direction of PfMDR1-mediated drug transport is from the cell cytosol into the acidic DV lumen or the acidic extracellular solution. **(b)** Weak base drugs also accumulate in the parasite DV via simple diffusion of the neutral species, which becomes protonated upon entering the acidic lumen of this organelle. In parasites that express chloroquine resistance-conferring isoforms of PfCRT, the transporter mediates the transport of the protonated drug from the DV into the parasite cytosol. In the *Xenopus* oocyte expression system, the drug under study is typically added to the acidic extracellular solution and mutant isoforms of PfCRT transport the protonated drug into the oocyte cytosol. Therefore, in both scenarios, the direction of PfCRT-mediated drug transport is from the acidic DV lumen/acidic extracellular solution into the cell cytosol. That said, in the case of lumefantrine, the drug was microinjected into the oocyte and its transport from the cytosol into the extracellular solution was measured. This modified protocol was used because lumefantrine transport via PfCRT appears to be reliant on the (as yet unidentified) second cosubstrate of the transporter [10]. The endogenous substrates of PfCRT, such as the hemoglobin-derived hexapeptide VDPVNF, are translocated with H^+^ and a second cosubstrate that is naturally present within the oocyte cytosol [10]. It should be noted, however, that PfCRT is a carrier [6,10,131], which means it can mediate the transport of a substrate in either direction across the membrane (assuming the necessary cosubstrates are present), with the net direction of transport determined by the prevailing electrochemical gradients of the substrates. In the parasite DV, these gradients will result in lumefantrine being cotransported with H^+^ (and the unidentified second cosubstrate) from the DV lumen into the parasite cytosol. DV, digestive vacuole; NBD, nucleotide-binding domain; PfCRT, *Plasmodium falciparum* chloroquine resistance transporter; PfMDR1, *Plasmodium falciparum* multidrug resistance protein 1.
(TIF)

S10 Fig Iron chelator IV does not affect drug transport via PfMDR1. Measurements of [^3^H]dihydroartemisinin transport via PfMDR1 were made in the presence of the iron chelator IV to bind any free iron present in the oocyte. Free iron could activate dihydroartemisinin, thereby damaging the oocytes and interfering with measurements of transport. The effect of iron chelator IV (estimated intracellular concentration of 1 mM) on [^3^H]lumefantrine transport was measured in ne and oocytes expressing either PfNT1, PfMDR1^NYSND^, or PfMDR1^NFCDY^. The data are the mean of *n* = 4 independent experiments (each yielding similar results and overlaid as individual data points), and the error is the SEM. Where not visible, the error bars fall within the symbols. Statistical analyses were performed relative to the PfMDR1^NYSND^ control (blue) or the PfMDR1^NFCDY^ (dark pink) control; ns, not significant (1-way ANOVA). The data underlying this figure is supplied in S3 Data. ne, nonexpressing oocytes; PfMDR1, *Plasmodium falciparum* multidrug resistance protein 1; PfNT1, *Plasmodium falciparum* nucleoside transporter 1.
(TIF)

S1 Table Amino acid mutations in the PfMDR1 isoforms included for study. PfMDR1, *Plasmodium falciparum* multidrug resistance protein 1.
(PDF)

S2 Table Amino acid mutations in the PfCRT isoforms included for study. PfCRT, *Plasmodium falciparum* chloroquine resistance transporter.
(PDF)

S3 Table In vitro resistance indices of *P*. *falciparum* strains for lumefantrine, mefloquine, chloroquine, and quinine. (PDF)

S4 Table Generation of PfMDR1 coding sequences for expression in *Xenopus* oocytes. PfMDR1, *Plasmodium falciparum* multidrug resistance protein 1.
(PDF)

S1 Text Effects of amino-terminal or carboxyl-terminal epitope tags on the expression and function of PfMDR1 in *Xenopus* oocytes. PfMDR1, *Plasmodium falciparum* multidrug resistance protein 1.
(PDF)

S2 Text Direct measurement of the transport of fluorescent compounds via PfMDR1. PfMDR1, *Plasmodium falciparum* multidrug resistance protein 1.
(PDF)

S3 Text Roles of PfMDR1 in modulating the parasite’s response to a range of antimalarial drugs. PfMDR1, *Plasmodium falciparum* multidrug resistance protein 1.
(PDF)

S4 Text Quinine resistance in the malaria parasite is a multifactorial phenomenon. (PDF)

S1 File Alignment of MDR1 proteins used to generate the PfMDR1 homology model. PfMDR1, *Plasmodium falciparum* multidrug resistance protein 1.
(PDF)

S1 Data Rates of PfMDR1-mediated drug transport. PfMDR1, *Plasmodium falciparum* multidrug resistance protein 1.
(XLSX)

S2 Data Protonated drug proportions. (XLSX)

S3 Data Source data file containing the underlying data presented in Figs 1–6, Table 1, and S1, S2, S5, S7, and S10 Figs. (XLSX)

## Abbreviations:

ABC: ATP-binding cassette
BLAST: Basic Local Alignment Search Tool
app Km: apparent Michaelis constant
app Vmax: apparent maximal velocity
DOPE: discrete optimized protein energy
DV: digestive vacuole
HA: hemagglutinin
NBD: nucleotide-binding domain
ne: nonexpressing oocytes
PfCRT: *Plasmodium falciparum* chloroquine resistance transporter
PfMDR1: *Plasmodium falciparum* multidrug resistance protein 1
PfNT1: *Plasmodium falciparum* nucleoside transporter 1
P-gp: P-glycoprotein
VMD: Visual Molecular Dynamics

## References

[1] Martin RE. The transportome of the malaria parasite. Biol Rev. 2020;95:305–32. doi: 10.1111/brv.12565 31701663

[2] Sisowath C, Petersen I, Veiga MI, Martensson A, Premji Z, Bjorkman A, et al. *In vivo* selection of *Plasmodium falciparum* parasites carrying the chloroquine-susceptible *pfcrt* K76 allele after treatment with artemether-lumefantrine in Africa. J Infect Dis. 2009;199:750–7. doi: 10.1086/596738 19210165 PMC2718568

[3] Otienoburu SD, Maiga-Ascofare O, Schramm B, Jullien V, Jones JJ, Zolia YM, et al. Selection of *Plasmodium falciparum pfcrt* and *pfmdr1* polymorphisms after treatment with artesunate-amodiaquine fixed dose combination or artemether-lumefantrine in Liberia. Malar J. 2016;15:452. doi: 10.1186/s12936-016-1503-3 27596849 PMC5011943

[4] Sisowath C, Strömberg J, Mårtensson A, Msellem M, Obondo C, Björkman A, et al. *In vivo* selection of *Plasmodium falciparum pfmdr1* 86N coding alleles by artemether-lumefantrine (Coartem). J Infect Dis. 2005;191:1014–7. doi: 10.1086/427997 15717281

[5] Zhang M, Wang C, Otto TD, Oberstaller J, Liao X, Adapa SR, et al. Uncovering the essential genes of the human malaria parasite *Plasmodium falciparum* by saturation mutagenesis. Science (New York, NY). 2018;360: eaap7847. doi: 10.1126/science.aap7847 29724925 PMC6360947

[6] Martin RE, Marchetti RV, Cowan AI, Howitt SM, Broer S, Kirk K. Chloroquine transport via the malaria parasite’s chloroquine resistance transporter. Science (New York, NY). 2009;325:1680–2. doi: 10.1126/science.1175667 19779197

[7] Summers RL, Dave A, Dolstra TJ, Bellanca S, Marchetti RV, Nash MN, et al. Diverse mutational pathways converge on saturable chloroquine transport via the malaria parasite’s chloroquine resistance transporter. Proc Natl Acad Sci U S A. 2014;111:E1759–67. doi: 10.1073/pnas.1322965111 24728833 PMC4035986

[8] van Schalkwyk DA, Nash MN, Shafik SH, Summers RL, Lehane AM, Smith PJ, et al. Verapamil-sensitive transport of quinacrine and methylene blue via the *Plasmodium falciparum* chloroquine resistance transporter reduces the parasite’s susceptibility to these tricyclic drugs. J Infect Dis. 2016;213:800–10. doi: 10.1093/infdis/jiv509 26503982

[9] Richards SN, Nash MN, Baker ES, Webster MW, Lehane AM, Shafik SH, et al. Molecular mechanisms for drug hypersensitivity induced by the malaria parasite’s chloroquine resistance transporter. PLoS Pathog. 2016;12:e1005725. doi: 10.1371/journal.ppat.1005725 27441371 PMC4956231

[10] Shafik SH, Cobbold SA, Barkat K, Richards SN, Lancaster NS, Llinás M, et al. The natural function of the malaria parasite’s chloroquine resistance transporter. Nat Commun. 2020;11:3922. doi: 10.1038/s41467-020-17781-6 32764664 PMC7413254

[11] Papakrivos J, Sa JM, Wellems TE. Functional characterization of the *Plasmodium falciparum* chloroquine-resistance transporter (PfCRT) in transformed *Dictyostelium discoideum* vesicles. PLoS ONE. 2012;7:e39569. doi: 10.1371/journal.pone.0039569 22724026 PMC3378554

[12] Callaghan PS, Siriwardana A, Hassett MR, Roepe PD. *Plasmodium falciparum* chloroquine resistance transporter (PfCRT) isoforms PH1 and PH2 perturb vacuolar physiology. Malar J. 2016;15:186. doi: 10.1186/s12936-016-1238-1 27036417 PMC4815217

[13] Callaghan PS, Hassett MR, Roepe PD. Functional comparison of 45 naturally occurring isoforms of the *Plasmodium falciparum* chloroquine resistance transporter (PfCRT). Biochemistry. 2015;54:5083–94. doi: 10.1021/acs.biochem.5b00412 26208441 PMC5070608

[14] Baro NK, Pooput C, Roepe PD. Analysis of chloroquine resistance transporter (CRT) isoforms and orthologues in *S*. *cerevisiae* yeast. Biochemistry. 2011;50:6701–10. doi: 10.1021/bi200922g 21744797 PMC3155940

[15] Summers RL, Nash MN, Martin RE. Know your enemy: understanding the role of PfCRT in drug resistance could lead to new antimalarial tactics. Cell Mol Life Sci. 2012;69:1967–95. doi: 10.1007/s00018-011-0906-0 22286067 PMC11115045

[16] Fidock DA, Nomura T, Talley AK, Cooper RA, Dzekunov SM, Ferdig MT, et al. Mutations in the *P*. *falciparum* digestive vacuole transmembrane protein PfCRT and evidence for their role in chloroquine resistance. Mol Cell. 2000;6:861–71. doi: 10.1016/s1097-2765(05)00077-8 11090624 PMC2944663

[17] Sidhu AB, Verdier-Pinard D, Fidock DA. Chloroquine resistance in *Plasmodium falciparum* malaria parasites conferred by *pfcrt* mutations. Science (New York, NY). 2002;298:210–3. doi: 10.1126/science.1074045 12364805 PMC2954758

[18] Bellanca S, Summers RL, Meyrath M, Dave A, Nash MN, Dittmer M, et al. Multiple drugs compete for transport via the *Plasmodium falciparum* chloroquine resistance transporter at distinct but interdependent sites. J Biol Chem. 2014;289:36336–51. doi: 10.1074/jbc.M114.614206 25378409 PMC4276893

[19] Kim J, Tan YZ, Wicht KJ, Erramilli SK, Dhingra SK, Okombo J, et al. Structure and drug resistance of the *Plasmodium falciparum* transporter PfCRT. Nature. 2019;576:315–20. doi: 10.1038/s41586-019-1795-x 31776516 PMC6911266

[20] Lehane AM, Hayward R, Saliba KJ, Kirk K. A verapamil-sensitive chloroquine-associated H^+^ leak from the digestive vacuole in chloroquine-resistant malaria parasites. J Cell Sci. 2008;121:1624–32. doi: 10.1242/jcs.016758 18445688

[21] Lehane AM, Kirk K. Chloroquine resistance-conferring mutations in *pfcrt* give rise to a chloroquine-associated H^+^ leak from the malaria parasite’s digestive vacuole. Antimicrob Agents Chemother. 2008;52:4374–80. doi: 10.1128/AAC.00666-08 18852275 PMC2592892

[22] Lehane AM, Kirk K. Efflux of a range of antimalarial drugs and ’chloroquine resistance reversers’ from the digestive vacuole in malaria parasites with mutant PfCRT. Mol Microbiol. 2010;77:1039–51. doi: 10.1111/j.1365-2958.2010.07272.x 20598081

[23] Duraisingh MT, Jones P, Sambou I, von Seidlein L, Pinder M, Warhurst DC. The tyrosine-86 allele of the *pfmdr1* gene of *Plasmodium falciparum* is associated with increased sensitivity to the anti-malarials mefloquine and artemisinin. Mol Biochem Parasitol. 2000;108:13–23. doi: 10.1016/s0166-6851(00)00201-2 10802315

[24] Duraisingh MT, Roper C, Walliker D, Warhurst DC. Increased sensitivity to the antimalarials mefloquine and artemisinin is conferred by mutations in the *pfmdr1* gene of *Plasmodium falciparum*. Mol Microbiol. 2000;36:955–61. doi: 10.1046/j.1365-2958.2000.01914.x 10844681

[25] Eyase FL, Akala HM, Ingasia L, Cheruiyot A, Omondi A, Okudo C, et al. The role of *Pfmdr1* and *Pfcrt* in changing chloroquine, amodiaquine, mefloquine and lumefantrine susceptibility in western-Kenya *P*. *falciparum* samples during 2008–2011. PLoS ONE. 2013;8:e64299. doi: 10.1371/journal.pone.0064299 23675533 PMC3652850

[26] Foote SJ, Kyle DE, Martin RK, Oduola AM, Forsyth K, Kemp DJ, et al. Several alleles of the multidrug-resistance gene are closely linked to chloroquine resistance in *Plasmodium falciparum*. Nature. 1990;345:255–8. doi: 10.1038/345255a0 2185424

[27] Nsobya SL, Kiggundu M, Nanyunja S, Joloba M, Greenhouse B, Rosenthal PJ. *In vitro* sensitivities of *Plasmodium falciparum* to different antimalarial drugs in Uganda. Antimicrob Agents Chemother. 2010;54:1200–6. doi: 10.1128/AAC.01412-09 20065051 PMC2825959

[28] Wurtz N, Fall B, Pascual A, Fall M, Baret E, Camara C, et al. Role of *Pfmdr1* in *in vitro Plasmodium falciparum* susceptibility to chloroquine, quinine, monodesethylamodiaquine, mefloquine, lumefantrine, and dihydroartemisinin. Antimicrob Agents Chemother. 2014;58:7032–40. doi: 10.1128/AAC.03494-14 25199781 PMC4249527

[29] Sidhu AB, Valderramos SG, Fidock DA. *pfmdr1* mutations contribute to quinine resistance and enhance mefloquine and artemisinin sensitivity in *Plasmodium falciparum*. Mol Microbiol. 2005;57:913–26. doi: 10.1111/j.1365-2958.2005.04729.x 16091034

[30] Cowman AF, Galatis D, Thompson JK. Selection for mefloquine resistance in *Plasmodium falciparum* is linked to amplification of the *pfmdr1* gene and cross-resistance to halofantrine and quinine. Proc Natl Acad Sci U S A. 1994;91:1143–7. doi: 10.1073/pnas.91.3.1143 8302844 PMC521470

[31] Peel SA, Bright P, Yount B, Handy J, Baric RS. A strong association between mefloquine and halofantrine resistance and amplification, overexpression, and mutation in the P-glycoprotein gene homolog (*pfmdr*) of *Plasmodium falciparum in vitro*. Am J Trop Med Hyg. 1994;51:648–58. doi: 10.4269/ajtmh.1994.51.648 7985758

[32] Price RN, Uhlemann AC, Brockman A, McGready R, Ashley E, Phaipun L, et al. Mefloquine resistance in *Plasmodium falciparum* and increased *pfmdr1* gene copy number. Lancet (London, England). 2004;364:438–47. doi: 10.1016/S0140-6736(04)16767-6 15288742 PMC4337987

[33] Sondo P, Derra K, Diallo Nakanabo S, Tarnagda Z, Kazienga A, Zampa O, et al. Artesunate-amodiaquine and artemether-lumefantrine therapies and selection of *Pfcrt* and *Pfmdr1* alleles in Nanoro, Burkina Faso. PLoS ONE. 2016;11:e0151565. doi: 10.1371/journal.pone.0151565 27031231 PMC4816516

[34] Windle ST, Lane KD, Gadalla NB, Liu A, Mu J, Caleon RL, et al. Evidence for linkage of *pfmdr1*, *pfcrt*, and *pfk13* polymorphisms to lumefantrine and mefloquine susceptibilities in a *Plasmodium falciparum* cross. Int J Parasitol. 2020;14:208–17. 10.1016/j.ijpddr.2020.10.009 PMC7677662 33197753

[35] Yeka A, Kigozi R, Conrad MD, Lugemwa M, Okui P, Katureebe C, et al. Artesunate/amodiaquine versus artemether/lumefantrine for the treatment of uncomplicated malaria in Uganda: A randomized trial. J Infect Dis. 2016;213:1134–42. doi: 10.1093/infdis/jiv551 26597254 PMC4836737

[36] Karcz SR, Galatis D, Cowman AF. Nucleotide binding properties of a P-glycoprotein homologue from *Plasmodium falciparum*. Mol Biochem Parasitol. 1993;58:269–76. doi: 10.1016/0166-6851(93)90048-3 8097560

[37] Sanchez CP, Rotmann A, Stein WD, Lanzer M. Polymorphisms within PfMDR1 alter the substrate specificity for anti-malarial drugs in *Plasmodium falciparum*. Mol Microbiol. 2008;70:786–98. doi: 10.1111/j.1365-2958.2008.06413.x 18713316

[38] van Es HH, Karcz S, Chu F, Cowman AF, Vidal S, Gros P, et al. Expression of the plasmodial *pfmdr1* gene in mammalian cells is associated with increased susceptibility to chloroquine. Mol Cell Biol. 1994;14:2419–28. doi: 10.1128/mcb.14.4.2419-2428.1994 7511206 PMC358609

[39] Amoah LE, Lekostaj JK, Roepe PD. Heterologous expression and ATPase activity of mutant versus wild type PfMDR1 protein. Biochemistry. 2007;46:6060–73. doi: 10.1021/bi7002026 17469853

[40] Pleeter P, Lekostaj JK, Roepe PD. Purified *Plasmodium falciparum* multi-drug resistance protein (PfMDR 1) binds a high affinity chloroquine analogue. Mol Biochem Parasitol. 2010;173:158–61. doi: 10.1016/j.molbiopara.2010.05.012 20546803 PMC2906614

[41] Volkman SK, Cowman AF, Wirth DF. Functional complementation of the *ste6* gene of *Saccharomyces cerevisiae* with the *pfmdr1* gene of *Plasmodium falciparum*. Proc Natl Acad Sci U S A. 1995;92:8921–5. doi: 10.1073/pnas.92.19.8921 7568044 PMC41079

[42] Ruetz S, Delling U, Brault M, Schurr E, Gros P. The *pfmdr1* gene of *Plasmodium falciparum* confers cellular resistance to antimalarial drugs in yeast cells. Proc Natl Acad Sci U S A. 1996;93:9942–7. doi: 10.1073/pnas.93.18.9942 8790436 PMC38534

[43] Shalinsky DR, Jekunen AP, Alcaraz JE, Christen RD, Kim S, Khatibi S, et al. Regulation of initial vinblastine influx by P-glycoprotein. Br J Cancer. 1993;67:30–6. doi: 10.1038/bjc.1993.6 8094005 PMC1968208

[44] Eytan GD, Regev R, Oren G, Hurwitz CD, Assaraf YG. Efficiency of P-glycoprotein-mediated exclusion of rhodamine dyes from multidrug-resistant cells is determined by their passive transmembrane movement rate. Eur J Biochem. 1997;15:104–12. doi: 10.1111/j.1432-1033.1997.00104.x 9310367

[45] Senarathna SM, Page-Sharp M, Crowe A. The interactions of P-glycoprotein with antimalarial drugs, including substrate affinity, inhibition and regulation. PLoS ONE. 2016;11:e0152677. doi: 10.1371/journal.pone.0152677 27045516 PMC4821601

[46] Polli JW, Wring SA, Humphreys JE, Huang L, Morgan JB, Webster LO, et al. Rational use of *in vitro* P-glycoprotein assays in drug discovery. J Pharmacol Exp Ther. 2001;299:620–8. 11602674

[47] Suzuki T, Fukami T, Tomono K. Possible involvement of cationic-drug sensitive transport systems in the blood-to-brain influx and brain-to-blood efflux of amantadine across the blood-brain barrier. Biopharm Drug Dispos. 2015;36:126–37. doi: 10.1002/bdd.1926 25410756

[48] Doan KMM, Humphreys JE, Webster LO, Wring SA, Shampine LJ, Serabjit-Singh CJ, et al. Passive permeability and P-glycoprotein-mediated efflux differentiate central nervous system (CNS) and non-CNS marketed drugs. J Pharmacol Exp Ther. 2002;303:1029–37. doi: 10.1124/jpet.102.039255 12438524

[49] Mwai L, Diriye A, Masseno V, Muriithi S, Feltwell T, Musyoki J, et al. Genome wide adaptations of *Plasmodium falciparum* in response to lumefantrine selective drug pressure. PLoS ONE. 2012;7:e31623. doi: 10.1371/journal.pone.0031623 22384044 PMC3288012

[50] Wong W, Bai XC, Sleebs BE, Triglia T, Brown A, Thompson JK, et al. Mefloquine targets the *Plasmodium falciparum* 80S ribosome to inhibit protein synthesis. Nat Microbiol. 2017;2:17031. doi: 10.1038/nmicrobiol.2017.31 28288098 PMC5439513

[51] Solomonov I, Osipova M, Feldman Y, Baehtz C, Kjaer K, Robinson IK, et al. Crystal nucleation, growth, and morphology of the synthetic malaria pigment β-hematin and the effect thereon by quinoline additives: The malaria pigment as a target of various antimalarial drugs. J Am Chem Soc. 2007;129:2615–27. doi: 10.1021/ja0674183 17290993

[52] Buller R, Peterson ML, Almarsson Ö, Leiserowitz L. Quinoline binding site on malaria pigment crystal: A rational pathway for antimalaria drug design. Cryst Growth Des. 2002;2:553–62.

[53] Bergeron MJ, Boggavarapu R, Meury M, Ucurum Z, Caron L, Isenring P, et al. Frog oocytes to unveil the structure and supramolecular organization of human transport proteins. PLoS ONE. 2011;6:e21901. doi: 10.1371/journal.pone.0021901 21760919 PMC3131388

[54] Safa AR. Photoaffinity labeling of the multidrug-resistance-related P-glycoprotein with photoactive analogs of verapamil. Proc Natl Acad Sci U S A. 1988;85:7187–91. doi: 10.1073/pnas.85.19.7187 2902625 PMC282149

[55] Boesch D, Gavériaux C, Jachez B, Pourtier-Manzanedo A, Bollinger P, Loor F. *In vivo* circumvention of P-glycoprotein-mediated multidrug resistance of tumor cells with SDZ PSC 833. Cancer Res. 1991;51:4226–33. 1678313

[56] Twentyman PR, Bleehen NM. Resistance modification by PSC-833, a novel non-immunosuppressive cyclosporin. Eur J Cancer. 1991;27:1639–42. doi: 10.1016/0277-5379(91)90435-g 1816768

[57] Urbatsch IL, Sankaran B, Weber J, Senior AE. P-glycoprotein is stably inhibited by vanadate-induced trapping of nucleotide at a single catalytic site. J Biol Chem. 1995;270:19383–90. doi: 10.1074/jbc.270.33.19383 7642618

[58] Urbatsch IL, Beaudet L, Carrier I, Gros P. Mutations in either nucleotide-binding site of P-glycoprotein (Mdr3) prevent vanadate trapping of nucleotide at both sites. Biochemistry. 1998;37:4592–602. doi: 10.1021/bi9728001 9521779

[59] Beck WT, Cirtain MC, Glover CJ, Felsted RL, Safa AR. Effects of indole alkaloids on multidrug resistance and labeling of P-glycoprotein by a photoaffinity analog of vinblastine. Biochem Biophys Res Commun. 1988;153:959–66. doi: 10.1016/s0006-291x(88)81321-4 2898941

[60] Carter NS, Ben Mamoun C, Liu W, Silva EO, Landfear SM, Goldberg DE, et al. Isolation and functional characterization of the PfNT1 nucleoside transporter gene from *Plasmodium falciparum*. J Biol Chem. 2000;275:10683–91. doi: 10.1074/jbc.275.14.10683 10744765

[61] Parker MD, Hyde RJ, Yao SY, McRobert L, Cass CE, Young JD, et al. Identification of a nucleoside/nucleobase transporter from *Plasmodium falciparum*, a novel target for anti-malarial chemotherapy. Biochem J. 2000;349:67–75. doi: 10.1042/0264-6021:3490067 10861212 PMC1221121

[62] Urbatsch IL, Gimi K, Wilke-Mounts S, Senior AE. Investigation of the role of glutamine-471 and glutamine-1114 in the two catalytic sites of P-glycoprotein. Biochemistry. 2000;39:11921–7. doi: 10.1021/bi001220s 11009605

[63] Zolnerciks JK, Wooding C, Linton KJ. Evidence for a Sav1866-like architecture for the human multidrug transporter P-glycoprotein. FASEB J. 2007;21:3937–48. doi: 10.1096/fj.07-8610com 17627029

[64] Zolnerciks JK, Akkaya BG, Snippe M, Chiba P, Seelig A, Linton KJ. The Q loops of the human multidrug resistance transporter ABCB1 are necessary to couple drug binding to the ATP catalytic cycle. FASEB J. 2014;28:4335–46. doi: 10.1096/fj.13-245639 25016028

[65] Loo TW, Clarke DM. Attachment of a ‘molecular spring’ restores drug-stimulated ATPase activity to P-glycoprotein lacking both Q loop glutamines. Biochem Biophys Res Commun. 2017;483:366–70. doi: 10.1016/j.bbrc.2016.12.137 28025146

[66] Patel SK, George LB, Prasanth Kumar S, Highland HN, Jasrai YT, Pandya HA, et al. A computational approach towards the understanding of *Plasmodium falciparum* multidrug resistance protein 1. ISRN Bioinform. 2013;2013:437168. doi: 10.1155/2013/437168 25937947 PMC4393060

[67] Ferreira PE, Holmgren G, Veiga MI, Uhlén P, Kaneko A, Gil JP. PfMDR1: mechanisms of transport modulation by functional polymorphisms. PLoS ONE. 2011;6:e23875. doi: 10.1371/journal.pone.0023875 21912647 PMC3164660

[68] Jin MS, Oldham ML, Zhang Q, Chen J. Crystal structure of the multidrug transporter P-glycoprotein from *Caenorhabditis elegans*. Nature. 2012;490:566–9. doi: 10.1038/nature11448 23000902 PMC3482266

[69] Kim Y, Chen J. Molecular structure of human P-glycoprotein in the ATP-bound, outward-facing conformation. Science (New York, NY). 2018;359:915–9. doi: 10.1126/science.aar7389 29371429

[70] Reed MB, Saliba KJ, Caruana SR, Kirk K, Cowman AF. Pgh1 modulates sensitivity and resistance to multiple antimalarials in *Plasmodium falciparum*. Nature. 2000;403:906–9. doi: 10.1038/35002615 10706290

[71] Johnson DJ, Fidock DA, Mungthin M, Lakshmanan V, Sidhu AB, Bray PG, et al. Evidence for a central role for PfCRT in conferring *Plasmodium falciparum* resistance to diverse antimalarial agents. Mol Cell. 2004;15:867–77. doi: 10.1016/j.molcel.2004.09.012 15383277 PMC2943419

[72] Lakshmanan V, Bray PG, Verdier-Pinard D, Johnson DJ, Horrocks P, Muhle RA, et al. A critical role for PfCRT K76T in *Plasmodium falciparum* verapamil-reversible chloroquine resistance. EMBO J. 2005;24:2294–305. doi: 10.1038/sj.emboj.7600681 15944738 PMC1173140

[73] Baniecki ML, Wirth DF, Clardy J. High-throughput *Plasmodium falciparum* growth assay for malaria drug discovery. Antimicrob Agents Chemother. 2007;51:716–23. doi: 10.1128/AAC.01144-06 17116676 PMC1797774

[74] Van Tyne D, Park DJ, Schaffner SF, Neafsey DE, Angelino E, Cortese JF, et al. Identification and functional validation of the novel antimalarial resistance locus PF10_0355 in *Plasmodium falciparum*. PLoS Genet. 2011;7:e1001383. doi: 10.1371/journal.pgen.1001383 21533027 PMC3080868

[75] Yuan J, Cheng KC, Johnson RL, Huang R, Pattaradilokrat S, Liu A, et al. Chemical genomic profiling for antimalarial therapies, response signatures, and molecular targets. Science (New York, NY). 2011;333:724–9. 10.1126/science.1205216 PMC3396183 21817045

[76] Chugh M, Scheurer C, Sax S, Bilsland E, van Schalkwyk DA, Wicht KJ, et al. Identification and deconvolution of cross-resistance signals from antimalarial compounds using multidrug-resistant *Plasmodium falciparum* strains. Antimicrob Agents Chemother. 2015;59:1110–8. doi: 10.1128/AAC.03265-14 25487796 PMC4335906

[77] Eastman RT, Khine P, Huang R, Thomas CJ, Su XZ. PfCRT and PfMDR1 modulate interactions of artemisinin derivatives and ion channel blockers. Sci Rep. 2016;6:25379. doi: 10.1038/srep25379 27147113 PMC4857081

[78] Mehlotra RK, Fujioka H, Roepe PD, Janneh O, Ursos LM, Jacobs-Lorena V, et al. Evolution of a unique *Plasmodium falciparum* chloroquine-resistance phenotype in association with *pfcrt* polymorphism in Papua New Guinea and South America. Proc Natl Acad Sci U S A. 2001;98:12689–94. doi: 10.1073/pnas.221440898 11675500 PMC60115

[79] Mu J, Ferdig MT, Feng X, Joy DA, Duan J, Furuya T, et al. Multiple transporters associated with malaria parasite responses to chloroquine and quinine. Mol Microbiol. 2003;49:977–89. doi: 10.1046/j.1365-2958.2003.03627.x 12890022

[80] Sá JM, Twu O, Hayton K, Reyes S, Fay MP, Ringwald P, et al. Geographic patterns of *Plasmodium falciparum* drug resistance distinguished by differential responses to amodiaquine and chloroquine. Proc Natl Acad Sci U S A. 2009;106:18883–9. doi: 10.1073/pnas.0911317106 19884511 PMC2771746

[81] Mu J, Myers RA, Jiang H, Liu S, Ricklefs S, Waisberg M, et al. *Plasmodium falciparum* genome-wide scans for positive selection, recombination hot spots and resistance to antimalarial drugs. Nat Genet. 2010;42:268–71. doi: 10.1038/ng.528 20101240 PMC2828519

[82] Valderramos SG, Valderramos JC, Musset L, Purcell LA, Mercereau-Puijalon O, Legrand E, et al. Identification of a mutant PfCRT-mediated chloroquine tolerance phenotype in *Plasmodium falciparum*. PLoS Pathog. 2010;6:e1000887. doi: 10.1371/journal.ppat.1000887 20485514 PMC2869323

[83] Sanchez CP, Mayer S, Nurhasanah A, Stein WD, Lanzer M. Genetic linkage analyses redefine the roles of PfCRT and PfMDR1 in drug accumulation and susceptibility in *Plasmodium falciparum*. Mol Microbiol. 2011;82:865–78. doi: 10.1111/j.1365-2958.2011.07855.x 21999470

[84] Griffin CE, Hoke JM, Samarakoon U, Duan J, Mu J, Ferdig MT, et al. Mutation in the *Plasmodium falciparum* CRT protein determines the stereospecific activity of antimalarial cinchona alkaloids. Antimicrob Agents Chemother. 2012;56:5356–64. doi: 10.1128/AAC.05667-11 22869567 PMC3457399

[85] Reiling SJ, Rohrbach P. Monitoring PfMDR1 transport in *Plasmodium falciparum*. Malar J. 2015;14:270. doi: 10.1186/s12936-015-0791-3 26169590 PMC4501111

[86] Ross LS, Dhingra SK, Mok S, Yeo T, Wicht KJ, Kumpornsin K, et al. Emerging Southeast Asian PfCRT mutations confer *Plasmodium falciparum* resistance to the first-line antimalarial piperaquine. Nat Commun. 2018;9:3314. doi: 10.1038/s41467-018-05652-0 30115924 PMC6095916

[87] Friedrich O, Reiling SJ, Wunderlich J, Rohrbach P. Assessment of *Plasmodium falciparum* PfMDR1 transport rates using Fluo-4. J Cell Mol Med. 2014;18:1851–62. doi: 10.1111/jcmm.12313 24889967 PMC4196660

[88] Preechapornkul P, Imwong M, Chotivanich K, Pongtavornpinyo W, Dondorp AM, Day NP, et al. *Plasmodium falciparum pfmdr1* amplification, mefloquine resistance, and parasite fitness. Antimicrob Agents Chemother. 2009;53:1509–15. doi: 10.1128/AAC.00241-08 19164150 PMC2663078

[89] Sidhu ABS, Uhlemann A-C, Valderramos SG, Valderramos J-C, Krishna S, Fidock DA. Decreasing *pfmdr1* copy number in *Plasmodium falciparum* malaria heightens susceptibility to mefloquine, lumefantrine, halofantrine, quinine, and artemisinin. J Infect Dis. 2006;194:528–35. doi: 10.1086/507115 16845638 PMC2978021

[90] Deane KJ, Summers RL, Lehane AM, Martin RE, Barrow RA. Chlorpheniramine analogues reverse chloroquine resistance in *Plasmodium falciparum* by inhibiting PfCRT. ACS Med Chem Lett. 2014;5:576–81. doi: 10.1021/ml5000228 24900883 PMC4027738

[91] Martin RE, Butterworth AS, Gardiner DL, Kirk K, McCarthy JS, Skinner-Adams TS. Saquinavir inhibits the malaria parasite’s chloroquine resistance transporter. Antimicrob Agents Chemother. 2012;56:2283–9. doi: 10.1128/AAC.00166-12 22354298 PMC3346596

[92] Hrycyna CA, Summers RL, Lehane AM, Pires MM, Namanja H, Bohn K, et al. Quinine dimers are potent inhibitors of the *Plasmodium falciparum* chloroquine resistance transporter and are active against quinoline-resistant *P*. *falciparum*. ACS Chem Biol. 2014;9:722–30. doi: 10.1021/cb4008953 24369685 PMC4068143

[93] Cowman AF, Karcz S, Galatis D, Culvenor JG. A P-glycoprotein homologue of *Plasmodium falciparum* is localized on the digestive vacuole. J Cell Biol. 1991;113:1033–42. doi: 10.1083/jcb.113.5.1033 1674943 PMC2289011

[94] Rohrbach P, Sanchez CP, Hayton K, Friedrich O, Patel J, Sidhu AB, et al. Genetic linkage of *pfmdr1* with food vacuolar solute import in *Plasmodium falciparum*. EMBO J. 2006;25:3000–11. doi: 10.1038/sj.emboj.7601203 16794577 PMC1500988

[95] Cowell AN, Istvan ES, Lukens AK, Gomez-Lorenzo MG, Vanaerschot M, Sakata-Kato T, et al. Mapping the malaria parasite druggable genome by using *in vitro* evolution and chemogenomics. Science (New York, NY). 2018;359:191–9. doi: 10.1126/science.aan4472 29326268 PMC5925756

[96] Sullivan DJ Jr, Gluzman IY, Russell DG, Goldberg DE. On the molecular mechanism of chloroquine’s antimalarial action. Proc Natl Acad Sci U S A. 1996;93:11865–70. doi: 10.1073/pnas.93.21.11865 8876229 PMC38150

[97] Zhang Y, Hempelmann E. Lysis of malarial parasites and erythrocytes by ferriprotoporphyrin IX-chloroquine and the inhibition of this effect by proteins. Biochem Pharmacol. 1987;36:1267–73. doi: 10.1016/0006-2952(87)90080-3 3297071

[98] Fitch CD, Chevli R, Banyal HS, Phillips G, Pfaller MA, Krogstad DJ. Lysis of *Plasmodium falciparum* by ferriprotoporphyrin IX and a chloroquine-ferriprotoporphyrin IX complex. Antimicrob Agents Chemother. 1982;21:819–22. doi: 10.1128/AAC.21.5.819 7049079 PMC182018

[99] Veiga MI, Dhingra SK, Henrich PP, Straimer J, Gnadig N, Uhlemann AC, et al. Globally prevalent PfMDR1 mutations modulate *Plasmodium falciparum* susceptibility to artemisinin-based combination therapies. Nat Commun. 2016;7:11553. doi: 10.1038/ncomms11553 27189525 PMC4873939

[100] Martin RE, Shafik SH, Richards SN. Mechanisms of resistance to the partner drugs of artemisinin in the malaria parasite. Curr Opin Pharmacol. 2018;42:71–80. doi: 10.1016/j.coph.2018.07.010 30142480

[101] Bohorquez E, Chua M, Meshnick S. Quinine localizes to a non-acidic compartment within the food vacuole of the malaria parasite *Plasmodium falciparum*. Malar J. 2012;11:350. doi: 10.1186/1475-2875-11-350 23088166 PMC3520729

[102] Calçada C, Silva M, Baptista V, Thathy V, Silva-Pedrosa R, Granja D, et al. Expansion of a specific *Plasmodium falciparum* PfMDR1 haplotype in Southeast Asia with increased substrate transport. MBio. 2020;11:e02093–20. doi: 10.1128/mBio.02093-20 33262257 PMC7733942

[103] Godfrey EW, Sanders GE. Effect of water hardness on oocyte quality and embryo development in the African clawed frog (*Xenopus laevis*). Comp Med. 2004;54:170–5. 15134362

[104] Schultz TW, Dawson DA. Housing and husbandry of *Xenopus* for oocyte production. Lab Anim (NY). 2003;32:34–9. doi: 10.1038/laban0203-34 12545183

[105] Bröer S, Rahman B, Pellegri G, Pellerin L, Martin JL, Verleysdonk S, et al. Comparison of lactate transport in astroglial cells and monocarboxylate transporter 1 (MCT 1) expressing *Xenopus laevis* oocytes. Expression of two different monocarboxylate transporters in astroglial cells and neurons. J Biol Chem. 1997;272:30096–102. doi: 10.1074/jbc.272.48.30096 9374487

[106] Saliba KJ, Martin RE, Bröer A, Henry RI, Siobhan McCarthy C, Downie MJ, et al. Sodium-dependent uptake of inorganic phosphate by the intracellular malaria parasite. Nature. 2006;443:582–5. doi: 10.1038/nature05149 17006451

[107] Hayward R, Saliba KJ, Kirk K. The pH of the digestive vacuole of *Plasmodium falciparum* is not associated with chloroquine resistance. J Cell Sci. 2006;119:1016–25. doi: 10.1242/jcs.02795 16492710

[108] Klonis N, Tan O, Jackson K, Goldberg D, Klemba M, Tilley L. Evaluation of pH during cytostomal endocytosis and vacuolar catabolism of haemoglobin in *Plasmodium falciparum*. Biochem J. 2007;407:343–54. doi: 10.1042/BJ20070934 17696875 PMC2275073

[109] Taylor MA, Smith LD. Accumulation of free amino acids in growing *Xenopus laevis* oocytes. Dev Biol. 1987;124:287–90. doi: 10.1016/0012-1606(87)90480-5 2889640

[110] Newmeyer DD, Lucocq JM, Bürglin TR, De Robertis EM. Assembly *in vitro* of nuclei active in nuclear protein transport: ATP is required for nucleoplasmin accumulation. EMBO J. 1986;5:501–10. 3709518 10.1002/j.1460-2075.1986.tb04239.x PMC1166791

[111] Gribble F, Loussouarn G, Tucker S, Zhao C, Nichols C, Ashcroft F. A novel method for measurement of submembrane ATP concentration. J Biol Chem. 2000;275:30046–9. doi: 10.1074/jbc.M001010200 10866996

[112] Schneider CA, Rasband WS, Eliceiri KW. NIH Image to ImageJ: 25 years of image analysis. Nat Methods. 2012;9:671–5. doi: 10.1038/nmeth.2089 22930834 PMC5554542

[113] Zhang V, Kucharski R, Landers C, Richards SN, Bröer S, Martin RE, et al. Characterization of a dopamine transporter and Its splice variant reveals novel features of dopaminergic regulation in the honey bee. Front Physiol. 2019;10. doi: 10.3389/fphys.2019.01375 31736791 PMC6838227

[114] Allen RJ, Kirk K. *Plasmodium falciparum* culture: the benefits of shaking. Mol Biochem Parasitol. 2010;169:63–5. doi: 10.1016/j.molbiopara.2009.09.005 19766147

[115] Drexler HG, Uphoff CC. Mycoplasma contamination of cell cultures: incidence, sources, effects, detection, elimination, prevention. Cytotechnology. 2002;39:75–90. doi: 10.1023/A:1022913015916 19003295 PMC3463982

[116] Smilkstein M, Sriwilaijaroen N, Kelly JX, Wilairat P, Riscoe M. Simple and inexpensive fluorescence-based technique for high-throughput antimalarial drug screening. Antimicrob Agents Chemother. 2004;48:1803–6. doi: 10.1128/AAC.48.5.1803-1806.2004 15105138 PMC400546

[117] Spry C, Macuamule C, Lin Z, Virga KG, Lee RE, Strauss E, et al. Pantothenamides are potent, on-target inhibitors of *Plasmodium falciparum* growth when serum pantetheinase is inactivated. PLoS ONE. 2013;8:e54974. doi: 10.1371/journal.pone.0054974 23405100 PMC3566143

[118] Thompson JD, Higgins DG, Gibson TJ. CLUSTAL W: improving the sensitivity of progressive multiple sequence alignment through sequence weighting, position-specific gap penalties and weight matrix choice. Nucleic Acids Res. 1994;22:4673–80. doi: 10.1093/nar/22.22.4673 7984417 PMC308517

[119] Sali A, Blundell T. Comparative protein modelling by satisfaction of spatial restraints. Protein Struct Distance Anal. 1994;64. 10.1006/jmbi.1993.1626 8254673

[120] Shen MY, Sali A. Statistical potential for assessment and prediction of protein structures. Protein Sci. 2006;15:2507–24. doi: 10.1110/ps.062416606 17075131 PMC2242414

[121] Humphrey W, Dalke A, Schulten K. VMD: Visual molecular dynamics. J Mol Graph. 1996;14:33–8. doi: 10.1016/0263-7855(96)00018-5 8744570

[122] Vermaas J, Hardy D, Stone J, Tajkhorshid E, Kohlmeyer A. TopoGromacs: Automated topology conversion from CHARMM to Gromacs within VMD. J Chem Inf Model. 2016;56. doi: 10.1021/acs.jcim.6b00103 27196035 PMC5543333

[123] Vanommeslaeghe K, Hatcher E, Acharya C, Kundu S, Zhong S, Shim J, et al. CHARMM general force field: A force field for drug-like molecules compatible with the CHARMM all-atom additive biological force fields. J Comput Chem. 2010;31:671–90. doi: 10.1002/jcc.21367 19575467 PMC2888302

[124] Vanommeslaeghe K, Raman EP, MacKerell AD Jr. Automation of the CHARMM General Force Field (CGenFF) II: assignment of bonded parameters and partial atomic charges. J Chem Inf Model. 2012;52:3155–68. doi: 10.1021/ci3003649 23145473 PMC3528813

[125] Vanommeslaeghe K, MacKerell AD Jr. Automation of the CHARMM General Force Field (CGenFF) I: bond perception and atom typing. J Chem Inf Model. 2012;52:3144–54. doi: 10.1021/ci300363c 23146088 PMC3528824

[126] Best RB, Zhu X, Shim J, Lopes PE, Mittal J, Feig M, et al. Optimization of the additive CHARMM all-atom protein force field targeting improved sampling of the backbone φ, ψ and side-chain χ(1) and χ(2) dihedral angles. J Chem Theory Comput. 2012;8:3257–73. doi: 10.1021/ct300400x 23341755 PMC3549273

[127] Foley M, Tilley L. Quinoline antimalarials: mechanisms of action and resistance and prospects for new agents. Pharmacol Therapeut. 1998;79:55–87. doi: 10.1016/s0163-7258(98)00012-6 9719345

[128] vonHeijne G. Control of topology and mode of assembly of a polytopic membrane protein by positively charged residues. Nature. 1989;341:456–8. doi: 10.1038/341456a0 2677744

[129] von Heijne G. Membrane-protein topology. Nat Rev Mol Cell Biol. 2006;7:909–18. doi: 10.1038/nrm2063 17139331

[130] Kuhn Y, Sanchez CP, Ayoub D, Saridaki T, Van Dorsselaer A, Lanzer M. Trafficking of the phosphoprotein PfCRT to the digestive vacuolar membrane in *Plasmodium falciparum*. Traffic. 2010;11:236–49. doi: 10.1111/j.1600-0854.2009.01018.x 20015114

[131] Summers RL, Martin RE. Functional characteristics of the malaria parasite’s "chloroquine resistance transporter": implications for chemotherapy. Virulence. 2010;1:304–8. doi: 10.4161/viru.1.4.12012 21178460

