# Supplementary figures and images for: Mechanistic basis for multidrug resistance and collateral drug sensitivity conferred to the malaria parasite by polymorphisms in PfMDR1 and PfCRT
Source: PLoS Biol. 2022 May 4;20(5):e3001616. doi: 10.1371/journal.pbio.3001616 (PMC9067703; doi:10.1371/journal.pbio.3001616)

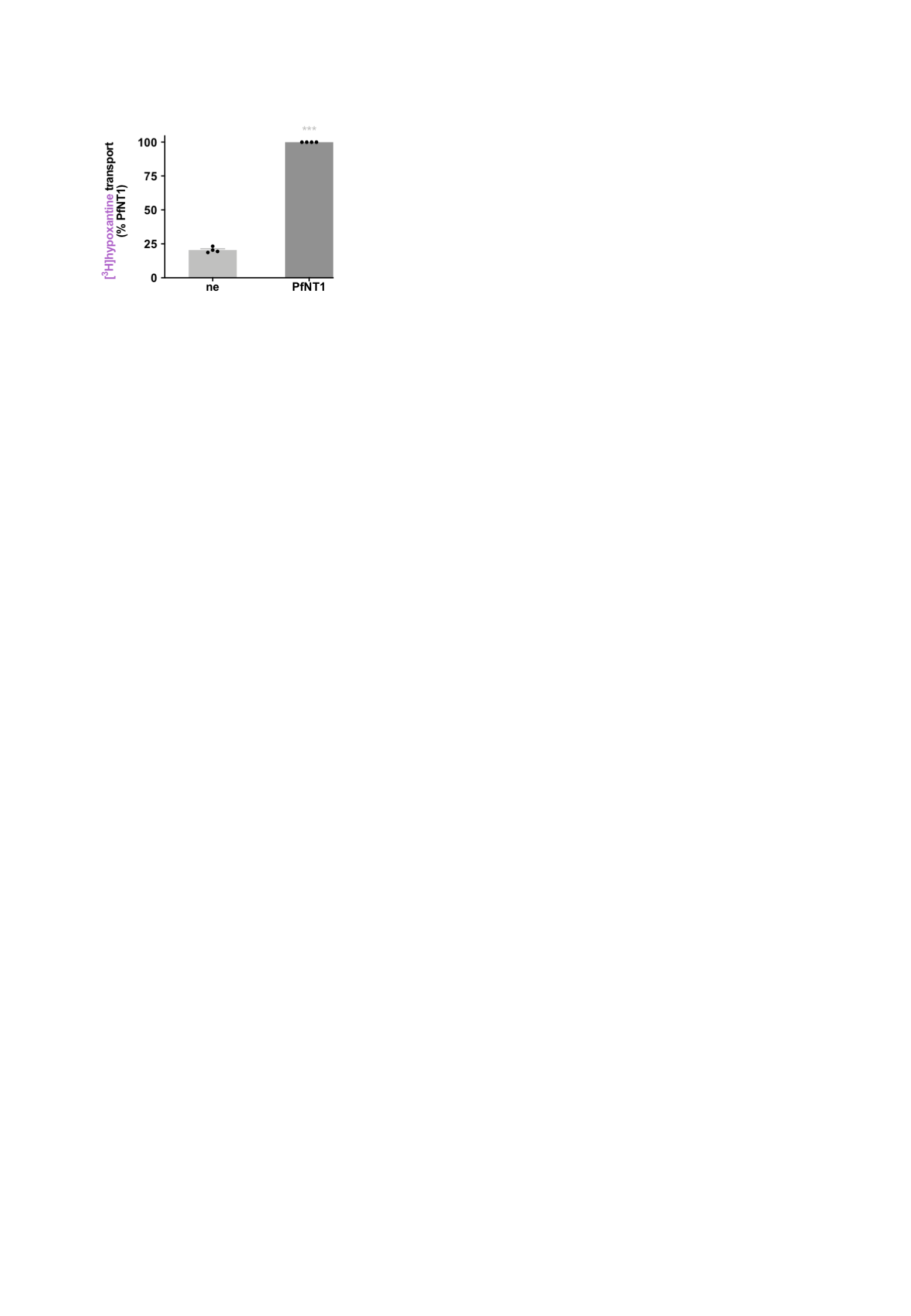

Supplement: S1 Fig — Oocytes expressing PfNT1 were used as a negative control in the experiments measuring [3H]drug efflux from oocytes expressing human P-gp or PfMDR1 and in the experiments measuring [3H]lumefantrine or [3H]VDPVNF efflux from oocytes expressing PfCRT. This control demonstrated that the heterologous expression of a transporter in Xenopus oocytes does not affect the ability of the oocyte membrane to reseal following the microinjection of a [3H]drug or [3H]VDPVNF. To confirm that PfNT1 was expressed and functional at the oocyte plasma membrane, the uptake of hypoxanthine (a known substrate of PfNT1) was measured in oocytes expressing the transporter, as well as in nonexpressing (ne) oocytes. The low level of [3H]hypoxanthine accumulation in ne is due to the simple diffusion of the unprotonated species. The data are the mean of n = 4 independent experiments, each yielding similar results and overlaid as individual data points, and the error is the SEM. The asterisks denote a significant difference from ne; ***P < 0.001 (1-way ANOVA). The data underlying this figure is supplied in S3 Data. ne, nonexpressing oocytes; PfCRT, Plasmodium falciparum chloroquine resistance transporter; PfNT1, Plasmodium falciparum nucleoside transporter 1; PfMDR1, Plasmodium falciparum multidrug resistance protein 1; P-gp, P-glycoprotein. (TIF) [file pbio.3001616.s001.tif]

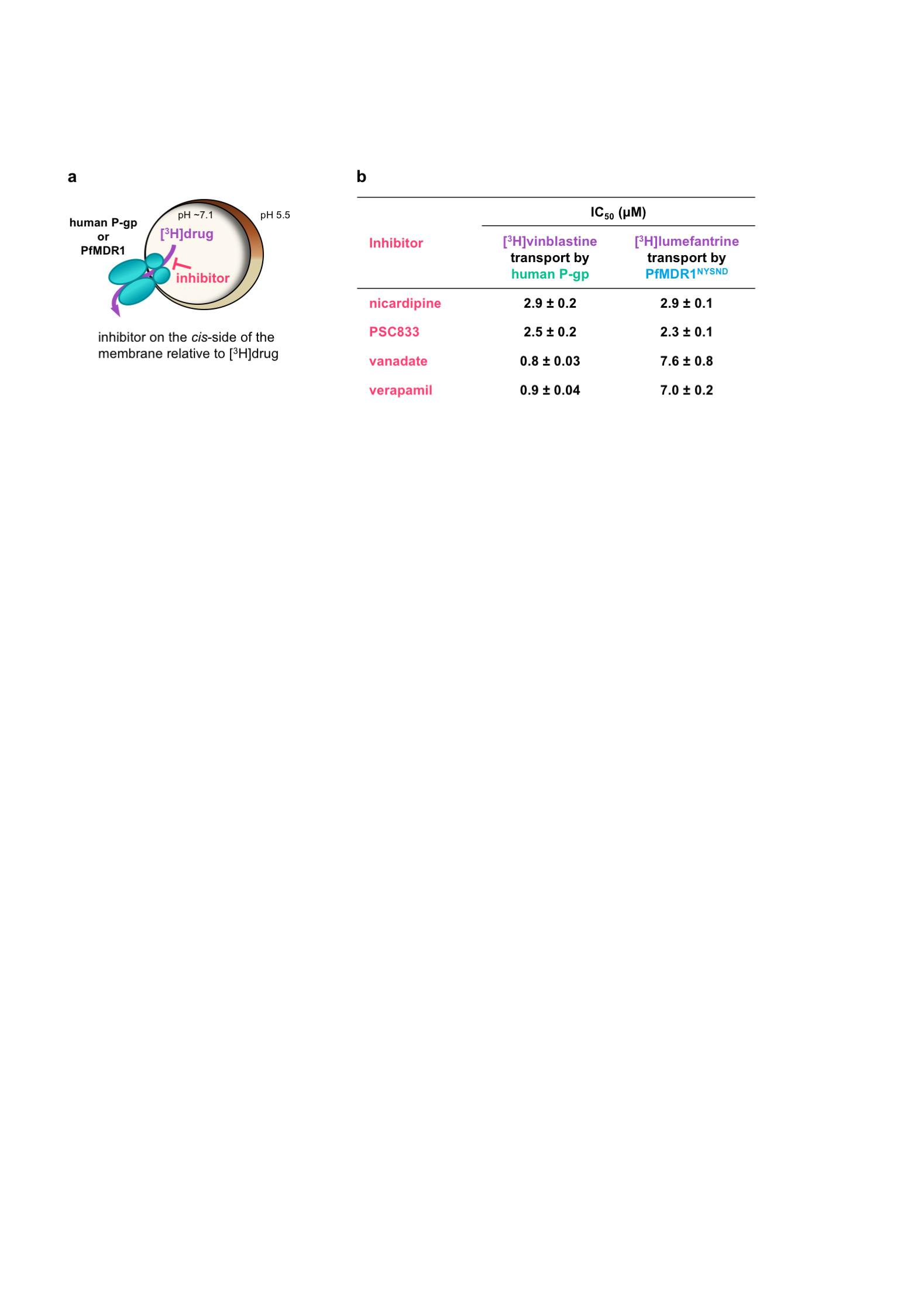

Supplement: S2 Fig — (a) Schematic showing the cis-inhibition of [3H]drug transport via human P-gp or PfMDR1 by known P-gp inhibitors in the Xenopus oocyte system. (b) The IC50 values of nicardipine, PSC833, vanadate, and verapamil against [3H]vinblastine transport via human P-gp and [3H]lumefantrine transport via PfMDR1. The data are the mean of n = 4 independent experiments, and the error is the SEM. The data underlying this figure is supplied in S3 Data. PfMDR1, Plasmodium falciparum multidrug resistance protein 1; P-gp, P-glycoprotein. (TIF) [file pbio.3001616.s002.tif]

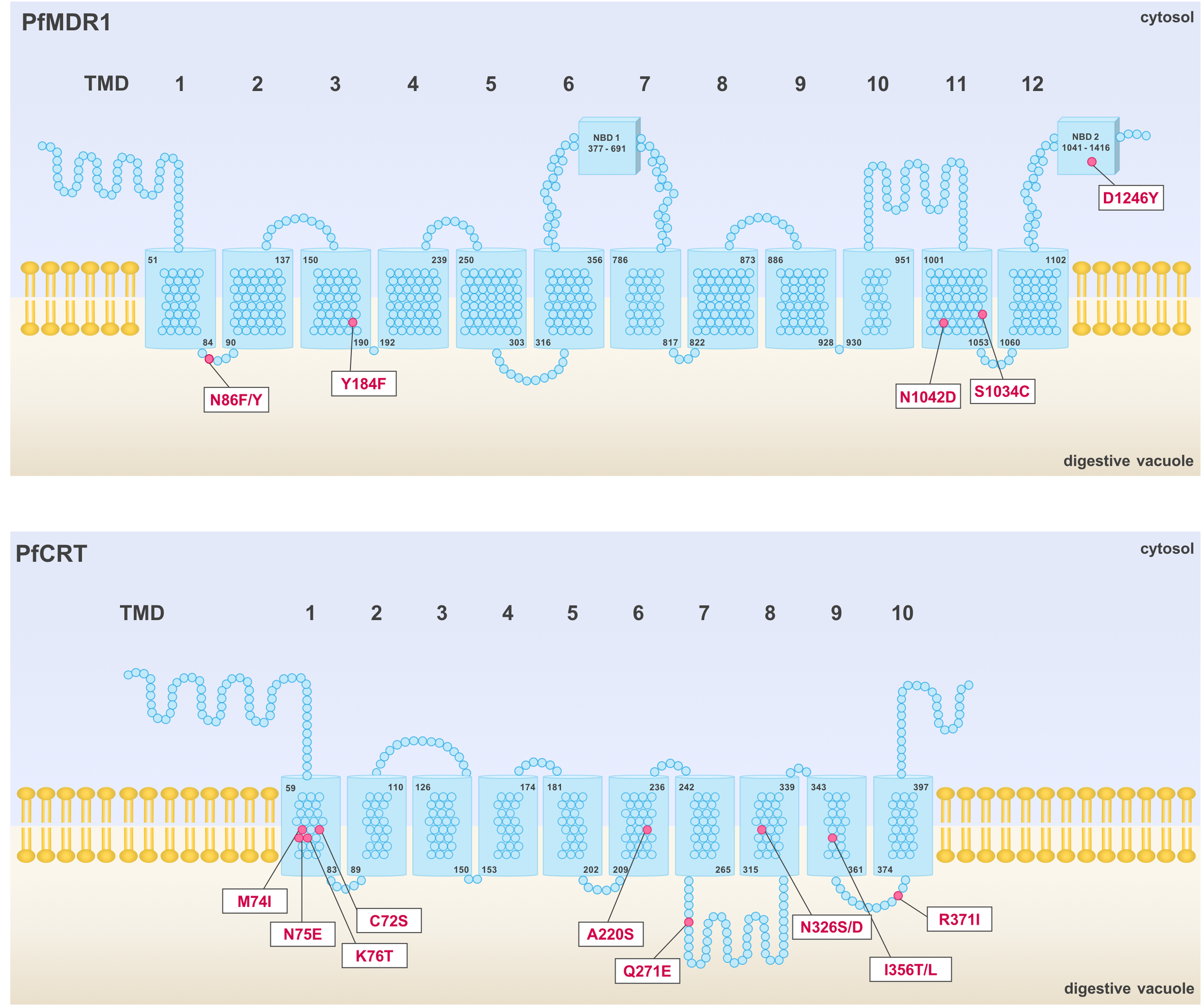

Supplement: S3 Fig — (a) PfMDR1 consists of 1,419 amino acid residues that are arranged into 12 TMDs and 2 NBDs. The length of the loops shown surrounding the NBDs are approximate. (b) PfCRT consists of 424 amino acid residues that are arranged into 10 TMDs. All loops are representative of the true loop length. The positions of the mutations in the isoforms of PfMDR1 and PfCRT used in this study are indicated by pink circles and the box attached to each polymorphic residue lists the (non-wild type) amino acid(s) that occur at that position. NBD, nucleotide-binding domain; PfCRT, Plasmodium falciparum chloroquine resistance transporter; PfMDR1, Plasmodium falciparum multidrug resistance protein 1; TMD, transmembrane domain. (TIF) [file pbio.3001616.s003.tif]

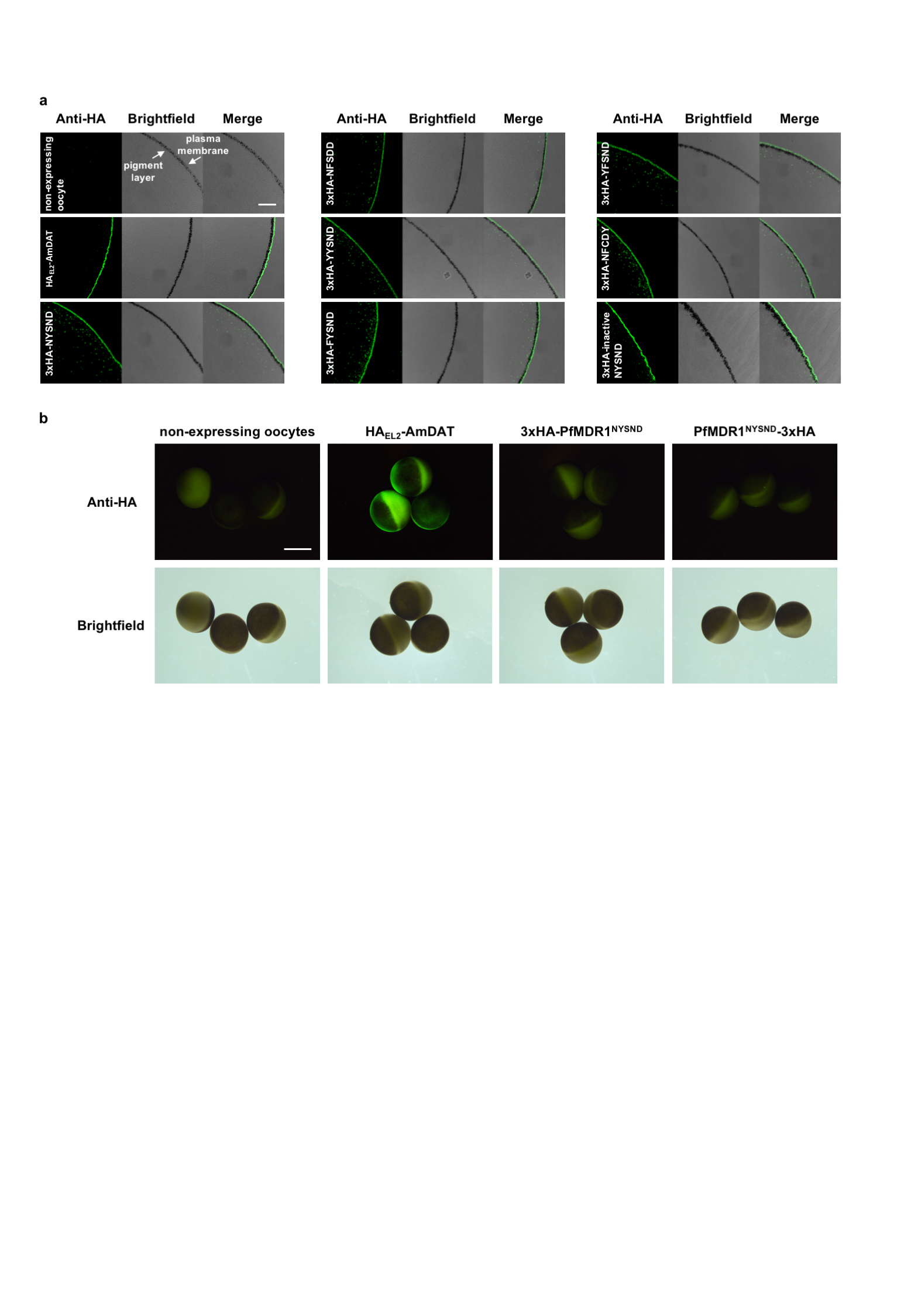

Supplement: S4 Fig — (a) Immunofluorescence microscopy images confirmed that the expression of each of the 3xHA-tagged PfMDR1 isoforms resulted in a fluorescent band external to the pigment layer, indicating that the proteins were expressed in the oocyte plasma membrane. The band was not present in ne. Oocytes expressing a HA-tagged version of the Apis mellifera dopamine transporter (HAEL2-AmDAT) at the plasma membrane serve as a positive control [113]. The length of the scale bar is 50 μm. The images are representative of at least 2 independent experiments (performed using oocytes from different frogs), within which images were obtained from a minimum of 3 oocytes per oocyte type. (b) Immunofluorescence microscopy was used to determine the orientation of PfMDR1 in the plasma membrane of live oocytes using an anti-HA antibody and a fluorescent secondary antibody. The anti-HA antibody will bind to extracellular HA-tags and cannot access those that are intracellular. Hence, only proteins with extracellular HA-tags will be detected by the anti-HA antibody. The fluorescent signal in oocytes expressing HAEL2-AmDAT is at its strongest at the periphery of the oocyte, indicating that AmDAT is orientated in the oocyte membrane such that the HA-tagged loop is extracellular. There was no fluorescent signal in the ne (the negative control) or in oocytes expressing 3xHA-PfMDR1NYSND or PfMDR1NYSND-3xHA (see S1 Text). This indicates that the amino terminus and carboxyl terminus of PfMDR1 are located in the cytosol of the oocyte. The length of the scale bar is 1 mm. The images are representative of 3 independent experiments, within which images were obtained from a minimum of 3 oocytes per oocyte type. HA, hemagglutinin; ne, nonexpressing oocytes; PfMDR1, Plasmodium falciparum multidrug resistance protein 1. (TIFF) [file pbio.3001616.s004.tiff]

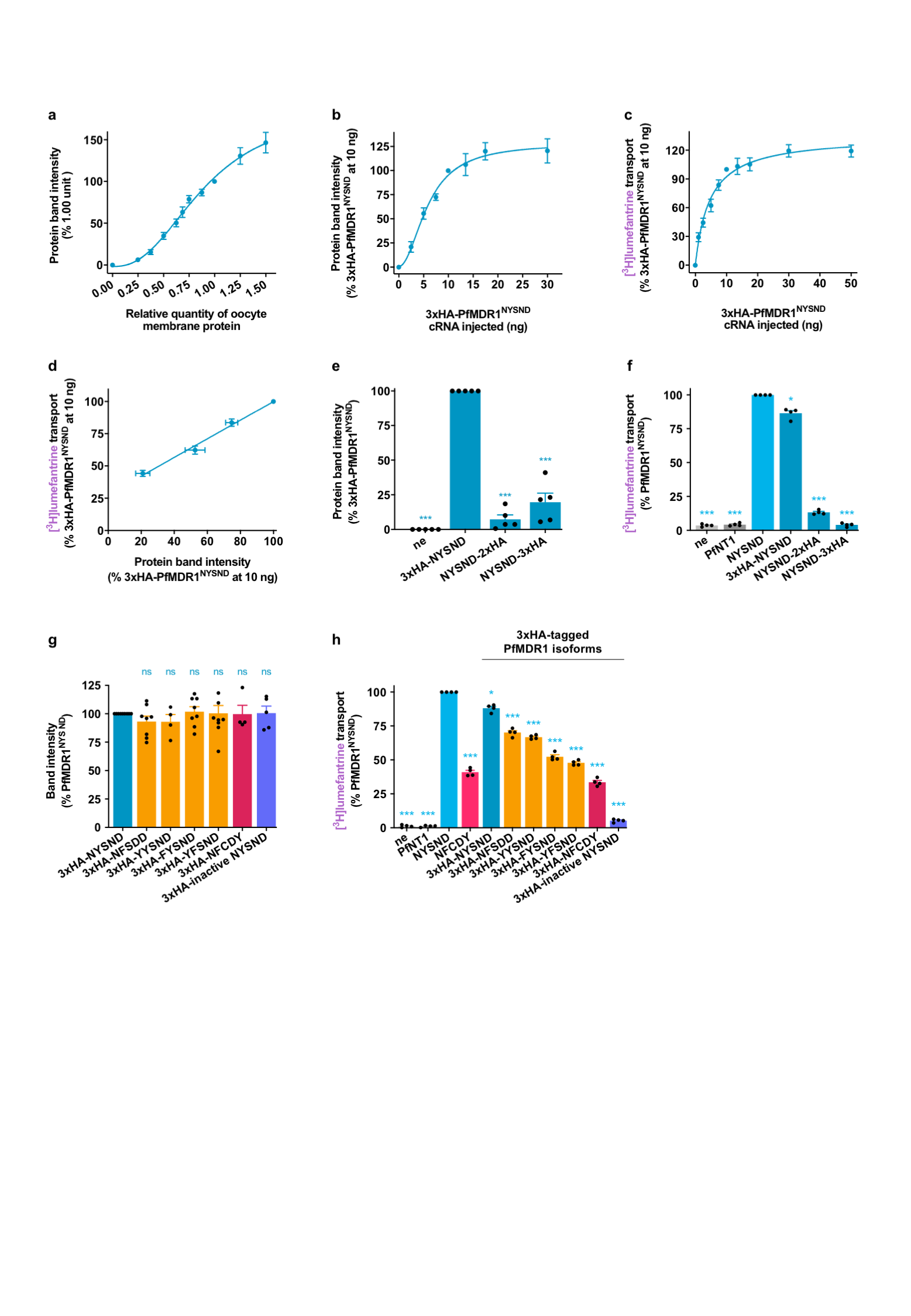

Supplement: S5 Fig — (a) Analysis of the PfMDR1 levels in different dilutions of a membrane protein preparation from 3xHA-PfMDR1NYSND-expressing oocytes revealed a sigmoidal relationship between the relative quantity of membrane protein and the intensity of the corresponding PfMDR1 band (r2 = 0.99). (b) A sigmoidal relationship was observed between the intensity of the 3xHA-PfMDR1NYSND protein band and the amount of 3xHA-PfMDR1NYSND cRNA microinjected into oocytes (r2 = 0.99). The relationship is approximately linear between 2.5 and 10 ng of cRNA. (c) A sigmoidal relationship was observed between the level of [3H]lumefantrine transport and the quantity of 3xHA-PfMDR1NYSND cRNA microinjected into the oocyte (r2 = 0.99). This relationship is linear between 1 and 10 ng of cRNA, with [3H]lumefantrine transport saturating above this range. (d) Combining the plots in panels b and c revealed that in oocytes microinjected with 2.5 to 10 ng of 3xHA-PfMDR13D7 cRNA, the intensity of the 3xHA-PfMDR1NYSND protein band strongly correlates with the level of 3xHA-PfMDR1NYSND-mediated [3H]lumefantrine transport (r2 = 0.9904). (e) Semiquantification of PfMDR1 protein levels in the membranes of oocytes expressing either 3xHA-PfMDR1NYSND, PfMDR1NYSND-2xHA, or PfMDR1NYSND-3xHA revealed that, relative to 3xHA-PfMDR1NYSND, the addition of HA-tags to the carboxyl terminus of the protein significantly reduced PfMDR1 levels. (f) Oocytes expressing PfMDR1NYSND-2xHA or PfMDR1NYSND-3xHA also displayed significantly lower levels of [3H]lumefantrine transport relative to those expressing 3xHA-PfMDR1NYSND. (g) Densitometric analyses of total protein indicated that there were no significant differences between sample lanes in the western blot experiments that gave rise to Fig 1G. In each experiment, the protein on the nitrocellulose membrane was stained with Ponceau S and the intensity between 70 and 180 kDa was measured within each lane. (h) 3xHA-PfMDR1NYSND and 3xHA-PfMDR1NFCDY had slightly reduced lumefantrine trans [file pbio.3001616.s005.tiff]

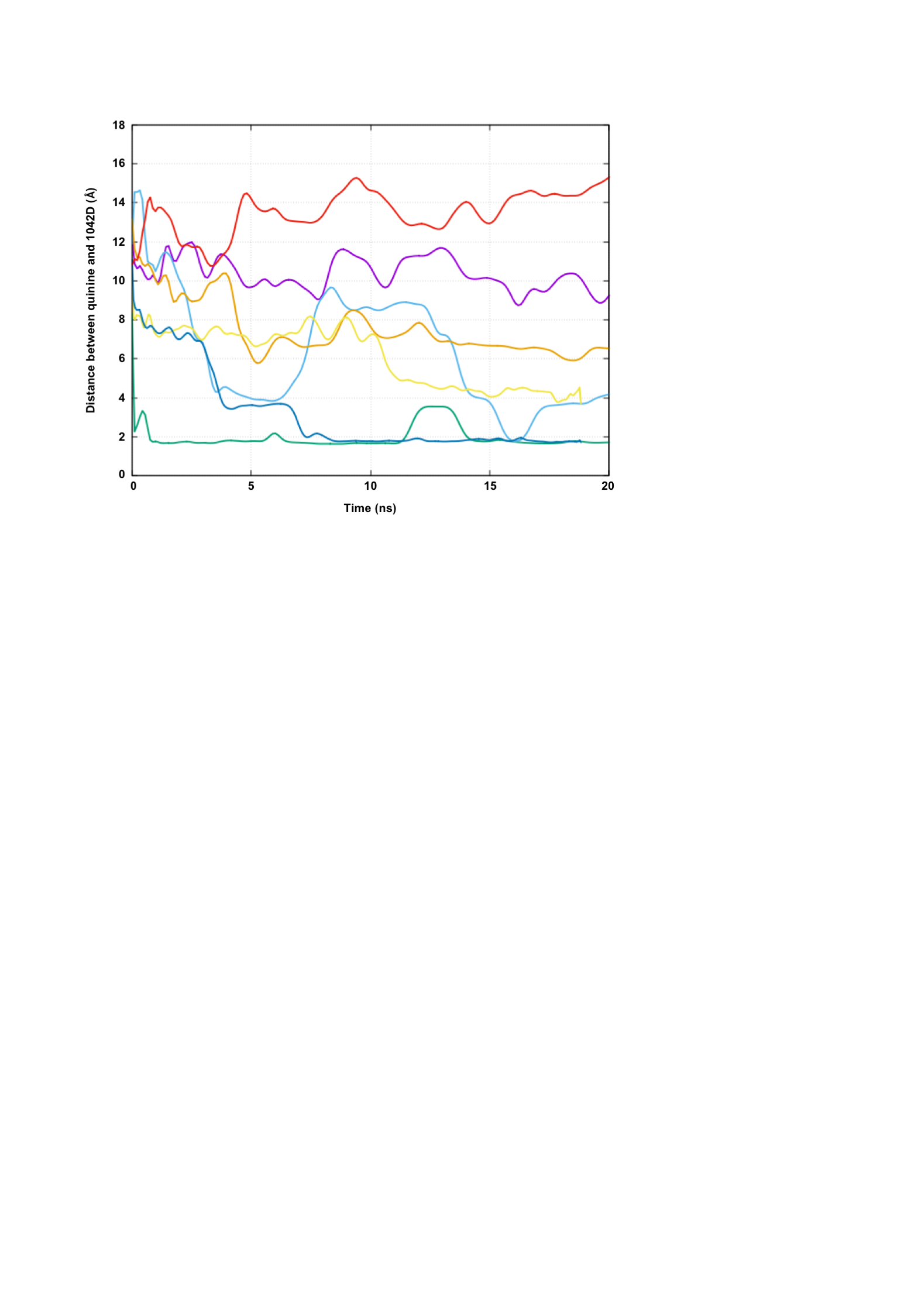

Supplement: S6 Fig — The movement of the quinine molecule during the 7 molecular dynamics simulations is indicated by the distance between the protonated cyclic amine ring of quinine and the carboxylate oxygen atom on the 1042D side chain over the duration of the simulation. Seven simulations were performed, each represented by a different colored line. Quinine moves close to D1042 in 4 of the 7 simulations (denoted by the yellow, blue, navy, and green lines), forming stable, long-lasting hydrogen bonds in 2 of these simulations (denoted by the navy and green lines). PfMDR1, Plasmodium falciparum multidrug resistance protein 1. (TIF) [file pbio.3001616.s006.tif]

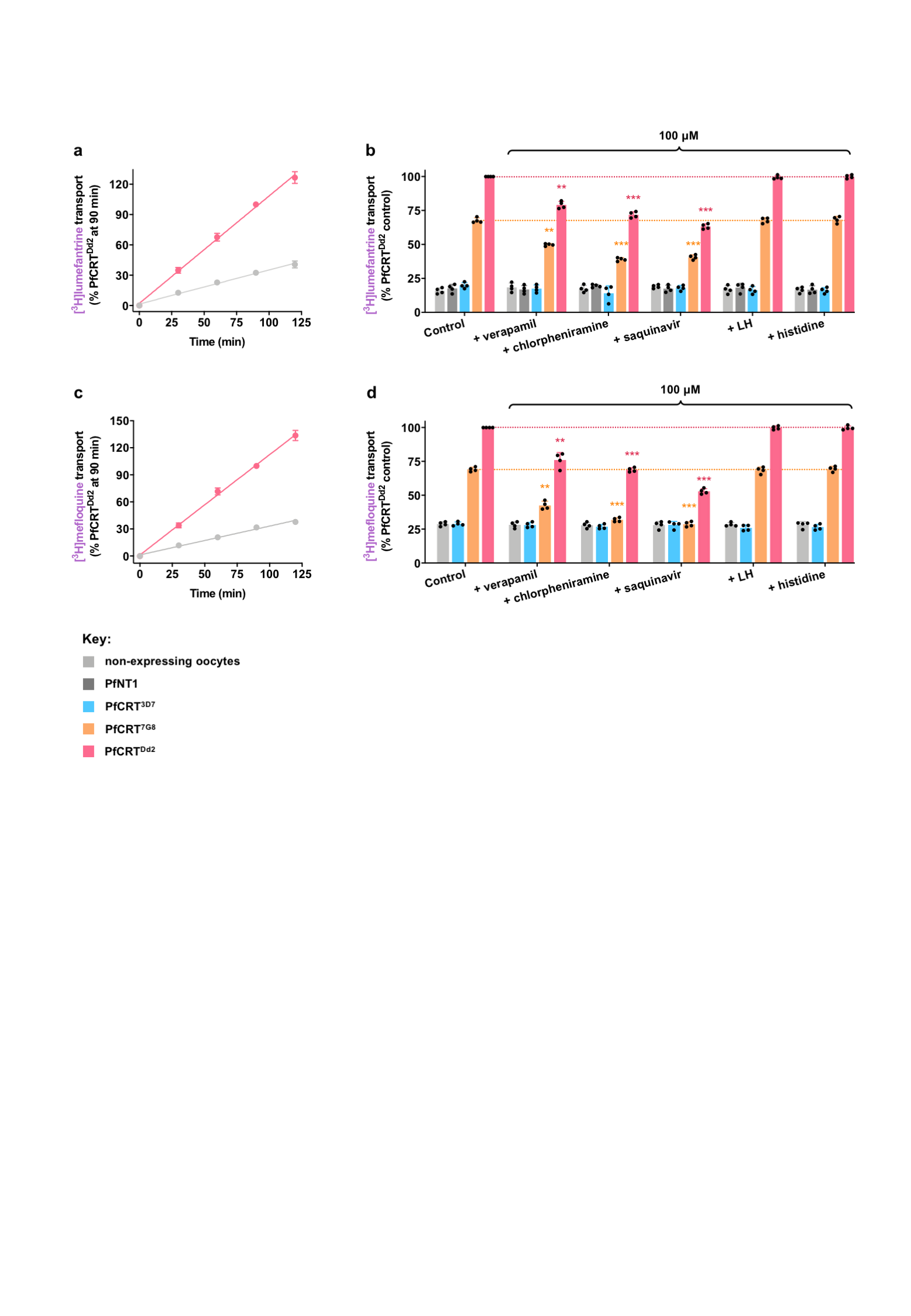

Supplement: S7 Fig — (a, c) The transport of [3H]lumefantrine (a) and [3H]mefloquine (c) via PfCRTDd2 was approximately linear with time for at least 2 hours. (b, d) The transport of [3H]lumefantrine (b) and [3H]mefloquine (d) via PfCRTDd2 and PfCRT7G8 was reduced by known inhibitors of the transporter (verapamil, chlorpheniramine, and saquinavir) and was unaffected by histidine and LH (metabolites that do not interact with PfCRT). The data are the mean of n = 4 independent experiments, each yielding similar results and overlaid as individual data points in panels b and d, and the error is the SEM. Where not visible, the error bars fall within the symbols. The asterisks denote a significant difference from the PfCRTDd2 control (red asterisks) or the PfCRT7G8 control (orange asterisks): **P < 0.01, ***P < 0.001 (1-way ANOVA). The data underlying this figure is supplied in S3 Data. PfCRT, Plasmodium falciparum chloroquine resistance transporter. (TIF) [file pbio.3001616.s007.tif]

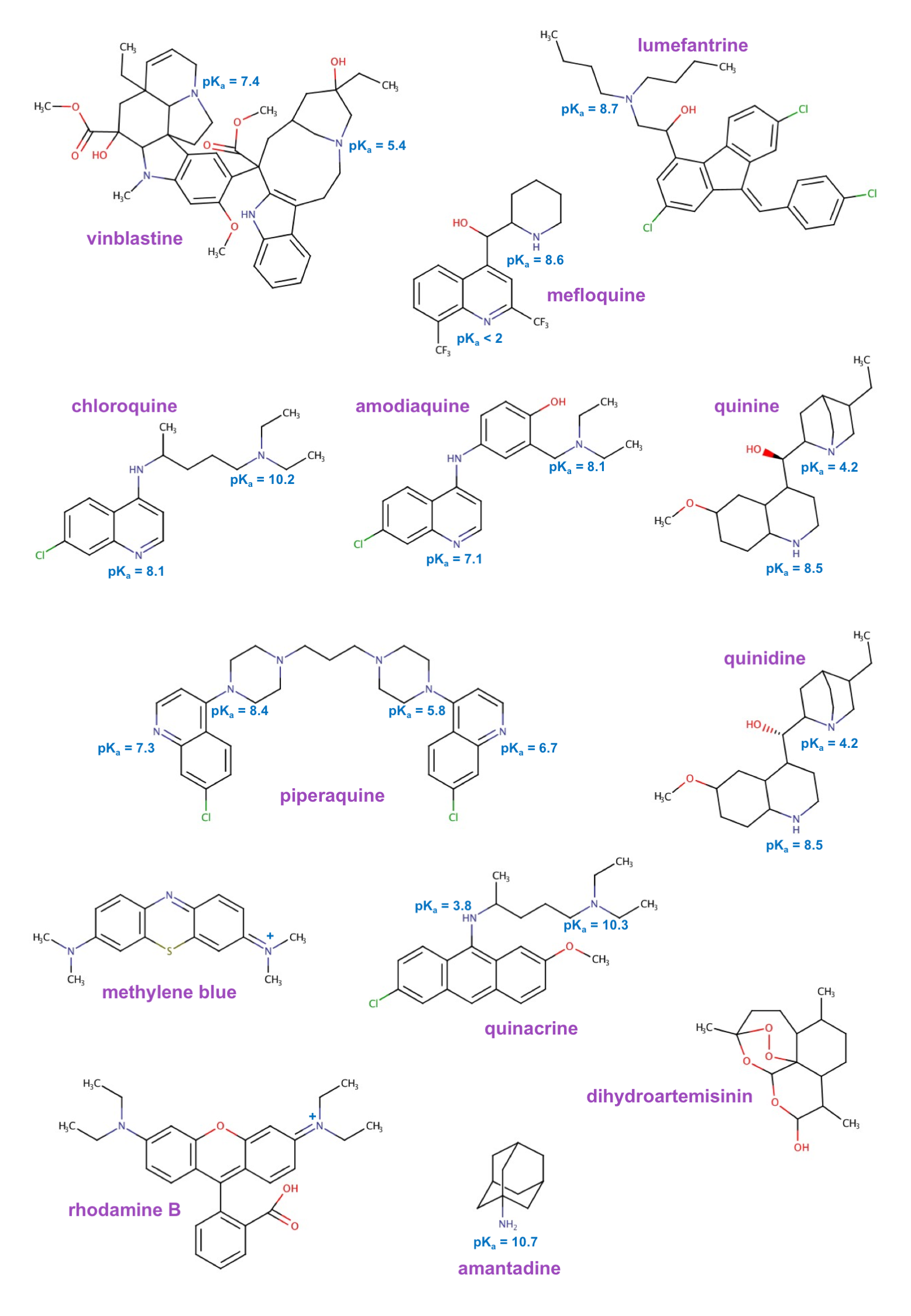

Supplement: S8 Fig — The pKa values for the protonatable nitrogen(s) within each compound are indicated. The structures and pKa values were collated from the literature [127] or generated in the MarvinSketch software (ChemAxon). pKa, the negative logarithm to the base 10 of the acid dissociation constant. (TIF) [file pbio.3001616.s008.tif]

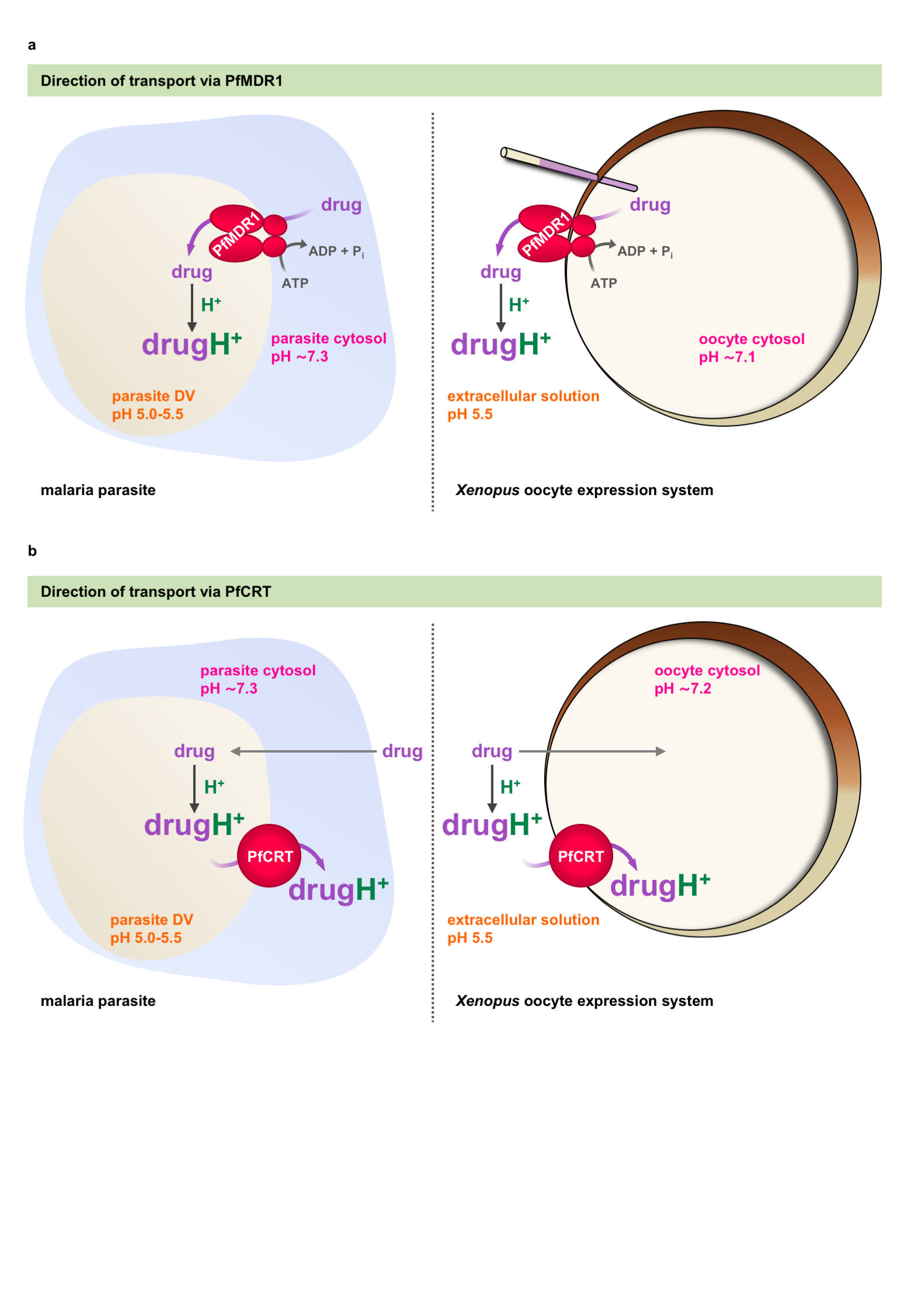

Supplement: S9 Fig — PfMDR1 orientates in the DV membrane such that its amino terminus and carboxyl terminus, as well as its NBDs, are located in the parasite cytosol [36]. It was expected that PfMDR1 would adopt the same orientation in the oocyte plasma membrane (i.e., with its termini and NBDs extending into the oocyte cytosol) due to the “positive inside rule” [128,129]. This orientation was confirmed using immunofluorescence microscopy and live oocytes expressing HA-tagged versions of PfMDR1 (S4 Fig). PfCRT also orientates in the DV membrane with its amino terminus and carboxyl terminus in the parasite cytosol [130] and is predicted, on the basis of the “positive inside rule,” to adopt the same orientation in the oocyte plasma membrane (i.e., with its amino terminus and carboxyl terminus in the oocyte cytosol). (a) In the malaria parasite, PfMDR1 mediates the transport of drugs from the parasite cytosol (pH approximately 7.3) into the acidic environment of the DV (pH 5.0 to 5.5), where the weak base drugs will become protonated [94]. In the Xenopus oocyte system, the drug under study is microinjected into the oocyte cytosol (pH approximately 7.2) and is transported via PfMDR1 into the extracellular solution (pH 5.5). Thus, in both cases, the direction of PfMDR1-mediated drug transport is from the cell cytosol into the acidic DV lumen or the acidic extracellular solution. (b) Weak base drugs also accumulate in the parasite DV via simple diffusion of the neutral species, which becomes protonated upon entering the acidic lumen of this organelle. In parasites that express chloroquine resistance-conferring isoforms of PfCRT, the transporter mediates the transport of the protonated drug from the DV into the parasite cytosol. In the Xenopus oocyte expression system, the drug under study is typically added to the acidic extracellular solution and mutant isoforms of PfCRT transport the protonated drug into the oocyte cytosol. Therefore, in both scenarios, the direction of PfCRT-mediated drug [file pbio.3001616.s009.tif]

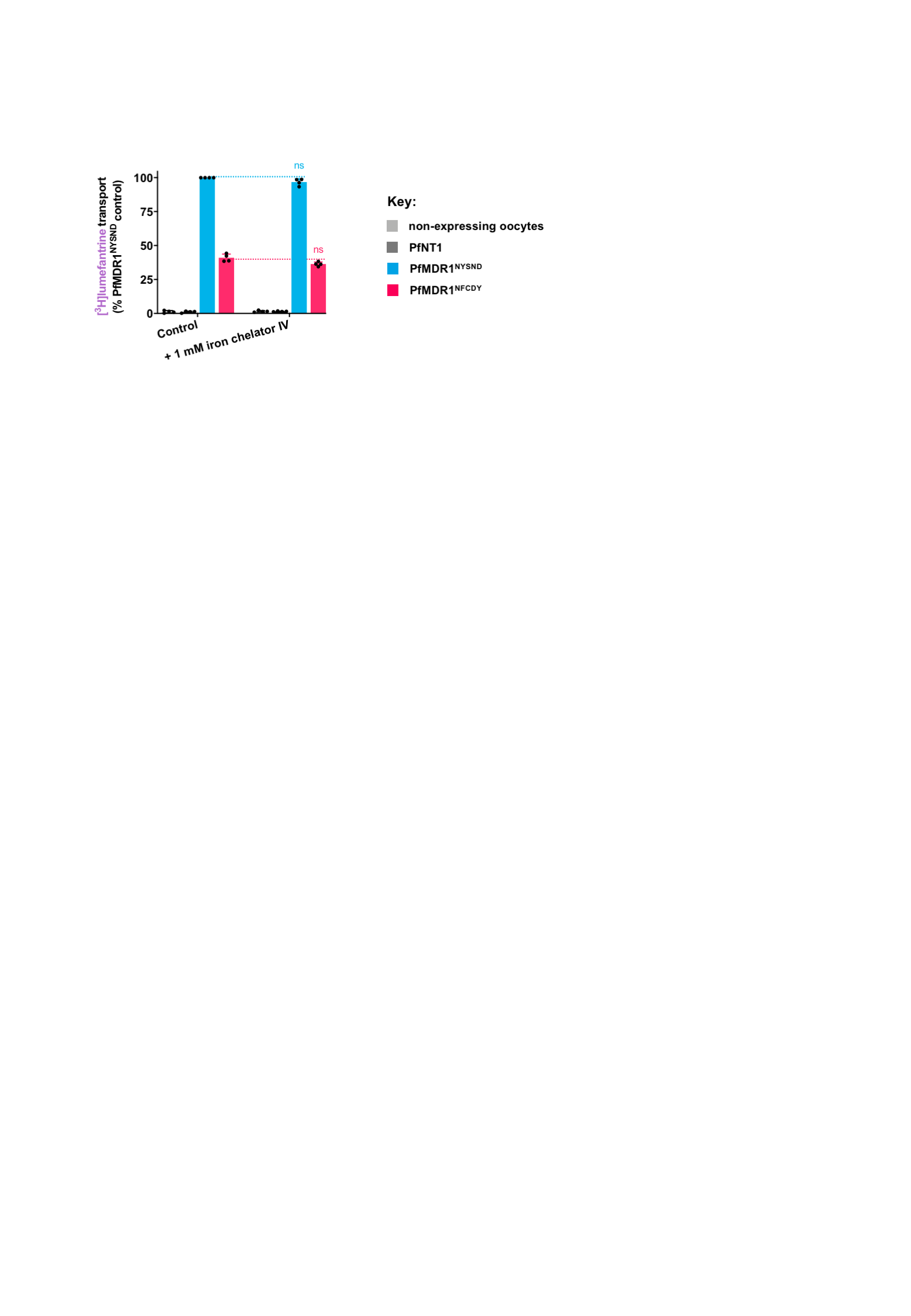

Supplement: S10 Fig — Measurements of [3H]dihydroartemisinin transport via PfMDR1 were made in the presence of the iron chelator IV to bind any free iron present in the oocyte. Free iron could activate dihydroartemisinin, thereby damaging the oocytes and interfering with measurements of transport. The effect of iron chelator IV (estimated intracellular concentration of 1 mM) on [3H]lumefantrine transport was measured in ne and oocytes expressing either PfNT1, PfMDR1NYSND, or PfMDR1NFCDY. The data are the mean of n = 4 independent experiments (each yielding similar results and overlaid as individual data points), and the error is the SEM. Where not visible, the error bars fall within the symbols. Statistical analyses were performed relative to the PfMDR1NYSND control (blue) or the PfMDR1NFCDY (dark pink) control; ns, not significant (1-way ANOVA). The data underlying this figure is supplied in S3 Data. ne, nonexpressing oocytes; PfMDR1, Plasmodium falciparum multidrug resistance protein 1; PfNT1, Plasmodium falciparum nucleoside transporter 1. (TIF) [file pbio.3001616.s010.tif]
