# Supplementary material for: Mechanistic basis for multidrug resistance and collateral drug sensitivity conferred to the malaria parasite by polymorphisms in PfMDR1 and PfCRT
Source: PLoS Biol. 2022 May 4;20(5):e3001616. doi: 10.1371/journal.pbio.3001616 (PMC9067703; doi:10.1371/journal.pbio.3001616)
Supplement: S1 Table — PfMDR1, Plasmodium falciparum multidrug resistance protein 1. (PDF) [file pbio.3001616.s011.pdf]

S1 Table: Amino acid mutations in the PfMDR1 isoforms included for study.

PfMDR1 isoform	Origin	Amino acid position in PfMDR1						
		86	184	588	1034	1042	1246	1337
3D7	Africa and SE Asia	N	Y	E	S	N	D	E
HB3	Honduras	N	F	E	S	D	D	E
Dd2 or K1	Africa	Y	Y	E	S	N	D	E
Dd2	Africa	F	Y	E	S	N	D	E
GB4	Africa	Y	F	E	S	N	D	E
7G8	South America	N	F	E	C	D	Y	E
inactive 3D7	n.a.	N	Y	Q	S	N	D	Q
