# Supplementary material for: Mechanistic basis for multidrug resistance and collateral drug sensitivity conferred to the malaria parasite by polymorphisms in PfMDR1 and PfCRT
Source: PLoS Biol. 2022 May 4;20(5):e3001616. doi: 10.1371/journal.pbio.3001616 (PMC9067703; doi:10.1371/journal.pbio.3001616)
Supplement: S2 Table — PfCRT, Plasmodium falciparum chloroquine resistance transporter. (PDF) [file pbio.3001616.s012.pdf]

S2 Table: Amino acid mutations in the PfCRT isoforms included for study.

PfCRT isoform	Origin	Amino acid position in PfCRT								
		72	74	75	76	220	271	326	356	371
**3D7**	Africa and SE Asia	**C**	**M**	**N**	**K**	**A**	**Q**	**N**	**I**	**R**
**Dd2**	Africa	**C**	**I**	**E**	**T**	**S**	**E**	**S**	**T**	**I**
**K1**	Africa	**C**	**I**	**E**	**T**	**S**	**E**	**S**	**I**	**I**
**GB4**	Africa	**C**	**I**	**E**	**T**	**S**	**E**	**N**	**I**	**I**
**7G8**	South America	**S**	**M**	**N**	**T**	**S**	**Q**	**D**	**L**	**R**
**Ecu1110**	South America	**C**	**M**	**N**	**T**	**S**	**Q**	**D**	**L**	**R**
