# Supplementary material for: Mechanistic basis for multidrug resistance and collateral drug sensitivity conferred to the malaria parasite by polymorphisms in PfMDR1 and PfCRT
Source: PLoS Biol. 2022 May 4;20(5):e3001616. doi: 10.1371/journal.pbio.3001616 (PMC9067703; doi:10.1371/journal.pbio.3001616)
Supplement: S3 Table — (PDF) [file pbio.3001616.s013.pdf]

S3 Table: *In vitro* drug resistance indices of *P. falciparum* strains for lumefantrine, mefloquine, chloroquine, and quinine.

Parasite strain[a]		3D7	HB3/GC03	Dd2	K1	GB4	7G8
PfMDR1 isoform		NYSND	NFSDD	YYSND & FYSND	YYSND	YFSND	NFCDY
		In vitro drug resistance index					
	Valderramos et al. (2010) [1]	1.00		0.48			0.36
	Van Tyne et al. (2011) [2]	1.00	0.83	0.87	0.36		0.36
Lumefantrine[b]	Pelleau et al. (2015) [3]	1.00					0.35
	Eastman et al. (2016) [4]	1.00	1.12	0.79		0.85	0.20
	Ross et al. (2018) [5]	1.00		0.66			
	**Mean ± SEM or range/2 (n)**	**1.00 ± 0.00 (5)**	**0.98 ± 0.15 (2)**	**0.70 ± 0.08 (4)**	**0.36 (1)**	**0.85 (1)**	**0.32 ± 0.04 (4)**
	Durasingh et al. (2000) [6]	1.00	0.47		0.35		0.21
	Reed et al. (2000) [7]	1.00					0.21
	Johnson et al. (2004) [8]	1.00		2.14	0.77		0.45
	Lakshmanan et al. (2005) [9]	1.00		1.31			
	Baniecki et al. (2007) [10]	1.00	0.78	2.12			
Mefloquine[b]	Van Tyne et al. (2011) [2]	1.00	0.54	0.72	0.65		0.29
	Yuan et al. (2011) [11]	1.00	0.51	0.61		0.07	0.07
	Chugh et al. (2015) [12]	1.00	1.03	1.40	0.53		0.32
	Eastman et al. (2016) [4]	1.00	1.58	0.79		0.76	0.22
	Ross et al. (2018) [5]	1.00		1.33			
	**Mean ± SEM or range/2 (n)**	**1.00 ± 0.00 (10)**	**0.82 ± 0.17 (6)**	**1.30 ± 0.21 (8)**	**0.58 ± 0.09 (4)**	**0.42 ± 0.35 (2)**	**0.25 ± 0.04 (7)**
	Foote et al. (1990) [13]	1.10	1.00		16.00		2.89
	Durasingh et al. (2000) [6]	1.04	1.00				
	Fidock et al. (2000) [14]	0.53	1.00	5.45			
	Mehlotra et al. (2001) [15]	0.38	1.00	8.00	20.00		3.62
	Mu et al. (2003) [16]	0.57	1.00	11.90			6.50
	Johnson et al. (2004) [8]	0.59	1.00	6.72			4.83
	Lakshmanan et al. (2005) [9]	1.11	1.00	12.77			
	Baniecki et al. (2007) [10]	1.33	1.00	13.16			
	Sá et al. (2009) [17]		1.00	13.59		11.77	8.11
Chloroquine[b]	Mu et al. (2010) [18]	0.58	1.00	8.37		11.84	
	Valderramos et al. (2010) [1]	0.97	1.00	8.87			6.06
	Sanchez et al. (2011) [19]		1.00	7.53		5.99	4.18
	Van Tyne et al. (2011) [2]	0.91	1.00	7.51	8.84		5.74
	Griffin et al. (2012) [20]		1.00	11.60	12.00		7.87
	Chugh et al. (2015) [12]	1.00	1.00	20.10	23.75		7.32
	Reiling et al. (2015) [21]	0.51	1.00	3.60			
	Ross et al. (2018) [5]	0.94	1.00	16.30			
	This study (S4 Text)	0.68	1.00	7.16			
	**Mean ± SEM (n)**	**0.82 ± 0.07 (15)**	**1.00 ± 0.00 (18)**	**10.16 ± 1.08 (16)**	**16.12 ± 2.68 (5)**	**9.87 ± 1.94 (3)**	**5.71 ± 0.57 (10)**
	Durasingh et al. (2000) [6]	1.00	1.21				
	Chen et al. (2003) [22]	1.00			3.11		1.89
	Mu et al. (2003) [16]	1.00	2.50	3.95			1.54
	Johnson et al. (2004) [8]	1.00		6.47			1.78
	Lakshmanan et al. (2005) [9]	1.00		4.93			
Quinine[b]	Mu et al. (2010) [18]	1.00	3.73	4.09		3.22	
	Valderramos et al. (2010) [1]	1.00		5.25			3.07
	Van Tyne et al. (2011) [2]	1.00	0.83	3.21	3.17		1.58
	Ross et al. (2018) [5]	1.00		6.25			
	This study (S4 Text)	1.00	2.76	4.68			
	**Mean ± SEM or range/2 (n)**	**1.00 ± 0.00 (10)**	**2.20 ± 0.53 (5)**	**4.86 ± 0.40 (8)**	**3.14 ± 0.03 (2)**	**3.22 (1)**	**1.97 ± 0.28 (5)**

[a]The PfCRT isoform expressed by the 3D7 and HB3/GC03 strains is $PfCRT^{3D7}$. The other parasite strains express the PfCRT isoform indicated by their name, e.g., the Dd2 strain expresses $PfCRT^{Dd2}$ (see S2 Table).

[b]The *in vitro* drug resistance indices for lumefantrine, mefloquine, and quinine were calculated by dividing the IC_{50} value measured in each strain by the IC_{50} value determined in the same study for 3D7 parasites. However, the *in vitro* resistance indices for chloroquine were calculated by dividing the IC_{50} value measured in each strain by the IC_{50} value determined in the same study for HB3 or GC03 parasites because in some of these studies, the chloroquine IC_{50} value for 3D7 parasites was not determined (i.e. the HB3 or GC03 strains were used as the chloroquine-sensitive control strains in these studies).
