# Supplementary material for: Mechanistic basis for multidrug resistance and collateral drug sensitivity conferred to the malaria parasite by polymorphisms in PfMDR1 and PfCRT
Source: PLoS Biol. 2022 May 4;20(5):e3001616. doi: 10.1371/journal.pbio.3001616 (PMC9067703; doi:10.1371/journal.pbio.3001616)
Supplement: S4 Table — PfMDR1, Plasmodium falciparum multidrug resistance protein 1. (PDF) [file pbio.3001616.s014.pdf]

S4 Table: Generation of PfMDR1 coding sequences for expression in *Xenopus* oocytes.

Template	Mutation or tag	Forward primer 5'→3'[a]	Reverse primer 5'→3'[a]	Resulting construct
PfMDR1^{NYSND}	N86Y	CATGtatCTGGGGGA	CCAGataCATGTTCTTCAG	PfMDR1^{YYSND}
PfMDR1^{NYSND}	N86F	CATGttcCTGGGGGATGATAT	CCAGgaaCATGTTCTTCAGAATC	PfMDR1^{FYSND}
PfMDR1^{NYSND}	N86Y,<br>Y184F	CATGtatCTGGGGGA<br>CTGGGGCTTtttATTTGGTCG	CATGtatCTGGGGGA<br>CAAATaaaAAGCCCCAGGAACG	PfMDR1^{YFSND}
PfMDR1^{NYSND}	Y184F,<br>N1042D	CTGGGGCTTtttATTTGGTCG<br>TGTTTATTgatAGTTTCGCGTAC	CAAATaaaAAGCCCCAGGAACG<br>GTACGCGAAACTatcAATAAACA	PfMDR1^{NFSDD}
PfMDR1^{NFSDD}	S1034C,<br>D1246Y	GGTTTCtgtCAATCGGCGCAA<br>CCTGAGGtatCTTAGAAA	CGATTGacaGAAACCCCATAG<br>TTTCTAAGataCCTCAGG	PfMDR1^{NFCDY}
PfMDR1^{NYSND}	E588Q,<br>E1337Q	CTAATTCTTGACcagGCAACAAG<br>GCTTCTGGACcagGCTACAAG	CTTGTTGCctgGTCAAGAATTAG<br>CTTGTAGCctgGTCCAGAAGC	inactive PfMDR1^{NYSND}
PfMDR1^{NYSND}	2xHA	CGA**GGATCC***GCCGCCACC*ATGGGCAAA	CGA**GAATTC**TTA*tgcatagtcaggaacatcataaggatatgccactgctgcatagtcaggaacatcatatggata*GGCCACGGCTTTTGCCAGTTTCACATACTTTTTG	PfMDR1^{NYSND}-2xHA

[a]Mutated codons are shown in lowercase, restriction enzyme sites are shown in bold, the synthetic Kozak sequence is shown in italicized uppercase, and the coding sequence of the 2x hemagglutinin (HA)-tag is shown in italicized lowercase.
