# Supplementary material for: Mechanistic basis for multidrug resistance and collateral drug sensitivity conferred to the malaria parasite by polymorphisms in PfMDR1 and PfCRT
Source: PLoS Biol. 2022 May 4;20(5):e3001616. doi: 10.1371/journal.pbio.3001616 (PMC9067703; doi:10.1371/journal.pbio.3001616)
Supplement: S1 Text — PfMDR1, Plasmodium falciparum multidrug resistance protein 1. (PDF) [file pbio.3001616.s015.pdf]

S1 Text: Effects of N- or C-terminal epitope-tags on the expression and function of PfMDR1 in *Xenopus* oocytes.

The addition of a triple HA-tag to the N-terminus of PfMDR1^{NYSND} caused a modest reduction in the protein's ability to transport lumefantrine (S5 Fig). A possible explanation for this small decrease is that the addition of the triple HA-tag induces a conformational change in PfMDR1 that slightly hinders lumefantrine from accessing, or binding to, the substrate cavity. By contrast, the addition of either a double or a triple HA-tag to the C-terminus of PfMDR1 had a profound effect, almost completely abolishing protein expression and thus resulting in little or no lumefantrine transport (S5 Fig). This finding suggests that the C-terminus plays an important role in the correct folding and functional expression of PfMDR1; given the close proximity, perhaps the HA-tags interfere with the correct folding of NBD 2 — a region of the protein crucial for the function of the transporter. The misfolded protein is likely to be degraded by the oocyte, resulting in very low levels of functional protein at the surface of the oocyte. This scenario is consistent with the findings of a previous study that identified a C-terminal dileucine motif (located at the end of NBD 2) in human P-gp that is important for protein folding [1]. This dileucine motif is conserved in several MDR1 proteins, including PfMDR1. Thus, the addition of the HA-tags to the C-terminus of PfMDR1 may perturb the structure of this region, leading to the eventual degradation of the epitope-tagged protein.

Given that the N-terminal triple HA-tag had only a modest effect on protein expression, we used N-terminal triple HA-tagged versions of PfMDR1 in the immunofluorescence assays and the semiquantitative western blot analyses (S4–S5 Figs) to confirm the localization and expression levels of the PfMDR1 isoforms in *Xenopus* oocytes, respectively. Investigation of the orientation of PfMDR1 in the oocyte plasma membrane (S4 Fig) employed both N- and C-terminally HA-tagged versions of PfMDR1 (3xHA-PfMDR1^{NYSND} and PfMDR1^{NYSND}-3xHA). Although the expression level of PfMDR1^{NYSND}-3xHA is significantly lower than that of 3xHA-PfMDR1^{NYSND}, this should not affect the interpretation of the results because (1) the N- and C-termini are expected to be on the same side of the oocyte plasma membrane (noting that PfMDR1 has an even number of transmembrane domains) and (2) given that a fluorescent signal was not obtained with the 3xHA-PfMDR1^{NYSND}-expressing

oocytes, it stands to reason that a fluorescent signal would likewise be absent from the PfMDR1^{NYSND}-3xHA-expressing oocytes.

References

1. Loo TW, Bartlett MC, Clarke DM. The dileucine motif at the COOH terminus of human multidrug resistance P-glycoprotein is important for folding but not activity. J Biol Chem. 2005;280: 2522-2528.
