# Supplementary material for: Mechanistic basis for multidrug resistance and collateral drug sensitivity conferred to the malaria parasite by polymorphisms in PfMDR1 and PfCRT
Source: PLoS Biol. 2022 May 4;20(5):e3001616. doi: 10.1371/journal.pbio.3001616 (PMC9067703; doi:10.1371/journal.pbio.3001616)
Supplement: S2 Text — PfMDR1, Plasmodium falciparum multidrug resistance protein 1. (PDF) [file pbio.3001616.s016.pdf]

S2 Text: Direct measurement of the transport of fluorescent compounds via PfMDR1.

The radiolabeled drug transport assay was repurposed to measure the PfMDR1-mediated transport of compounds with inherent fluorescence. Three fluorescent compounds — methylene blue and quinacrine (the first synthetic antimalarials to be deployed), as well as rhodamine B (a fluorescent dye) — have previously been identified as substrates of human P-gp [1, 2]. We confirmed these findings by demonstrating that all three compounds were effluxed from oocytes expressing human P-gp (S1 Data), and we also showed that these compounds were substrates of PfMDR1 (Fig 2 and S1 Data). The low level of leakage from the negative control oocytes (non-expressing oocytes and those expressing PfNT1) was most likely due to simple diffusion of the neutral species of the compound.

References

1. Eytan GD, Regev R, Oren G, Hurwitz CD, Assaraf YG. Efficiency of P-glycoprotein-mediated exclusion of rhodamine dyes from multidrug-resistant cells is determined by their passive transmembrane movement rate. Eur J Biochem. 1997;15: 104-112.
2. Senarathna SM, Page-Sharp M, Crowe A. The interactions of P-glycoprotein with antimalarial drugs, including substrate affinity, inhibition and regulation. PloS one. 2016;11: e0152677.
