# Supplementary material for: Mechanistic basis for multidrug resistance and collateral drug sensitivity conferred to the malaria parasite by polymorphisms in PfMDR1 and PfCRT
Source: PLoS Biol. 2022 May 4;20(5):e3001616. doi: 10.1371/journal.pbio.3001616 (PMC9067703; doi:10.1371/journal.pbio.3001616)
Supplement: S3 Text — PfMDR1, Plasmodium falciparum multidrug resistance protein 1. (PDF) [file pbio.3001616.s017.pdf]

S3 Text: Roles of PfMDR1 in modulating the parasite's response to a range of antimalarial drugs.

PfMDR1 is involved in altering the response of the malaria parasite to a range of structurally diverse antimalarial drugs. The impacts of both amino acid mutations and *pfmdr1* copy number variation on drug resistance are discussed below in the context of our findings on the capacities of different field PfMDR1 isoforms to transport antimalarial drugs.

Mefloquine

The amplification of *pfmdr1* has been correlated with reduced parasite susceptibility to mefloquine (though this is not always the case [1]), whereas the N86Y, N1042D, and D1246Y mutations have been associated with increased sensitivity to the drug [2-12]. There does not appear to be an association between altered mefloquine susceptibility and Y184F or S1034C [13, 14]. Our datasets revealed that in the *Xenopus* oocyte system, the capacity for mefloquine transport via PfMDR1 isoforms decreases in the order: PfMDR1^{NYSND} > PfMDR1^{NFSDD} > PfMDR1^{FYSND} > PfMDR1^{YFSND} = PfMDR1^{YYSND} > PfMDR1^{NFCDY} (Fig 2b and S1 Data). The reduced mefloquine transport activities of the mutant PfMDR1 isoforms would lead to less of the drug entering and accumulating within the DV, and thus a greater proportion of mefloquine remaining in the cytosol. Indeed, several of these mutant isoforms have been linked to increases in the parasite's sensitivity to mefloquine [2-12]. For example, the introduction of the N86Y mutation into PfMDR1^{NYSND} — yielding PfMDR1^{YYSND} — caused a significant reduction in the protein's ability to transport mefloquine (Fig 2b, Fig 3b, and S1 Data), consistent with the linkage of this polymorphism to mefloquine-sensitive phenotypes in a recent genetic cross [10]. A smaller decrease in mefloquine transport is observed when the N86F mutation is introduced into PfMDR1^{NYSND} (yielding PfMDR1^{FYSND}; Fig 2b and S1 Data). This is in accordance with the findings of two independent studies that demonstrated that N86F was associated with mefloquine resistance *in vitro* [7, 11]. Furthermore, the impacts of N1042D and D1246Y are evident in the PfMDR1^{NFSDD} and PfMDR1^{NFCDY} isoforms, with both proteins having lower mefloquine transport activities than PfMDR1^{NYSND} (Fig 2b and S1 Data). This is consistent with previous studies that reported an increase in the *in vitro* mefloquine susceptibility of parasites expressing a *pfmdr1* allele containing one or both of these mutations [2, 4, 12].

Our findings are therefore consistent with a scenario in which mefloquine exerts its primary antimalarial activity in the cytosol (the cytosolic 80S ribosome has been reported as a target [15]) as this is the most parsimonious explanation for why a reduction in mefloquine transport into the DV would increase the killing effect of mefloquine (Fig 7). This model also provides a mechanistic explanation for why the amplification of wild-type *pfmdr1*, which results in the overexpression of $PfMDR1^{NYSND}$ [6, 9, 14, 16, 17], results in mefloquine resistance. Higher levels of $PfMDR1^{NYSND}$ in the DV membrane will significantly increase the rate of mefloquine transport from the cytosol into the DV, resulting in the sequestration of the drug away from its cytosolic target(s). Our observations also indicate that the targets previously proposed for mefloquine inside the DV (such as the detoxification of heme [18]) play secondary roles, or perhaps no role, in the antimalarial activity of mefloquine.

An analysis of the relationship between the rates of mefloquine transport via the six field PfMDR1 isoforms and the corresponding *in vitro* parasite responses to mefloquine revealed a strong positive correlation (Fig 5b and S3 Table; Pearson correlation coefficient = 0.97; *P* = 0.006). That is, PfMDR1 isoforms with relatively high capacities for mefloquine transport tended to be present in the parasites that had relatively high mefloquine IC_{50}s (and thus increased resistance to the drug), whereas those isoforms with low capacities for mefloquine transport tended to be present in the parasites with low mefloquine IC_{50}s (and thus increased sensitivity to the drug). However, the data point for Dd2 is an outlier to this trend and we found that the correlation was significantly improved by its exclusion. The Dd2 strain typically harbors 2–4 copies of *pfmdr1* [3, 14, 19], whereas the other parasite strains included in our analysis possess a single copy of the gene. Thus, the variations in *pfmdr1* copy number between the different Dd2 strains is likely to underlie, at least in part, the significant variations in the level of mefloquine resistance reported for Dd2 parasites in different studies (the mefloquine resistance indices for Dd2 strains range from 0.61 to 2.12; S3 Table). The presence of multiple copies of *pfmdr1* is known to result in the overexpression of PfMDR1 and the degree of overexpression correlates positively with the *pfmdr1* copy number [11, 20-23]. Furthermore, although the $PfMDR1^{YYSND}$ and $PfMDR1^{FYSND}$ isoforms have reduced capacities for mefloquine transport, these deficiencies are likely to be increasingly counterbalanced by higher levels of the transporter at the DV membrane as the *pfmdr1* copy number increases from 2 to 4. Hence, when considered in the context of our datasets as well as the mechanistic model we are proposing for the role of PfMDR1 in

mefloquine resistance (Fig 7), it is perhaps not surprising that a range of mefloquine responses are observed between different Dd2 strains. Overall, the strong correlation between the rate of mefloquine transport via a given PfMDR1 isoform and the *in vitro* mefloquine response of the corresponding parasite strain indicates that PfMDR1 contributes to mefloquine resistance by decreasing the cytosolic concentration of the drug, which in turn is consistent with mefloquine having its primary target in the cytosol [15].

Lumefantrine

Parasites with reduced susceptibility to mefloquine also tend to display a decrease in sensitivity to lumefantrine [22, 24-27]. For example, polymorphisms such as N86Y and D1246Y, which have been linked with increased mefloquine sensitivity, are also associated with increased susceptibility to lumefantrine [5, 14, 28-36]. Moreover, N86 is frequently selected over N86Y in response to artemether-lumefantrine treatment [28-35, 37]. As might be expected from these shared trends, we found that the order in which lumefantrine transport activity decreased across the six field isoforms was similar to that observed for mefloquine: PfMDR1^{NYSND} > PfMDR1^{NFSDD} = PfMDR1^{YYSND} > PfMDR1^{FYSND} = PfMDR1^{YFSND} > PfMDR1^{NFCDY} (Fig 2a and S1 Data). However, it is worth noting that whilst PfMDR1^{NFCDY} exhibits the lowest transport activity in both datasets, it is a particularly poor transporter of mefloquine. The other notable point of difference is PfMDR1^{YYSND}; it ranks equal second for lumefantrine transport activity but equal fourth for mefloquine transport activity.

Given the association between mutations in PfMDR1 and increases in the parasite’s sensitivity to lumefantrine, our demonstration of reduced lumefantrine transport via the mutant isoforms indicates that it is the reduction in the sequestration of lumefantrine within the DV, and its concomitant accumulation with the cytosol, that underpins the parasite’s increased sensitivity to lumefantrine. Hence, our results support a scenario in which lumefantrine exerts its primary antimalarial effect outside of the DV (Fig 7).

As was the case for mefloquine, we observed a strong positive correlation between the rates of lumefantrine transport via the PfMDR1 field isoforms and the *in vitro* lumefantrine responses of parasites carrying these isoforms (Fig 5a and S3 Table; Pearson correlation coefficient = 0.76, *P* =

0.078). This relationship indicates that PfMDR1's contribution to lumefantrine resistance is similar to its role in mefloquine resistance; overexpression of PfMDR1^{NYSND} will increase the sequestration of lumefantrine within the DV, whereas the reduced transport capacities of the mutant isoforms will increase the concentration of lumefantrine within the cytosol. The access of lumefantrine to its primary antimalarial target is evidently decreased by the former and increased by the latter (Fig 7). Further mechanistic insights into the roles played by PfMDR1 mutations in the parasite's response to lumefantrine may be gained by determining the drug transport activities of other PfMDR1 field isoforms (e.g. the NFSND, NYSNY, YYSNY, and YFSNY variants).

Chloroquine

Mutations in PfCRT are the primary determinant of chloroquine resistance in *P. falciparum*. However, polymorphisms in *pfmdr1* can increase the level of chloroquine resistance exhibited by parasites carrying mutant isoforms of PfCRT. Our datasets show that the capacity of PfMDR1 for chloroquine transport decreases in the order: PfMDR1^{NYSND} > PfMDR1^{NFSDD} > PfMDR1^{YFSND} = PfMDR1^{YYSND} = PfMDR1^{FYSND} > PfMDR1^{NFCDY} (Fig 2c and S1 Data). Hence, all of the mutant field isoforms have lower capacities for chloroquine transport than wild-type PfMDR1. Points of similarity between the chloroquine, mefloquine, and lumefantrine profiles include PfMDR1^{NFSDD} (which ranks second — or equal second — in all three datasets) and PfMDR1^{NFCDY} (which exhibits the most dramatic reduction in drug transport activity relative to wild-type PfMDR1). However, overall the chloroquine dataset shares more similarities with the profile obtained for piperaquine (see below) than it does with the patterns observed for mefloquine and lumefantrine.

A previous attempt by Sanchez *et al*. [38] to express PfMDR1 in *Xenopus* oocytes indicated that PfMDR1^{NYSND} was capable of chloroquine transport, but no transport was detected via PfMDR1^{NFSDD}, PfMDR1^{YYSND}, or PfMDR1^{NFCDY}. Since the rate of PfMDR1^{NYSND}-mediated chloroquine transport detected by Sanchez *et al*. [38] (~15 fmol/oocyte/h) is ~35-fold lower than that obtained in our study (531 ± 18 fmol/oocyte/h), it is likely that the chloroquine transport activities of the mutant PfMDR1 isoforms (which we have shown to be lower than that of PfMDR1^{NYSND}) were below the limit of detection in the assays employed by Sanchez *et al*. [38]. Nevertheless, both datasets demonstrate

that the addition of mutations to PfMDR1^{NYSND} results in a decrease in the ability of the protein to transport chloroquine.

Our findings reveal that the PfMDR1 isoforms typically found in chloroquine-sensitive parasites (i.e., PfMDR1^{NYSND} and PfMDR1^{NFSDD}) exhibit the greatest capacities for chloroquine transport, whereas those from chloroquine-resistant parasites (i.e., PfMDR1^{FYSND}, PfMDR1^{YFSND}, PfMDR1^{YYSND}, and PfMDR1^{NFCDY}) exhibit reduced capacities for chloroquine transport (Fig 2 and Fig 3). The decreased capacities of the mutant field PfMDR1 isoforms for chloroquine transport would result in less of the drug entering and accumulating within the DV, which is where chloroquine exerts its primary antimalarial effect by inhibiting the detoxification of heme [39-42]. Depending on the rate of chloroquine efflux mediated by the PfCRT isoform present at the DV membrane, as well as the rate of chloroquine entry into the DV via simple diffusion (which is dependent on the concentration of chloroquine in the parasite cytosol and, in turn, the extracellular concentration of chloroquine), the reduction in chloroquine import via PfMDR1 could contribute to a net decrease in the DV accumulation of chloroquine. In this scenario, the combination of a mutant PfCRT isoform (that mediates chloroquine efflux from the DV) and a mutant PfMDR1 isoform (with a decreased capacity for chloroquine transport into the DV) could achieve a lower DV concentration of chloroquine than would be achieved by the transport activity of PfCRT alone. On the other hand, we would anticipate that the sequestration of chloroquine within the DV would be at its highest when wild-type PfCRT (PfCRT^{3D7}, which lacks significant chloroquine transport activity) is paired with the overexpression of wild-type PfMDR1 (PfMDR1^{NYSND}), as this is likely to result in a very high rate of chloroquine import into the DV and little or no efflux of the drug back out into the cytosol. Further support for this mechanistic model (Fig 7) comes from *in vitro* studies that have implicated the deamplification of *pfmdr1* in high-level chloroquine resistance [43, 44]; a return to ‘normal’ expression levels of PfMDR1 would cause a decrease in the rate of chloroquine transport into the DV.

The chloroquine resistance indices (S3 Table) indicate that parasites expressing a PfMDR1 isoform containing N86Y as well as a mutant isoform of PfCRT exhibit high levels of chloroquine resistance [8, 33, 36, 45]. By contrast, 3D7 and HB3 parasites (which carry PfCRT^{3D7} and express PfMDR1^{NYSND} or PfMDR1^{NFSDD}, respectively) exhibit high levels of chloroquine susceptibility, with the HB3 strains

appearing to be slightly less sensitive to chloroquine ($P < 0.05$). The latter observation is consistent with the reduced capacity of PfMDR1^{NFSDD} for chloroquine transport into the DV, which could be resulting in a lower DV concentration of the drug and thus a reduction in the parasite's sensitivity to chloroquine. That said, other genetic factors, aside from polymorphisms in *pfcrt* and *pfmdr1*, are thought to exert modest effects on the parasite's susceptibility to chloroquine [46, 47], and one or more of these factors could be responsible for the difference between 3D7 and HB3. Only one of the commonly studied strains (7G8) expresses PfMDR1^{NFCDY} — the PfMDR1 isoform with the lowest rate of chloroquine transport — and it should be noted that the mutant PfCRT isoform carried by these parasites (PfCRT^{7G8}) has the lowest chloroquine transport activity of the mutant field PfCRT isoforms measured to date. In a previous study, we identified a strong positive correlation between PfCRT chloroquine transport activity and the *in vitro* parasite response to the drug [48], but the 7G8 strain was an outlier to this trend because it exhibited a greater level of chloroquine resistance than what would be predicted given the low capacity of PfCRT^{7G8} for chloroquine transport [48]. Given these previous findings, the relatively moderate level of chloroquine resistance exhibited by parasites carrying PfMDR1^{NFCDY} is likely to be a reflection of the aforementioned interplay between the rate of chloroquine import via PfMDR1 and the rate of chloroquine efflux via PfCRT, with the transport activity of PfCRT having the greater impact on the concentration of chloroquine within the DV (mutations in PfCRT are the primary determinant of chloroquine resistance) and thus the parasite's susceptibility to the drug.

An analysis of the relationship between the rates of chloroquine transport via PfMDR1 and the *in vitro* response to the drug by parasites expressing the corresponding PfMDR1 isoform revealed a negative correlation (Fig 5c and S3 Table). As foreshadowed above, the data point for 7G8 lay outside of this trend; the chloroquine resistance index of the 7G8 parasite was lower than what might be expected from the low rate of chloroquine transport mediated by PfMDR1^{NFCDY}. However, this outlier is readily explained by the fact that PfCRT^{7G8} has a relatively low capacity for chloroquine transport and thus confers a lower level of chloroquine resistance than the other PfCRT isoforms [48]. Exclusion of the 7G8 data point strengthens the negative correlation between PfMDR1 chloroquine transport capacity and the *in vitro* chloroquine resistance index (Pearson correlation coefficient = -0.86; $P = 0.0597$). Together, these findings suggest that PfMDR1^{NFCDY} contributes to the chloroquine resistance

phenotype to a greater extent than other PfMDR1 isoforms as a consequence of its very low capacity for chloroquine transport.

Taken together, our datasets for chloroquine transport via PfCRT and PfMDR1 are also consistent with a previously reported relationship between total chloroquine accumulation, chloroquine resistance, and polymorphisms in *pfcrt* and *pfmdr1* [49]. The chloroquine responses of the progeny from a GB4 x 7G8 genetic cross were determined, and differences in the accumulation of chloroquine between strains were estimated by measuring the total accumulation of label in parasite-infected red blood cells that had been incubated with radiolabelled chloroquine. Parasites harbouring $PfMDR1^{NFCDY}$ (the '7G8' isoform of PfMDR1) exhibited greater resistance to chloroquine than their counterparts harbouring $PfMDR1^{YFSND}$ (the isoform carried by GB4 parasites), and were also found to accumulate less of the drug. However, for the reasons set out below, significant caution should be exercised when drawing comparisons between the rates of drug transport we have measured for PfMDR1 and PfCRT in the oocyte system and measurements of total drug accumulation made with parasite-infected red blood cells (e.g., [5, 49]).

First, the measurements of total drug accumulation do not reveal how much drug has accumulated within the parasite DV versus in the parasite cytosol, nor how these two concentrations vary between transfectant lines expressing different isoforms of either PfMDR1 or PfCRT. This lack of sub-cellular resolution is somewhat limiting given that our datasets, and the resulting mechanistic models, link the acquisition of multidrug resistance and collateral drug sensitivity to altered drug distribution between these two compartments.

Secondly, and perhaps more importantly, because accumulation assays typically entail only a single measurement, they will not have detected all of the differences between parasite strains/lines if one or more of them reached a 'steady state' before the others. It should also be noted that the extracellular drug concentration varies considerably in these assays as the incubation progresses. For example, significant decreases in the extracellular chloroquine concentration occur in suspensions of chloroquine-sensitive parasites due to the very high sequestration of the drug within the DV, whereas much smaller decreases occur in suspensions of chloroquine-resistant parasites. A full determination

of whether parasite strains/lines exhibit differences in total drug accumulation would require a time-course of measurements, as well as repeating the time-course in the presence of different extracellular concentrations of the drug. An analysis of the resulting dataset would yield the initial rate of total drug accumulation in the parasitized red blood cell, which would be — at least in part — a reflection of the net rate of drug flux across the DV membrane. That is, the total rate of drug transport into the DV (via diffusion, PfMDR1, and any other route of influx) minus the total rate of drug export (via PfCRT and any other route of efflux). Such an approach is more likely to detect the full spectrum of differences in the accumulation of drugs between parasite lines/strains.

Thirdly, the genetic background — and in particular the isoform of PfCRT expressed — appears to have a major impact on the ability of the assay to detect changes in total drug accumulation between parasites carrying different *pfmdr1* polymorphisms. For example, in the study of the GB4 x 7G8 progeny, polymorphisms in *pfmdr1* appeared to have little or no impact on the total accumulation of chloroquine in parasites carrying $PfCRT^{GB4}$ [49], and a similar result was observed in a study of transgenic lines that carried different *pfmdr1* polymorphisms (and which all expressed $PfCRT^{GB4}$) [5]. $PfCRT^{GB4}$ has a very high capacity for transporting chloroquine out of the DV [48] and is thus likely to appear as the key determinant, if not the only determinant, of total chloroquine accumulation across parasites carrying different *pfmdr1* polymorphisms — especially if only a single measurement is made (see above). By contrast, performing the assays with parasites carrying a low-capacity, high-affinity transporter of chloroquine (e.g. $PfCRT^{7G8}$) appears to facilitate detection of the effects of *pfmdr* polymorphisms on total chloroquine accumulation.

Amodiaquine

The mode of action and trends in the parasite’s response to amodiaquine are very similar to those observed for chloroquine. For example, both drugs exert their primary antimalarial effect in the DV by interfering with the detoxification of heme [50], and the mutations in PfCRT that confer chloroquine resistance also decrease the parasite’s susceptibility to amodiaquine [5, 31-33, 36, 49, 51-54]. Likewise, the PfMDR1 mutations that are associated with decreased susceptibility to chloroquine — N86Y and D1246Y — have also been shown to decrease the parasite’s susceptibility to amodiaquine [55]. Our datasets reveal that the amodiaquine transport activities of the six PfMDR1 isoforms

decrease in the order: PfMDR1^{NYSND} > PfMDR1^{YYSND} = PfMDR1^{YFSND} > PfMDR1^{FYSND} > PfMDR1^{NFSDD} > PfMDR1^{NFCDY} (Fig 2e and S1 Data). Whilst there are some similarities between the chloroquine and amodiaquine datasets, the points of difference include: (1) PfMDR1^{NFSDD} (it ranks fifth for amodiaquine transport activity, but is second (or equal second) in the chloroquine, lumefantrine, and mefloquine datasets), (2) PfMDR1^{YYSND} (it ranks equal second for amodiaquine and lumefantrine transport activities, but is equal third and equal fourth in the chloroquine and mefloquine datasets, respectively), and (3) PfMDR1^{NFCDY} (it ranks sixth in all four datasets, but is a particularly poor transporter of amodiaquine and mefloquine relative to wild-type PfMDR1).

These variations aside, our findings indicate that PfMDR1's contribution to amodiaquine resistance is likely to be similar to its role in chloroquine resistance (Fig 7), whereby decreases in the PfMDR1-mediated import of amodiaquine could result in a lower DV concentration of the drug and thus a reduction in the parasite's susceptibility to amodiaquine. Characterization of the effects of the PfMDR1 mutations that have been reported in areas following amodiaquine treatment failure (such as PfMDR1^{YYSNY}) will provide further insights into the contribution of PfMDR1 to amodiaquine resistance.

Piperaquine

Piperaquine resistance in *P. falciparum* is a multifactorial phenomenon, with polymorphisms in *pfcrt* [56-59], *pfmdr1* [5, 60-63], and several other genes [62-65] appearing to influence the parasite's response to the drug [66, 67]. Deamplification of a region of chromosome 5 that includes *pfmdr1* was detected following the *in vitro* pressure of a Dd2 strain with piperaquine [61]. Furthermore, there has been a reduction in the prevalence of multicopy *pfmdr1* since the deployment of the dihydroartemisinin + piperaquine combination therapy [62, 63, 68, 69], and one study reported a modest inverse association between *pfmdr1* copy number and parasite susceptibility to piperaquine [60]. That said, the deamplification of *pfmdr1* in field isolates may also be due to the withdrawal of mefloquine, a drug that selects for *pfmdr1* amplification [6, 70]. Together, these studies suggest that the parasite's sensitivity to piperaquine is influenced by the level of PfMDR1 present in the DV membrane. In addition, novel mutant field isoforms of PfMDR1 have been shown to modulate the parasite's response to piperaquine [5].

We found that the capacity for piperaquine transport amongst the six field isoforms decreased in the order: PfMDR1^{NYSND} > PfMDR1^{NFSDD} = PfMDR1^{YYSND} > PfMDR1^{FYSND} = PfMDR1^{YFSND} > PfMDR1^{NFCDY} (Fig 2f and S1 Data). Again, although the chloroquine and piperaquine datasets are quite similar, the points of difference include: (1) PfMDR1^{YYSND} (it ranks equal second in the piperaquine, amodiaquine, and lumefantrine datasets, but is equal third and equal fourth in the chloroquine and mefloquine datasets, respectively) and (2) PfMDR1^{YFSND} (it ranks equal fourth in the piperaquine, lumefantrine, mefloquine datasets, but second (or equal second) in the chloroquine and amodiaquine datasets, respectively).

Given that several lines of evidence indicate that piperaquine exerts its primary antimalarial effects within the DV [62-64], our finding that mutant isoforms of PfMDR1 exhibit reduced piperaquine transport activities relative to the wild-type protein suggest that the mechanistic model we are proposing for the role of PfMDR1 in chloroquine resistance may also apply to piperaquine (Fig 7). That is, a reduction in the PfMDR1-mediated transport of piperaquine into the DV may lead to a lower concentration of the drug at its primary site of action, and thus a decrease in the parasite's sensitivity to piperaquine. More detailed mechanistic insights into PfMDR1's contribution to piperaquine resistance may be gained by measuring the drug transport activities of the novel field isoforms that have been implicated in the parasite's piperaquine response.

Methylene blue

There has been some interest in deploying methylene blue as a partner drug in triple combination therapies [71]. Methylene blue has been shown to inhibit hemozoin formation [72-74] and to target the parasite's glutathione reductase [75]. Chloroquine resistance-conferring mutations in PfCRT have been shown to decrease the parasite's susceptibility to methylene blue, and a mutant field isoform of PfCRT (PfCRT^{Dd2}) has been shown to transport the drug (whereas the wild-type transporter lacked this activity) [76]. A recent study detected a tentative association between PfMDR1^{YFSND} and a slight reduction in the parasite's susceptibility to the drug [77]. This study aside, little is understood about the effects of *pfmdr1* polymorphisms on the parasite's response to methylene blue.

Our measurements reveal that the methylene blue transport activities of the six field PfMDR1 isoforms decrease in the order: PfMDR1^{NYSND} > PfMDR1^{NFSDD} > PfMDR1^{YYSND} > PfMDR1^{FYSND} = PfMDR1^{YFSND} > PfMDR1^{NFCDY} (Fig 2h and S1 Data). Hence, the methylene blue dataset most closely resembles those of lumefantrine and quinacrine.

Although much remains to be confirmed in regard to the identity and location of methylene blue's primary antimalarial target(s) and the genetic determinants of resistance, the datasets we have presented here and elsewhere [76] are consistent with the mechanistic model we are proposing for chloroquine (Fig 7). The reduced capacities of the mutant PfMDR1 isoforms for the transport of methylene blue could lead to a decrease in its sequestration within the DV. Hence, the pairing of a PfCRT isoform that mediates methylene blue efflux from the DV (e.g. PfCRT^{Dd2}) with one of these mutant PfMDR1 isoforms could result in a significant reduction in the DV concentration of methylene blue. This change in drug distribution is likely to confer resistance to methylene blue if the DV is the drug's primary site of action.

Dihydroartemisinin

Polymorphisms in *pfmdr1* that are associated with increased parasite susceptibility to mefloquine, lumefantrine, and quinine can also sensitize parasites to artemisinin or dihydroartemisinin [2, 4, 8, 78, 79]. For example, genetic modification of parasites expressing chloroquine resistance-conferring isoforms of PfCRT revealed that PfMDR1^{NYSND} and PfMDR1^{NFSND} cause a decrease in the parasite's susceptibility to dihydroartemisinin relative to parasites expressing either the PfMDR1^{YYSND} or PfMDR1^{YFSND} isoforms [5, 14]. Our datasets reveal that the dihydroartemisinin transport activities of the six PfMDR1 field isoforms decrease in the order: PfMDR1^{NYSND} > PfMDR1^{NFSDD} > PfMDR1^{YFSND} > PfMDR1^{FYSND} = PfMDR1^{YYSND} > PfMDR1^{NFCDY} (Fig 2g and S1 Data). The dihydroartemisinin dataset is therefore reminiscent of those observed for mefloquine, quinine, and quinidine (a stereoisomer of quinine) in that PfMDR1^{YYSND} has a substantially lower transport activity than PfMDR1^{NFSDD}. Moreover, in all four datasets the transport activity of PfMDR1^{YYSND} is either below, or on par with, PfMDR1^{FYSND} and PfMDR1^{YFSND}.

Given that the mutant PfMDR1 isoforms have reduced levels of dihydroartemisinin transport activity relative to the wild-type protein, as well as the observation that parasites expressing these isoforms tend to be more susceptible to the drug, it appears that PfMDR1's contribution to dihydroartemisinin resistance may be similar to its role in both mefloquine and lumefantrine resistance (Fig 7). The reduced rate of dihydroartemisinin transport into the DV of parasites expressing a mutant PfMDR1 isoform could result in a higher concentration of the drug in the cytosol, and thus increased activity against its primary target(s).

References

1. Nkhoma SC, Ahmed AOA, Zaman S, Porier D, Baker Z, Stedman TT. Dissection of haplotype-specific drug response phenotypes in multiclonal malaria isolates. Int J Parasitol. 2021;15: 152-161.

2. Reed MB, Saliba KJ, Caruana SR, Kirk K, Cowman AF. Pgh1 modulates sensitivity and resistance to multiple antimalarials in *Plasmodium falciparum*. Nature. 2000;403: 906-909.

3. Reiling SJ, Rohrbach P. Monitoring PfMDR1 transport in *Plasmodium falciparum*. Malar J. 2015;14: 270.

4. Sidhu AB, Valderramos SG, Fidock DA. *pfmdr1* mutations contribute to quinine resistance and enhance mefloquine and artemisinin sensitivity in *Plasmodium falciparum*. Mol Microbiol. 2005;57: 913-926.

5. Veiga MI, Dhingra SK, Henrich PP, Straimer J, Gnadig N, Uhlemann AC, et al. Globally prevalent PfMDR1 mutations modulate *Plasmodium falciparum* susceptibility to artemisinin-based combination therapies. Nat Commun. 2016;7: 11553.

6. Price RN, Uhlemann AC, Brockman A, McGready R, Ashley E, Phaipun L, et al. Mefloquine resistance in *Plasmodium falciparum* and increased *pfmdr1* gene copy number. Lancet (London, England). 2004;364: 438-447.

7. Peel SA, Bright P, Yount B, Handy J, Baric RS. A strong association between mefloquine and halofantrine resistance and amplification, overexpression, and mutation in the P-glycoprotein gene homolog (*pfmdr*) of *Plasmodium falciparum in vitro*. Am J Trop Med Hyg. 1994;51: 648-658.

8. Duraisingh MT, Jones P, Sambou I, von Seidlein L, Pinder M, Warhurst DC. The tyrosine-86 allele of the *pfmdr1* gene of *Plasmodium falciparum* is associated with increased sensitivity to the anti-malarials mefloquine and artemisinin. Mol Biochem Parasitol. 2000;108: 13-23.

9. Price RN, Cassar C, Brockman A, Duraisingh M, van Vugt M, White NJ, et al. The *pfmdr1* gene is associated with a multidrug-resistant phenotype in *Plasmodium falciparum* from the western border of Thailand. Antimicrob Agents Chemother. 1999;43: 2943-2949.

10. Windle ST, Lane KD, Gadalla NB, Liu A, Mu J, Caleon RL, et al. Evidence for linkage of *pfmdr1*, *pfcrt*, and *pfk13* polymorphisms to lumefantrine and mefloquine susceptibilities in a *Plasmodium falciparum* cross. Int J Parasitol. 2020;14: 208-217.

11. Cowman AF, Galatis D, Thompson JK. Selection for mefloquine resistance in *Plasmodium falciparum* is linked to amplification of the *pfmdr1* gene and cross-resistance to halofantrine and quinine. Proc Natl Acad Sci USA. 1994;91: 1143-1147.

12. Duraisingh MT, Cowman AF. Contribution of the *pfmdr1* gene to antimalarial drug-resistance. Acta Trop. 2005;94: 181-190.

13. Wurtz N, Fall B, Pascual A, Fall M, Baret E, Camara C, et al. Role of *Pfmdr1* in *in vitro Plasmodium falciparum* susceptibility to chloroquine, quinine, monodesethylamodiaquine, mefloquine, lumefantrine, and dihydroartemisinin. Antimicrob Agents Chemother. 2014;58: 7032-7040.

14. Calçada C, Silva M, Baptista V, Thathy V, Silva-Pedrosa R, Granja D, et al. Expansion of a specific *Plasmodium falciparum* PfMDR1 haplotype in Southeast Asia with increased substrate transport. mBio. 2020;11: e02093-02020.

15. Wong W, Bai XC, Sleebs BE, Triglia T, Brown A, Thompson JK, et al. Mefloquine targets the *Plasmodium falciparum* 80S ribosome to inhibit protein synthesis. Nat Microbiol. 2017;2: 17031.

16. Uhlemann A-C, McGready R, Ashley EA, Brockman A, Singhasivanon P, Krishna S, et al. Intrahost selection of *Plasmodium falciparum pfmdr1* alleles after antimalarial treatment on the northwestern border of Thailand. J Infect Dis. 2007;195: 134-141.

17. Chaijaroenkul W, Wisedpanichkij R, Na-Bangchang K. Monitoring of *in vitro* susceptibilities and molecular markers of resistance of *Plasmodium falciparum* isolates from Thai-Myanmar border to chloroquine, quinine, mefloquine and artesunate. Acta Tropica. 2010;113: 190-194.

18. Combrinck JM, Mabotha TE, Ncokazi KK, Ambele MA, Taylor D, Smith PJ, et al. Insights into the role of heme in the mechanism of action of antimalarials. ACS Chem Biol. 2013;8: 133-137.

19. Bohórquez EB, Juliano JJ, Kim HS, Meshnick SR. Mefloquine exposure induces cell cycle delay and reveals stage-specific expression of the *pfmdr1* gene. Antimicrob Agents Chemother. 2013;57: 833-839.

20. Cowman AF, Karcz S, Galatis D, Culvenor JG. A P-glycoprotein homologue of *Plasmodium falciparum* is localized on the digestive vacuole. J Cell Biol. 1991;113: 1033-1042.

21. Rohrbach P, Sanchez CP, Hayton K, Friedrich O, Patel J, Sidhu AB, et al. Genetic linkage of *pfmdr1* with food vacuolar solute import in *Plasmodium falciparum*. EMBO J. 2006;25: 3000-3011.

22. Sidhu ABS, Uhlemann A-C, Valderramos SG, Valderramos J-C, Krishna S, Fidock DA. Decreasing *pfmdr1* copy number in *Plasmodium falciparum* malaria heightens susceptibility to mefloquine, lumefantrine, halofantrine, quinine, and artemisinin. J Infect Dis. 2006;194: 528-535.

23. Elandaloussi LM, Lindt M, Collins M, Smith PJ. Analysis of P-glycoprotein expression in purified parasite plasma membrane and food vacuole from *Plasmodium falciparum*. Parasitol Res. 2006;99: 631-637.

24. Eastman RT, Khine P, Huang R, Thomas CJ, Su XZ. PfCRT and PfMDR1 modulate interactions of artemisinin derivatives and ion channel blockers. Sci Rep. 2016;6: 25379.

25. Van Tyne D, Park DJ, Schaffner SF, Neafsey DE, Angelino E, Cortese JF, et al. Identification and functional validation of the novel antimalarial resistance locus PF10_0355 in *Plasmodium falciparum*. PLoS Genet. 2011;7: e1001383.

26. Veiga MI, Ferreira PE, Jornhagen L, Malmberg M, Kone A, Schmidt BA, et al. Novel polymorphisms in *Plasmodium falciparum* ABC transporter genes are associated with major ACT antimalarial drug resistance. PLoS One. 2011;6: e20212.

27. Chavchich M, Gerena L, Peters J, Chen N, Cheng Q, Kyle DE. Role of *pfmdr1* amplification and expression in induction of resistance to artemisinin derivatives in *Plasmodium falciparum*. Antimicrob Agents Chemother. 2010;54: 2455-2464.

28. Sisowath C, Strömberg J, Mårtensson A, Msellem M, Obondo C, Björkman A, et al. *In vivo* selection of *Plasmodium falciparum pfmdr1* 86N coding alleles by artemether-lumefantrine (Coartem). J Infect Dis. 2005;191: 1014-1017.

29. Conrad MD, LeClair N, Arinaitwe E, Wanzira H, Kakuru A, Bigira V, et al. Comparative impacts over 5 years of artemisinin-based combination therapies on *Plasmodium falciparum* polymorphisms that modulate drug sensitivity in Ugandan children. J Infect Dis. 2014;210: 344-353.

30. Baraka V, Tinto H, Valea I, Fitzhenry R, Delgado-Ratto C, Mbonye MK, et al. *In vivo* selection of *Plasmodium falciparum* variants by artemether-lumefantrine and dihydroartemisinin-piperaquine in Burkina Faso. Antimicrob Agents Chemother. 2015;59: 734-737.

31. Otienoburu SD, Maïga-Ascofaré O, Schramm B, Jullien V, Jones JJ, Zolia YM, et al. Selection of *Plasmodium falciparum pfcrt* and *pfmdr1* polymorphisms after treatment with artesunate-amodiaquine fixed dose combination or artemether-lumefantrine in Liberia. Malar J. 2016;15: 452.

32. Sondo P, Derra K, Diallo Nakanabo S, Tarnagda Z, Kazienga A, Zampa O, et al. Artesunate-amodiaquine and artemether-lumefantrine therapies and selection of *Pfcrt* and *Pfmdr1* alleles in Nanoro, Burkina Faso. PLoS One. 2016;11: e0151565.

33. Eyase FL, Akala HM, Ingasia L, Cheruiyot A, Omondi A, Okudo C, et al. The role of *Pfmdr1* and *Pfcrt* in changing chloroquine, amodiaquine, mefloquine and lumefantrine susceptibility in western-Kenya *P. falciparum* samples during 2008-2011. PLoS One. 2013;8: e64299.

34. Venkatesan M, Gadalla NB, Stepniewska K, Dahal P, Nsanzabana C, Moriera C, et al. Polymorphisms in *Plasmodium falciparum* chloroquine resistance transporter and multidrug resistance 1 genes: parasite risk factors that affect treatment outcomes for *P. falciparum* malaria after artemether-lumefantrine and artesunate-amodiaquine. Am J Trop Med Hyg. 2014;91: 833-843.

35. Somé AF, Séré YY, Dokomajilar C, Zongo I, Rouamba N, Greenhouse B, et al. Selection of known *Plasmodium falciparum* resistance-mediating polymorphisms by artemether-lumefantrine and amodiaquine-sulfadoxine-pyrimethamine but not dihydroartemisinin-piperaquine in Burkina Faso. Antimicrob Agents Chemother. 2010;54: 1949-1954.

36. Nsobya SL, Kiggundu M, Nanyunja S, Joloba M, Greenhouse B, Rosenthal PJ. *In vitro* sensitivities of *Plasmodium falciparum* to different antimalarial drugs in Uganda. Antimicrob Agents Chemother. 2010;54: 1200-1206.

37. Sisowath C, Petersen I, Veiga MI, Martensson A, Premji Z, Bjorkman A, et al. *In vivo* selection of *Plasmodium falciparum* parasites carrying the chloroquine-susceptible *pfcrt* K76 allele after treatment with artemether-lumefantrine in Africa. J Infect Dis. 2009;199: 750-757.

38. Sanchez CP, Rotmann A, Stein WD, Lanzer M. Polymorphisms within PfMDR1 alter the substrate specificity for anti-malarial drugs in *Plasmodium falciparum*. Mol Microbiol. 2008;70: 786-798.

39. Sullivan DJ, Jr., Gluzman IY, Russell DG, Goldberg DE. On the molecular mechanism of chloroquine's antimalarial action. Proc Natl Acad Sci USA. 1996;93: 11865-11870.

40. Sullivan DJ, Jr., Matile H, Ridley RG, Goldberg DE. A common mechanism for blockade of heme polymerization by antimalarial quinolines. J Biol Chem. 1998;273: 31103-31107.

41. Egan TJ, Ross DC, Adams PA. Quinoline anti-malarial drugs inhibit spontaneous formation of beta-haematin (malaria pigment). FEBS Lett. 1994;352: 54-57.

42. Slater AF, Cerami A. Inhibition by chloroquine of a novel haem polymerase enzyme activity in malaria trophozoites. Nature. 1992;355: 167-169.

43. Barnes DA, Foote SJ, Galatis D, Kemp DJ, Cowman AF. Selection for high-level chloroquine resistance results in deamplification of the *pfmdr1* gene and increased sensitivity to mefloquine in *Plasmodium falciparum*. EMBO J. 1992;11: 3067-3075.

44. Griffin CE, Hoke JM, Samarakoon U, Duan J, Mu J, Ferdig MT, et al. Mutation in the *Plasmodium falciparum* CRT protein determines the stereospecific activity of antimalarial cinchona alkaloids. Antimicrob Agents Chemother. 2012;56: 5356-5364.

45. Foote SJ, Kyle DE, Martin RK, Oduola AM, Forsyth K, Kemp DJ, et al. Several alleles of the multidrug-resistance gene are closely linked to chloroquine resistance in *Plasmodium falciparum*. Nature. 1990;345: 255-258.

46. Raj DK, Mu J, Jiang H, Kabat J, Singh S, Sullivan M, et al. Disruption of a *Plasmodium falciparum* multidrug resistance-associated protein (PfMRP) alters its fitness and transport of antimalarial drugs and glutathione. J Biol Chem. 2009;284: 7687-7696.

47. Chen N, Russell B, Fowler E, Peters J, Cheng Q. Levels of chloroquine resistance in *Plasmodium falciparum* are determined by loci other than *pfcrt* and *pfmdr1*. J Infect Dis. 2002;185: 405-406.

48. Summers RL, Dave A, Dolstra TJ, Bellanca S, Marchetti RV, Nash MN, et al. Diverse mutational pathways converge on saturable chloroquine transport via the malaria parasite's chloroquine resistance transporter. Proc Natl Acad Sci USA. 2014;111: E1759-1767.

49. Sanchez CP, Mayer S, Nurhasanah A, Stein WD, Lanzer M. Genetic linkage analyses redefine the roles of PfCRT and PfMDR1 in drug accumulation and susceptibility in *Plasmodium falciparum*. Mol Microbiol. 2011;82: 865-878.

50. Fitzroy SM, Gildenhuys J, Olivier T, Tshililo NO, Kuter D, de Villiers KA. The effects of quinoline and non-quinoline inhibitors on the kinetics of lipid-mediated beta-hematin crystallization. Langmuir. 2017;33: 7529-7537.

51. Sá JM, Twu O, Hayton K, Reyes S, Fay MP, Ringwald P, et al. Geographic patterns of *Plasmodium falciparum* drug resistance distinguished by differential responses to amodiaquine and chloroquine. Proc Natl Acad Sci USA. 2009;106: 18883-18889.

52. Folarin OA, Bustamante C, Gbotosho GO, Sowunmi A, Zalis MG, Oduola AMJ, et al. *In vitro* amodiaquine resistance and its association with mutations in *pfcrt* and *pfmdr1* genes of *Plasmodium falciparum* isolates from Nigeria. Acta Trop. 2011;120: 224-230.

53. Danquah I, Coulibaly B, Meissner P, Petruschke I, Müller O, Mockenhaupt FP. Selection of *pfmdr1* and *pfcrt* alleles in amodiaquine treatment failure in north-western Burkina Faso. Acta Trop. 2010;114: 63-66.

54. Foguim FT, Bogreau H, Gendrot M, Mosnier J, Fonta I, Benoit N, et al. Prevalence of mutations in the *Plasmodium falciparum* chloroquine resistance transporter, PfCRT, and association with *ex vivo* susceptibility to common anti-malarial drugs against African *Plasmodium falciparum* isolates. Malar J. 2020;19: 201.

55. Holmgren G, Hamrin J, Svärd J, Mårtensson A, Gil JP, Björkman A. Selection of *pfmdr1* mutations after amodiaquine monotherapy and amodiaquine plus artemisinin combination therapy in East Africa. Infect Genet Evol. 2007;7: 562-569.

56. Duru V, Khim N, Leang R, Kim S, Domergue A, Kloeung N, et al. *Plasmodium falciparum* dihydroartemisinin-piperaquine failures in Cambodia are associated with mutant K13 parasites presenting high

survival rates in novel piperaquine *in vitro* assays: retrospective and prospective investigations. BMC Med. 2015;13: 305-315.

57. Agrawal S, Moser KA, Morton L, Cummings MP, Parihar A, Dwivedi A, et al. Association of a novel mutation in the *Plasmodium falciparum* chloroquine resistance transporter with decreased piperaquine sensitivity. J Infect Dis. 2017;216: 468-476.

58. Dhingra SK, Small-Saunders JL, Ménard D, Fidock DA. *Plasmodium falciparum* resistance to piperaquine driven by PfCRT. Lancet Infect Dis. 2019;19: 1168-1169.

59. Ross LS, Dhingra SK, Mok S, Yeo T, Wicht KJ, Kumpornsin K, et al. Emerging Southeast Asian PfCRT mutations confer *Plasmodium falciparum* resistance to the first-line antimalarial piperaquine. Nat Commun. 2018;9: 3314.

60. Veiga MI, Ferreira PE, Malmberg M, Jörnhagen L, Björkman A, Nosten F, et al. *pfmdr1* amplification is related to increased *Plasmodium falciparum* in vitro sensitivity to the bisquinoline piperaquine. Antimicrob Agents Chemother. 2012;56: 3615-3619.

61. Eastman RT, Dharia NV, Winzeler EA, Fidock DA. Piperaquine resistance is associated with a copy number variation on chromosome 5 in drug-pressured *Plasmodium falciparum* parasites. Antimicrob Agents Chemother. 2011;55: 3908-3916.

62. Amato R, Lim P, Miotto O, Amaratunga C, Dek D, Pearson RD, et al. Genetic markers associated with dihydroartemisinin-piperaquine failure in *Plasmodium falciparum* malaria in Cambodia: a genotype-phenotype association study. Lancet Infect Dis. 2017;17: 164-173.

63. Witkowski B, Duru V, Khim N, Ross LS, Saintpierre B, Beghain J, et al. A surrogate marker of piperaquine-resistant *Plasmodium falciparum* malaria: a phenotype-genotype association study. Lancet Infect Dis. 2017;17: 174-183.

64. Bopp S, Magistrado P, Wong W, Schaffner SF, Mukherjee A, Lim P, et al. Plasmepsin II-III copy number accounts for bimodal piperaquine resistance among Cambodian *Plasmodium falciparum*. Nature communications. 2018;9: 1769.

65. Mukherjee A, Gagnon D, Wirth DF, Richard D. Inactivation of plasmepsins 2 and 3 sensitizes *Plasmodium falciparum* to the antimalarial drug piperaquine. Antimicrob Agents Chemother. 2018;62.

66. Small-Saunders JL, Hagenah LM, Fidock DA. Turning the tide: targeting PfCRT to combat drug-resistant *P. falciparum*? Nat Rev Microbiol. 2020;18: 261-262.

67. Wicht KJ, Mok S, Fidock DA. Molecular mechanisms of drug resistance in *Plasmodium falciparum* malaria. Annu Rev Microbiol. 2020;74: 431-454.

68. Parobek CM, Parr JB, Brazeau NF, Lon C, Chaorattanakawee S, Gosi P, et al. Partner-drug resistance and population substructuring of artemisinin-resistant *Plasmodium falciparum* in Cambodia. Genome Biol Evol. 2017;9: 1673-1686.

69. Imwong M, Hien TT, Thuy-Nhien NT, Dondorp AM, White NJ. Spread of a single multidrug resistant malaria parasite lineage (PfPailin) to Vietnam. Lancet Infect Dis. 2017;17: 1022-1023.

70. Preechapornkul P, Imwong M, Chotivanich K, Pongtavornpinyo W, Dondorp AM, Day NP, et al. *Plasmodium falciparum pfmdr1* amplification, mefloquine resistance, and parasite fitness. Antimicrob Agents Chemother. 2009;53: 1509-1515.

71. Müller O, Lu G, Jahn A, Mockenhaupt FP. How worthwhile is methylene blue as a treatment of malaria? Expert Rev Anti Infect Ther. 2019;17: 471-473.

72. Atamna H, Krugliak M, Shalmiev G, Deharo E, Pescarmona G, Ginsburg H. Mode of antimalarial effect of methylene blue and some of its analogues on *Plasmodium falciparum* in culture and their inhibition of *P. vinckei petteri* and *P. yoelii nigeriensis in vivo*. Biochem Pharmacol. 1996;51: 693-700.

73. Blank O, Davioud-Charvet E, Elhabiri M. Interactions of the antimalarial drug methylene blue with methemoglobin and heme targets in *Plasmodium falciparum*: a physico-biochemical study. Antioxid Redox Signal. 2012;17: 544-554.

74. Kumar S, Guha M, Choubey V, Maity P, Bandyopadhyay U. Antimalarial drugs inhibiting hemozoin (beta-hematin) formation: a mechanistic update. Life Sci. 2007;80: 813-828.

75. Färber PM, Arscott LD, Williams CH, Becker K, Schirmer RH. Recombinant *Plasmodium falciparum* glutathione reductase is inhibited by the antimalarial dye methylene blue. FEBS Lett. 1998;422: 311-314.

76. van Schalkwyk DA, Nash MN, Shafik SH, Summers RL, Lehane AM, Smith PJ, et al. Verapamil-sensitive transport of quinacrine and methylene blue via the *Plasmodium falciparum* chloroquine resistance transporter reduces the parasite's susceptibility to these tricyclic drugs. J Infect Dis. 2016;213: 800-810.

77. Gendrot M, Delandre O, Robert MG, Foguim FT, Benoit N, Amalvict R, et al. Absence of association between methylene blue reduced susceptibility and polymorphisms in 12 genes involved in antimalarial drug resistance in African *Plasmodium falciparum*. Pharmaceuticals. 2021;14: 351.

78. Mu J, Myers RA, Jiang H, Liu S, Ricklefs S, Waisberg M, et al. *Plasmodium falciparum* genome-wide scans for positive selection, recombination hot spots and resistance to antimalarial drugs. Nat Genet. 2010;42: 268-271.

79. Duraisingh MT, Roper C, Walliker D, Warhurst DC. Increased sensitivity to the antimalarials mefloquine and artemisinin is conferred by mutations in the *pfmdr1* gene of *Plasmodium falciparum*. Mol Microbiol. 2000;36: 955-961.
