# Supplementary material for: Mechanistic basis for multidrug resistance and collateral drug sensitivity conferred to the malaria parasite by polymorphisms in PfMDR1 and PfCRT
Source: PLoS Biol. 2022 May 4;20(5):e3001616. doi: 10.1371/journal.pbio.3001616 (PMC9067703; doi:10.1371/journal.pbio.3001616)
Supplement: S4 Text — (PDF) [file pbio.3001616.s018.pdf]

S4 Text: The multifactorial phenomenon of quinine resistance in the malaria parasite.

Polymorphisms in several transporters have been implicated in modulating the parasite's response to quinine — including changes in the genes encoding PfCRT, PfMDR1, the sodium-proton exchanger (PfNHE), and the multidrug resistance-associated protein 1 (PfMRP1). Whilst the interactions between quinine and several field isoforms of PfCRT have been characterized [1, 2], quinine's interaction with PfMDR1 is poorly understood. Here we measured the quinine transport activities of several field isoforms of PfMDR1 and PfCRT to provide greater insight into the possible roles of these two transporters in the parasite's acquisition of quinine resistance.

1. Quinine transport via PfCRT is associated with reduced parasite susceptibility to quinine.

We have previously used the *Xenopus* oocyte system to show that the mutant PfCRT isoforms carried by several strains of quinine-resistant parasites possess the ability to transport the drug, whereas the wild-type protein, and those from several quinine-sensitive parasites, do not exhibit this activity [1, 2]. Here we extend this analysis by showing that $PfCRT^{7G8}$ (an isoform expressed in quinine-tolerant parasites) also transports quinine, albeit with a much lower capacity than $PfCRT^{Dd2}$ (an isoform expressed in quinine-resistant parasites).

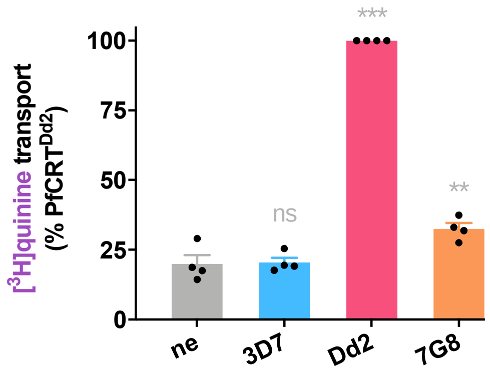

$PfCRT^{7G8}$ and $PfCRT^{Dd2}$ transport quinine whereas $PfCRT^{3D7}$ does not.

Our findings are consistent with previous measurements of quinine transport via PfCRT *in situ* using the H^+-efflux assay and the $C2^{GC03}$, $C6^{7G8}$, and $C4^{Dd2}$ lines [3, 4]. These studies indicated that both $PfCRT^{7G8}$ and $PfCRT^{Dd2}$ mediate the efflux of quinine out of the DV, whereas $PfCRT^{3D7}$ does not [3,

4]. Moreover, Sanchez et al. [5] compared the accumulation of $[^3H]$quinine in the same set of *pfcrt* isogenic lines and showed a reduction in quinine accumulation in $C6^{7G8}$ and $C4^{Dd2}$ parasites relative to the $C2^{GC03}$ line. Collectively, these findings confirm a role for PfCRT in the parasite's acquisition of quinine resistance that entails mutant isoforms of the transporter mediating the efflux of quinine from the DV, thereby reducing the access of the drug to its DV target (i.e. the inhibition of heme detoxification and/or another aspect of hemoglobin digestion [6]).

2. Absence of a clear correlation between rates of quinine transport via PfMDR1 in *Xenopus* oocytes and parasite quinine responses *in vitro*.

Our datasets indicate that the capacity of field PfMDR1 isoforms to transport quinine decreases in the order: $PfMDR1^{NFSDD}$ > $PfMDR1^{NYSND}$ > $PfMDR1^{FYSND}$ = $PfMDR1^{YFSND}$ = $PfMDR1^{YYSND}$ > $PfMDR1^{NFCDY}$ (Fig 4a and S1 Data). A similar trend is observed for the transport of quinidine, a stereoisomer of quinine (Fig 2d and S1 Data).

A previous study by Sanchez et al. [5] also measured quinine transport via PfMDR1 expressed in *Xenopus* oocytes, but they only detected quinine transport via $PfMDR1^{NYSND}$ and reported that $PfMDR1^{NFSDD}$, $PfMDR1^{YYSND}$, and $PfMDR1^{NFCDY}$ did not possess quinine transport activity. Given that the approach used by Sanchez et al. [5] resulted in a poor transport signal-to-background ratio for $PfMDR1^{NYSND}$ — they reported the rate of quinine transport to be ~55 fmol/oocyte/h, which is ~7.8-fold lower than the rate measured in our system (432 ± 13 fmol/oocyte/h) — it is very likely that the quinine transport activities of $PfMDR1^{YYSND}$ and $PfMDR1^{NFCDY}$ fell below the level of detection in their system. However, in a striking difference between our dataset and that of Sanchez et al. [5], we found that $PfMDR1^{NFSDD}$ exhibits a higher level of quinine transport activity than $PfMDR1^{NYSND}$. By contrast, Sanchez et al. [5] did not detect quinine transport via $PfMDR1^{NFSDD}$ at all. A possible explanation for this difference is that the expression conditions used by Sanchez et al. [5] (such as their use of the native *pfmdr1* sequence, rather than a sequence codon-harmonized for expression in *Xenopus* oocytes) resulted in the production of misfolded protein, rendering some PfMDR1 isoforms, such as $PfMDR1^{NFSDD}$, non-functional.

Importantly, the trends we observed in our oocyte system are consistent with previous *in vitro* measurements of *P. falciparum* responses to quinine. For example, Calçada et al. [7] showed that reversion of 86Y to N86 in Dd2 parasites (which express PfMDR1^{YYSND}) caused a ~1.6-fold decrease in the quinine IC_{50}. Consistent with this observation, we detected a ~1.6-fold decrease in the rate of quinine transport via PfMDR1^{YYSND} relative to PfMDR1^{NYSND}. Furthermore, Reed et al. [8] found that the replacement of PfMDR1^{NYSND} with PfMDR1^{NFCDY} in the chloroquine-sensitive D10 strain resulted in a ~1.9-fold increase in the quinine IC_{50}, which is in agreement with the ~2.3-fold decrease in the rate of quinine transport via PfMDR1^{NFCDY} relative to PfMDR1^{NYSND}. Together, these observations appear to suggest that PfMDR1 isoforms with low levels of quinine transport activity render the parasite less susceptible to quinine. However, an analysis of the relationship between the quinine transport capacities of PfMDR1 and the quinine resistance indices of the corresponding parasites (obtained from several published studies) did not identify a correlation between these two factors (Fig 5d and S3 Table). The most striking example of this observation is the similarity of the quinine resistance indices for HB3 and 7G8 parasites despite these strains expressing PfMDR1^{NFSDD} (which exhibits the highest capacity for quinine transport) or PfMDR1^{NFCDY} (which exhibits the lowest capacity for quinine transport), respectively. Moreover, exclusion of the 7G8 data point did not uncover a correlation between PfMDR1 quinine transport activity and the *in vitro* quinine resistance index. These findings suggest that although reductions in the ability of PfMDR1 to transport quinine may contribute to quinine resistance on specific genetic backgrounds (e.g., those carrying mutant PfCRT isoforms that have high quinine transport activities), polymorphisms in *pfmdr1* are not the main determinant of quinine resistance in the parasite.

3. The parasite's response to quinine is modulated by multiple factors.

Although the role of PfCRT in quinine resistance appears straightforward, some unusual *in vitro* observations highlight the complexity of the phenomenon of quinine resistance. Here we have shown that the 3D7 strain is more sensitive to quinine than HB3 parasites, which is in line with previous comparisons of the quinine susceptibilities of these two strains [9-11].

***In vitro* antiplasmodial activities of quinine and chloroquine against the 3D7, HB3, and Dd2 *P. falciparum* strains.**

Parasite strain	IC_{50} (nM)			
	Quinine		Chloroquine	
	Control	+ 1 µM VP	Control	+ 1 µM VP
3D7	112 ± 4	106 ± 3[ns]	13 ± 1	12 ± 1[ns]
HB3	309 ± 13***	304 ± 7[ns]	19 ± 1[ns]	13 ± 1[ns]
Dd2	524 ± 23***	298 ± 11***	136 ± 5***	56 ± 3***

In all cases, four independent experiments were performed (on different days), and within each experiment, measurements were averaged from three replicates. The error is the SEM.
The asterisks in blue denote comparisons between the 3D7 strain and another strain within either the quinine control or chloroquine control treatments.
The asterisks in black denote comparisons between the control treatment and the relevant verapamil (VP) treatment within the same strain.

Both 3D7 and HB3 parasites express the same isoform of PfCRT ($PfCRT^{3D7}$; an isoform that lacks the ability to transport quinine). Hence, the difference in quinine susceptibilities between these two strains cannot be attributed to PfCRT. The 3D7 and HB3 strains express $PfMDR1^{NYSND}$ and $PfMDR1^{NFSDD}$, respectively, and our datasets indicate that $PfMDR1^{NFSDD}$ has a higher capacity for quinine transport than $PfMDR1^{NYSND}$ (Figs 4a–4b and S1 Data). On this basis, it could be predicted that HB3 parasites would be more sensitive to quinine than 3D7 parasites because the increased transport capacity of $PfMDR1^{NFSDD}$ could increase the concentration of quinine within the DV and thus its activity against heme detoxification. However, the fact that 3D7 parasites are more sensitive to quinine than HB3 parasites suggests that the reduced sensitivity of the HB3 strain is conferred by one or more other genetic determinants (with PfMDR1 playing little or no role in the reduced susceptibility of this strain to quinine) and/or that quinine exerts an antimalarial effect on at least one other target.

In regard to the former possibility, quantitative trait analyses have implicated other genetic loci in modulating the parasite's susceptibility to quinine — including PfMRP1, PfNHE, and the homologous to E6-associated protein C-terminus (HECT) ubiquitin-protein ligase, PfUT. Although the roles of these proteins in altering the parasite's susceptibility to quinine are unresolved, polymorphisms in all three genes have been associated, but not definitively linked, with reduced sensitivity to the drug [10, 12-14]. Genetic disruption of PfMRP1 *in vitro* indicated that the protein is involved in the removal of quinine from the parasite [15]. *In vitro* studies have also suggested that PfUT and PfNHE both contribute to quinine resistance in a specific genetic context; polymorphisms in these genes can alter the potency of the drug, but only in the presence of a chloroquine resistance-conferring isoform of PfCRT ($PfCRT^{Dd2}$) [12, 13, 16, 17]. More recently, the overexpression of PfUT in 3D7 parasites has

been shown to alter the length of the parasite's cell cycle [18], which could trigger the differential expression of genes that provide advantages to the parasite in the presence of quinine.

It is also possible that the detoxification of heme (or another aspect of hemoglobin digestion) is not the primary target of quinine. A previous study demonstrated that the K1 and W2mef strains, which both carry a chloroquine-resistance-conferring isoform of PfCRT as well as multiple copies of *pfmdr1*, were less susceptible to quinine than parasites with fewer *pfmdr1* copies [19]. Indeed, several studies have associated multicopy *pfmdr1* with reduced quinine susceptibility [19-21]. This indicates that having higher levels of PfMDR1 expression, which should lead to higher rates of quinine transport into the DV, is advantageous under quinine pressure for parasites of certain genetic backgrounds.

Given these observations, it is tempting to propose the same mechanistic model for quinine resistance as we have for mefloquine and lumefantrine resistance, whereby an increase in the rate of quinine transport into the DV via PfMDR1 (resulting from the overexpression of PfMDR1 and/or the presence of PfMDR1^{NFSDD}) sequesters the drug away from a cytosolic target that is quinine's true primary site of action. Several cytosolic targets have been proposed for quinine. For example, quinine was originally thought to interfere with vesicular docking by binding to phospholipids, resulting in eventual parasite death [22]. More recently, the parasite's purine nucleoside phosphorylase (PfPNP) was implicated as the primary target of quinine [23]. Although the location of this enzyme within the *P. falciparum*-infected erythrocyte is currently unknown, its homologue in *Toxoplasma gondii* displays cytosolic localization [24], suggesting that PfPNP may likewise be located in the malaria parasite's cytosol.

Another factor to consider is whether increases in the *pfmdr1* copy number alter the trafficking of the protein in the parasite. Immunofluorescence assays have revealed that PfMDR1 is predominantly located in the DV membrane, but the protein is also found in the parasite's plasma membrane [25]. Hence, the parasite could potentially modulate its response to quinine (and other antimalarial drugs) by regulating the level of PfMDR1 protein at each membrane (in a manner similar to that described for the regulation of human P-gp function [26-28]). For example, preferentially increasing the expression of PfMDR1 at the plasma membrane could significantly increase the rate of drug export from the

parasite cytosol. Indeed, both transcriptomic and western blot analyses have indicated that parasites expressing multiple copies of *pfmdr1* express greater levels of *pfmdr1* transcript levels and PfMDR1 protein [29-31]. However, there has been no evidence to date that the additional PfMDR1 protein is preferentially trafficked to the parasite plasma membrane. Instead, parasites expressing multiple copies of *pfmdr1* exhibit a stronger fluorescence signal in the DV membrane relative to parasites expressing one copy of *pfmdr1* [25].

In any case, the association between mutant PfCRT isoforms that have high capacities for exporting quinine from the DV and reductions in the parasite's susceptibility to quinine is at odds with quinine exerting its primary antimalarial effect in the cytosol. It appears, therefore, that the role of PfMDR1 in quinine resistance is more complex and nuanced than simply either causing a decrease or an increase in the accumulation of the drug within the DV (i.e. the roles we have proposed for PfMDR1 in chloroquine resistance and mefloquine/lumefantrine resistance, respectively). In this regard, it is worth noting that whilst the wild-type protein exhibits similar affinities for the binding of quinine, mefloquine, and lumefantrine, the transport of quinine occurs at a substantially lower rate than the transport of mefloquine or lumefantrine (Fig 3 and Fig 4). It is possible that the relatively low rate of quinine translocation impedes the transport of the natural substrates of PfMDR1 and, given that the normal function of the transporter is essential for parasite survival, this inhibition could exert a killing effect that would be overcome by overexpression of PfMDR1 in the DV membrane. With this possibility in mind, it may be that the complex and variable relationship between quinine susceptibility and polymorphisms in *pfcrt* and *pfmdr1* centres on two key factors: (1) the net flux of quinine across the DV membrane and (2) the rate at which PfMDR1's natural substrates are transported into the DV. Whilst overexpression of PfMDR1 would overcome the (proposed) antimalarial effect of quinine's inhibition of the natural function of the transporter, the concomitant increase in the PfMDR1-mediated transport of quinine into the DV could increase the drug's ability to inhibit heme detoxification. However, this scenario would be circumvented if the rate of quinine efflux via PfCRT was sufficient to cause a net decrease in the concentration of quinine within the DV. That is, if the rate of quinine efflux via PfCRT is greater than the total rate of quinine influx (via both PfMDR1 and simple diffusion), there will be a decrease in the accumulation of quinine at its primary site of antimalarial action. Support for

this mechanistic model could be forthcoming if the natural substrates of PfMDR1 are identified and if their transport is shown to be significantly inhibited by quinine.

References

1. Bellanca S, Summers RL, Meyrath M, Dave A, Nash MN, Dittmer M, et al. Multiple drugs compete for transport via the *Plasmodium falciparum* chloroquine resistance transporter at distinct but interdependent sites. J Biol Chem. 2014;289: 36336-36351.

2. Richards SN, Nash MN, Baker ES, Webster MW, Lehane AM, Shafik SH, et al. Molecular mechanisms for drug hypersensitivity induced by the malaria parasite's chloroquine resistance transporter. PLoS Pathog. 2016;12: e1005725.

3. Hrycyna CA, Summers RL, Lehane AM, Pires MM, Namanja H, Bohn K, et al. Quinine dimers are potent inhibitors of the *Plasmodium falciparum* chloroquine resistance transporter and are active against quinoline-resistant *P. falciparum*. ACS Chem Biol. 2014;9: 722-730.

4. Lehane AM, Kirk K. Efflux of a range of antimalarial drugs and 'chloroquine resistance reversers' from the digestive vacuole in malaria parasites with mutant PfCRT. Mol Microbiol. 2010;77: 1039-1051.

5. Sanchez CP, Rotmann A, Stein WD, Lanzer M. Polymorphisms within PfMDR1 alter the substrate specificity for anti-malarial drugs in *Plasmodium falciparum*. Mol Microbiol. 2008;70: 786-798.

6. Bohorquez E, Chua M, Meshnick S. Quinine localizes to a non-acidic compartment within the food vacuole of the malaria parasite *Plasmodium falciparum*. Malar J. 2012;11: 350.

7. Calçada C, Silva M, Baptista V, Thathy V, Silva-Pedrosa R, Granja D, et al. Expansion of a specific *Plasmodium falciparum* PfMDR1 haplotype in Southeast Asia with increased substrate transport. mBio. 2020;11: e02093-02020.

8. Reed MB, Saliba KJ, Caruana SR, Kirk K, Cowman AF. Pgh1 modulates sensitivity and resistance to multiple antimalarials in *Plasmodium falciparum*. Nature. 2000;403: 906-909.

9. Duraisingh MT, Roper C, Walliker D, Warhurst DC. Increased sensitivity to the antimalarials mefloquine and artemisinin is conferred by mutations in the *pfmdr1* gene of *Plasmodium falciparum*. Mol Microbiol. 2000;36: 955-961.

10. Mu J, Ferdig MT, Feng X, Joy DA, Duan J, Furuya T, et al. Multiple transporters associated with malaria parasite responses to chloroquine and quinine. Mol Microbiol. 2003;49: 977-989.

11. Johnson DJ, Fidock DA, Mungthin M, Lakshmanan V, Sidhu AB, Bray PG, et al. Evidence for a central role for PfCRT in conferring *Plasmodium falciparum* resistance to diverse antimalarial agents. Mol Cell. 2004;15: 867-877.

12. Sanchez CP, Liu CH, Mayer S, Nurhasanah A, Cyrklaff M, Mu J, et al. A HECT ubiquitin-protein ligase as a novel candidate gene for altered quinine and quinidine responses in *Plasmodium falciparum*. PLoS Genet. 2014;10: e1004382.

13. Ferdig MT, Cooper RA, Mu J, Deng B, Joy DA, Su X-z, et al. Dissecting the loci of low-level quinine resistance in malaria parasites. Mol Microbiol. 2004;52: 985-997.

14. Wu K, Yao Y, Chen F, Xu M, Lu G, Jiang T, et al. Analysis of *Plasmodium falciparum* Na^+/H^+ exchanger (*pfnhe1*) polymorphisms among imported African malaria parasites isolated in Wuhan, Central China. BMC Infect Dis. 2019;19: 354.

15. Raj DK, Mu J, Jiang H, Kabat J, Singh S, Sullivan M, et al. Disruption of a *Plasmodium falciparum* multidrug resistance-associated protein (PfMRP) alters its fitness and transport of antimalarial drugs and glutathione. J Biol Chem. 2009;284: 7687-7696.

16. Briolant S, Pelleau S, Bogreau H, Hovette P, Zettor A, Castello J, et al. *In vitro* susceptibility to quinine and microsatellite variations of the *Plasmodium falciparum* Na^+/H^+ exchanger (*Pfnhe-1*) gene: the absence of association in clinical isolates from the Republic of Congo. Malar J. 2011;10: 37.

17. Nkrumah LJ, Riegelhaupt PM, Moura P, Johnson DJ, Patel J, Hayton K, et al. Probing the multifactorial basis of *Plasmodium falciparum* quinine resistance: evidence for a strain-specific contribution of the sodium-proton exchanger PfNHE. Mol Biochem Parasitol. 2009;165: 122-131.

18. Jankowska-Döllken M, Sanchez CP, Cyrklaff M, Lanzer M. Overexpression of the HECT ubiquitin ligase PfUT prolongs the intraerythrocytic cycle and reduces invasion efficiency of *Plasmodium falciparum*. Sci Rep. 2019;9: 18333.

19. Cowman AF, Galatis D, Thompson JK. Selection for mefloquine resistance in *Plasmodium falciparum* is linked to amplification of the *pfmdr1* gene and cross-resistance to halofantrine and quinine. Proc Natl Acad Sci USA. 1994;91: 1143-1147.

20. Peel SA, Bright P, Yount B, Handy J, Baric RS. A strong association between mefloquine and halofantrine resistance and amplification, overexpression, and mutation in the P-glycoprotein gene homolog (*pfmdr*) of *Plasmodium falciparum in vitro*. Am J Trop Med Hyg. 1994;51: 648-658.

21. Sidhu AB, Valderramos SG, Fidock DA. *pfmdr1* mutations contribute to quinine resistance and enhance mefloquine and artemisinin sensitivity in *Plasmodium falciparum*. Mol Microbiol. 2005;57: 913-926.

22. Jacobs GH, Aikawa M, Milhous WK, Rabbege JR. An ultrastructural study of the effects of mefloquine on malaria parasites. Am J Trop Med Hyg. 1987;36: 9-14.

23. Dziekan JM, Yu H, Chen D, Dai L, Wirjanata G, Larsson A, et al. Identifying purine nucleoside phosphorylase as the target of quinine using cellular thermal shift assay. Sci Transl Med. 2019;11: eaau3174.

24. Gherardi A, Peyrol S, Sarciron M. *Toxoplasma gondii*: localization of purine nucleoside phosphorylase activity *in vitro* and *in vivo* by electron microscopy. Med Mol Morphol. 2005;38: 251-255.

25. Cowman AF, Karcz S, Galatis D, Culvenor JG. A P-glycoprotein homologue of *Plasmodium falciparum* is localized on the digestive vacuole. J Cell Biol. 1991;113: 1033-1042.

26. Noack A, Noack S, Hoffmann A, Maalouf K, Buettner M, Couraud PO, et al. Drug-induced trafficking of P-glycoprotein in human brain capillary endothelial cells as demonstrated by exposure to mitomycin C. PLoS One. 2014;9: e88154.

27. Tome ME, Herndon JM, Schaefer CP, Jacobs LM, Zhang Y, Jarvis CK, et al. P-glycoprotein traffics from the nucleus to the plasma membrane in rat brain endothelium during inflammatory pain. J Cereb Blood Flow Metab. 2016;36: 1913-1928.

28. Schaefer CP, Arkwright NB, Jacobs LM, Jarvis CK, Hunn KC, Largent-Milnes TM, et al. Chronic morphine exposure potentiates p-glycoprotein trafficking from nuclear reservoirs in cortical rat brain microvessels. PLoS One. 2018;13: e0192340.

29. Reiling SJ, Rohrbach P. Monitoring PfMDR1 transport in *Plasmodium falciparum*. Malar J. 2015;14: 270.

30. Adjalley SH, Scanfeld D, Kozlowski E, Llinás M, Fidock DA. Genome-wide transcriptome profiling reveals functional networks involving the *Plasmodium falciparum* drug resistance transporters PfCRT and PfMDR1. BMC Genomics. 2015;16: 1090.

31. Jiang H, Patel JJ, Yi M, Mu J, Ding J, Stephens R, et al. Genome-wide compensatory changes accompany drug-selected mutations in the *Plasmodium falciparum crt* gene. PLoS One. 2008;3: e2484.

32. Sanchez CP, Mayer S, Nurhasanah A, Stein WD, Lanzer M. Genetic linkage analyses redefine the roles of PfCRT and PfMDR1 in drug accumulation and susceptibility in *Plasmodium falciparum*. Mol Microbiol. 2011;82: 865-878.

33. Veiga MI, Dhingra SK, Henrich PP, Straimer J, Gnadig N, Uhlemann AC, et al. Globally prevalent PfMDR1 mutations modulate *Plasmodium falciparum* susceptibility to artemisinin-based combination therapies. Nat Commun. 2016;7: 11553.
