# Supplementary material for: Mechanistic basis for multidrug resistance and collateral drug sensitivity conferred to the malaria parasite by polymorphisms in PfMDR1 and PfCRT
Source: PLoS Biol. 2022 May 4;20(5):e3001616. doi: 10.1371/journal.pbio.3001616 (PMC9067703; doi:10.1371/journal.pbio.3001616)
Supplement: S1 File — PfMDR1, Plasmodium falciparum multidrug resistance protein 1. (PDF) [file pbio.3001616.s019.pdf]

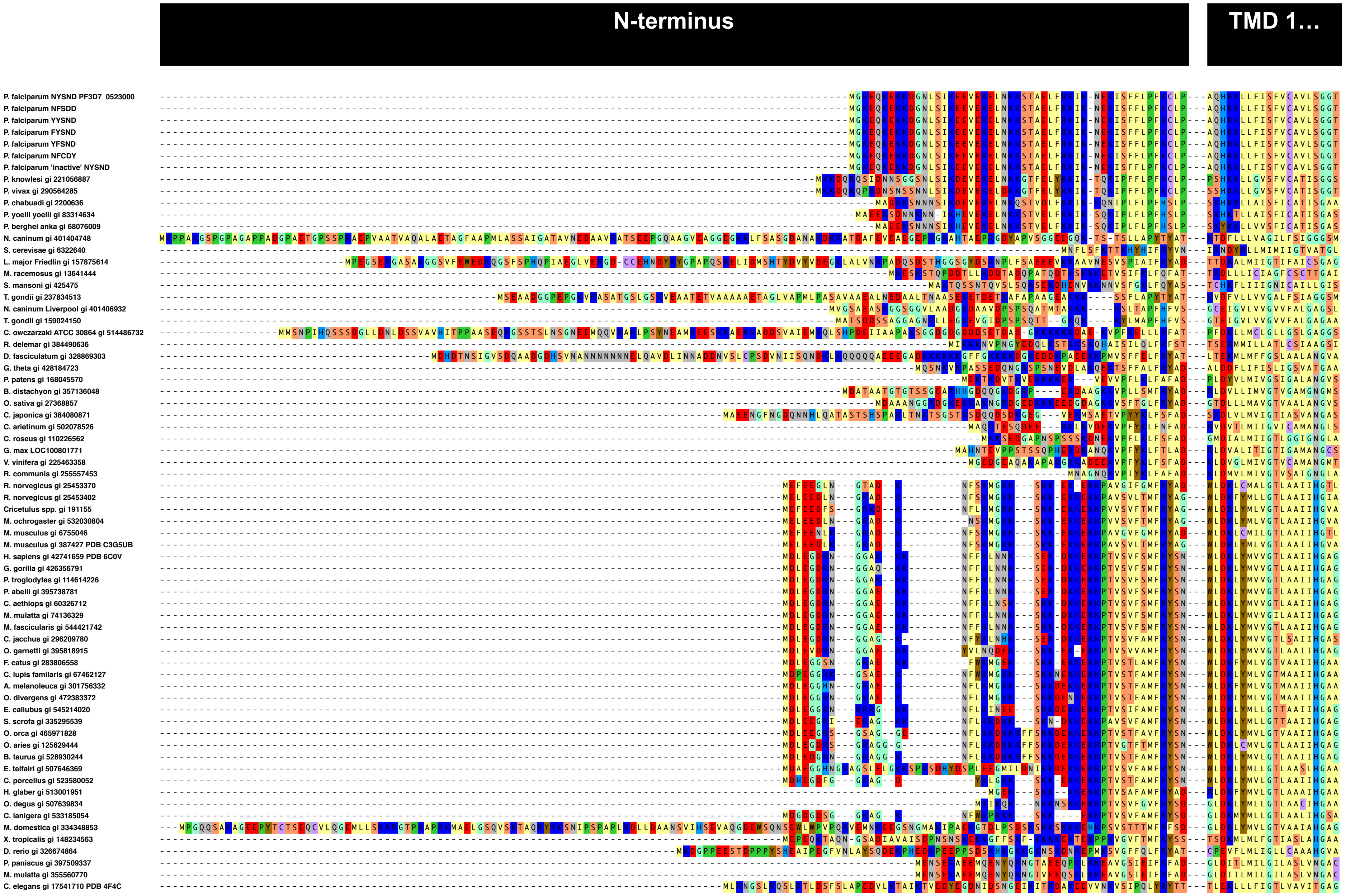
N-terminus
TMD 1…
P. falciparum NYSND PF3D7_0523000
P. falciparum NFSDD
P. falciparum YYSND
P. falciparum FYSND
P. falciparum YFSND
P. falciparum NFCDY
P. falciparum 'inactive' NYSND
P. knowlesi gi 221056887
P. vivax gi 290564285
P. chabaudi gi 2200636
P. yoelii yoelii gi 83314634
P. berghei anka gi 68076009
N. caninum gi 401404748
S. cerevisae gi 6322640
L. major Friedlin gi 157875614
M. racemosus gi 13641444
S. mansoni gi 425475
T. gondii gi 237834513
N. caninum Liverpool gi 401406932
T. gondii gi 159024150
C. owczarzaki ATCC 30864 gi 514486732
R. delemar gi 384490636
D. fasciculatum gi 328869303
G. theta gi 428184723
P. patens gi 168045570
B. distachyon gi 357136048
O. sativa gi 27368857
C. japonica gi 384080871
C. arietinum gi 502078526
C. roseus gi 110226562
G. max LOC100801771
V. vinifera gi 225463358
R. communis gi 255557453
R. norvegicus gi 25453370
R. norvegicus gi 25453402
Cricetulus spp. gi 191155
M. ochrogaster gi 532030804
M. musculus gi 6755046
M. musculus gi 387427 PDB C3G5UB
H. sapiens gi 42741659 PDB 6C0V
G. gorilla gi 426356791
P. troglodytes gi 114614226
P. abelii gi 395738781
C. aethiops gi 60326712
M. mulatta gi 74136329
M. fascicularis gi 544421742
C. jacchus gi 296209780
O. garnetti gi 395818915
F. catus gi 283806558
C. lupis familaris gi 67462127
A. melanoleuca gi 301756332
O. divergens gi 472383372
E. callubus gi 545214020
S. scrofa gi 335295539
O. orca gi 465971828
O. aries gi 125629444
B. taurus gi 528930244
E. telfairi gi 507646369
C. porcellus gi 523580052
H. glaber gi 513001951
O. degus gi 507639834
C. lanigera gi 533185054
M. domestica gi 334348853
X. tropicalis gi 148234563
D. rerio gi 326674864
P. paniscus gi 397509337
M. mulatta gi 355560770
C. elegans gi 17541710 PDB 4F4C

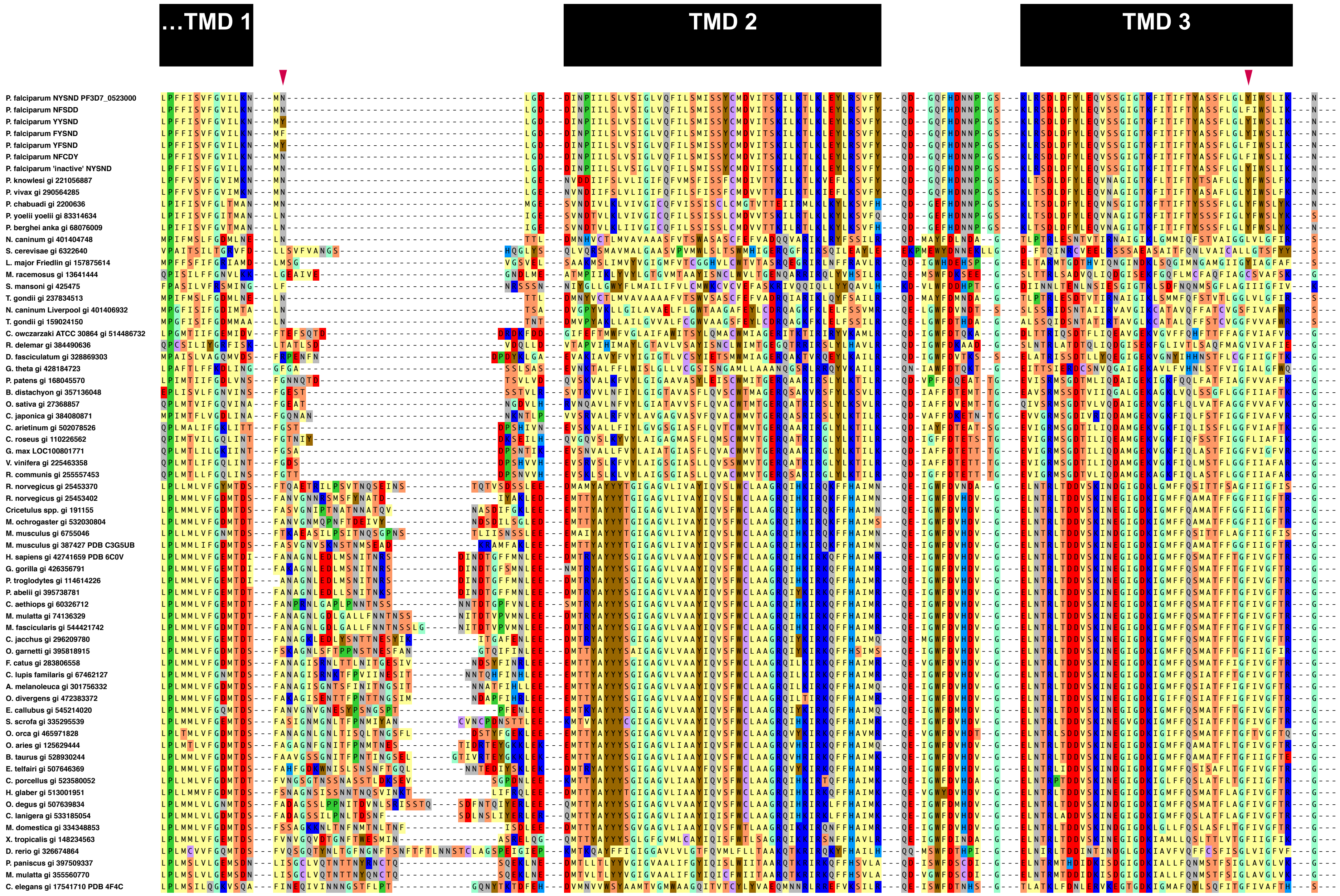

…TMD 1
TMD 2
TMD 3
P. falciparum NYSND PF3D7_0523000
P. falciparum NFSDD
P. falciparum YYSND
P. falciparum FYSND
P. falciparum YFSND
P. falciparum NFCDY
P. falciparum 'inactive' NYSND
P. knowlesi gi 221056887
P. vivax gi 290564285
P. chabaudi gi 2200636
P. yoelii yoelii gi 83314634
P. berghei anka gi 68076009
N. caninum gi 401404748
S. cerevisae gi 6322640
L. major Friedlin gi 157875614
M. racemosus gi 13641444
S. mansoni gi 425475
T. gondii gi 237834513
N. caninum Liverpool gi 401406932
T. gondii gi 159024150
C. owczarzaki ATCC 30864 gi 514486732
R. delemar gi 384490636
D. fasciculatum gi 328869303
G. theta gi 428184723
P. patens gi 168045570
B. distachyon gi 357136048
O. sativa gi 27368857
C. japonica gi 384080871
C. arietinum gi 502078526
C. roseus gi 110226562
G. max LOC100801771
V. vinifera gi 225463358
R. communis gi 255557453
R. norvegicus gi 25453370
R. norvegicus gi 25453402
Cricetulus spp. gi 191155
M. ochrogaster gi 532030804
M. musculus gi 6755046
M. musculus gi 387427 PDB C3G5UB
H. sapiens gi 42741659 PDB 6C0V
G. gorilla gi 426356791
P. troglodytes gi 114614226
P. abelii gi 395738781
C. aethiops gi 60326712
M. mulatta gi 74136329
M. fascicularis gi 544421742
C. jacchus gi 296209780
O. garnetti gi 395818915
F. catus gi 283806558
C. lupis familaris gi 67462127
A. melanoleuca gi 301756332
O. divergens gi 472383372
E. callubus gi 545214020
S. scrofa gi 335295539
O. orca gi 465971828
O. aries gi 125629444
B. taurus gi 528930244
E. telfairi gi 507646369
C. porcellus gi 523580052
H. glaber gi 513001951
O. degus gi 507639834
C. lanigera gi 533185054
M. domestica gi 334348853
X. tropicalis gi 148234563
D. rerio gi 326674864
P. paniscus gi 397509337
M. mulatta gi 355560770
C. elegans gi 17541710 PDB 4F4C

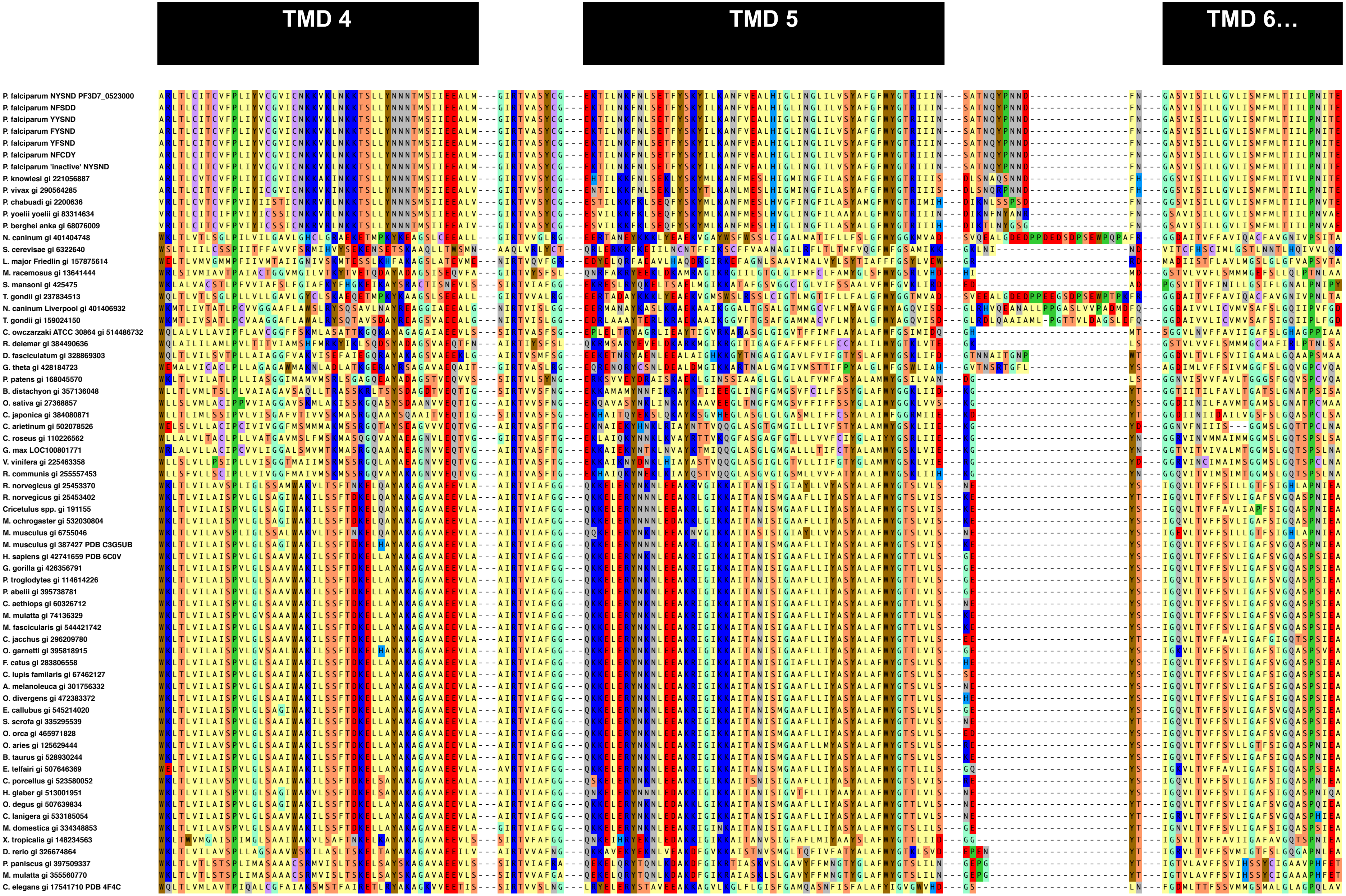
TMD 4
TMD 5
TMD 6...
P. falciparum NYSND PF3D7_0523000
P. falciparum NFSDD
P. falciparum YYSND
P. falciparum FYSND
P. falciparum YFSND
P. falciparum NFCDY
P. falciparum 'inactive' NYSND
P. knowlesi gi 221056887
P. vivax gi 290564285
P. chabuadi gi 2200636
P. yoelii yoelii gi 83314634
P. berghei anka gi 68076009
N. caninum gi 401404748
S. cerevisae gi 6322640
L. major Friedlin gi 157875614
M. racemosus gi 13641444
S. mansoni gi 425475
T. gondii gi 237834513
N. caninum Liverpool gi 401406932
T. gondii gi 159024150
C. owczarzaki ATCC 30864 gi 514486732
R. delemar gi 384490636
D. fasciculatum gi 328869303
G. theta gi 428184723
P. patens gi 168045570
B. distachyon gi 357136048
O. sativa gi 27368857
C. japonica gi 384080871
C. arietinum gi 502078526
C. roseus gi 110226562
G. max LOC100801771
V. vinifera gi 225463358
R. communis gi 255557453
R. norvegicus gi 25453370
R. norvegicus gi 25453402
Cricetulus spp. gi 191155
M. ochrogaster gi 532030804
M. musculus gi 6755046
M. musculus gi 387427 PDB C3G5UB
H. sapiens gi 42741659 PDB 6C0V
G. gorilla gi 426356791
P. troglodytes gi 114614226
P. abelii gi 395738781
C. aethiops gi 60326712
M. mulatta gi 74136329
M. fascicularis gi 544421742
C. jacchus gi 296209780
O. garnetti gi 395818915
F. catus gi 283806558
C. lupis familaris gi 67462127
A. melanoleuca gi 301756332
O. divergens gi 472383372
E. callubus gi 545214020
S. scrofa gi 335295539
O. orca gi 465971828
O. aries gi 125629444
B. taurus gi 528930244
E. telfairi gi 507646369
C. porcellus gi 523580052
H. glaber gi 513001951
O. degus gi 507639834
C. lanigera gi 533185054
M. domestica gi 334348853
X. tropicalis gi 148234563
D. rerio gi 326674864
P. paniscus gi 397509337
M. mulatta gi 355560770
C. elegans gi 17541710 PDB 4F4C

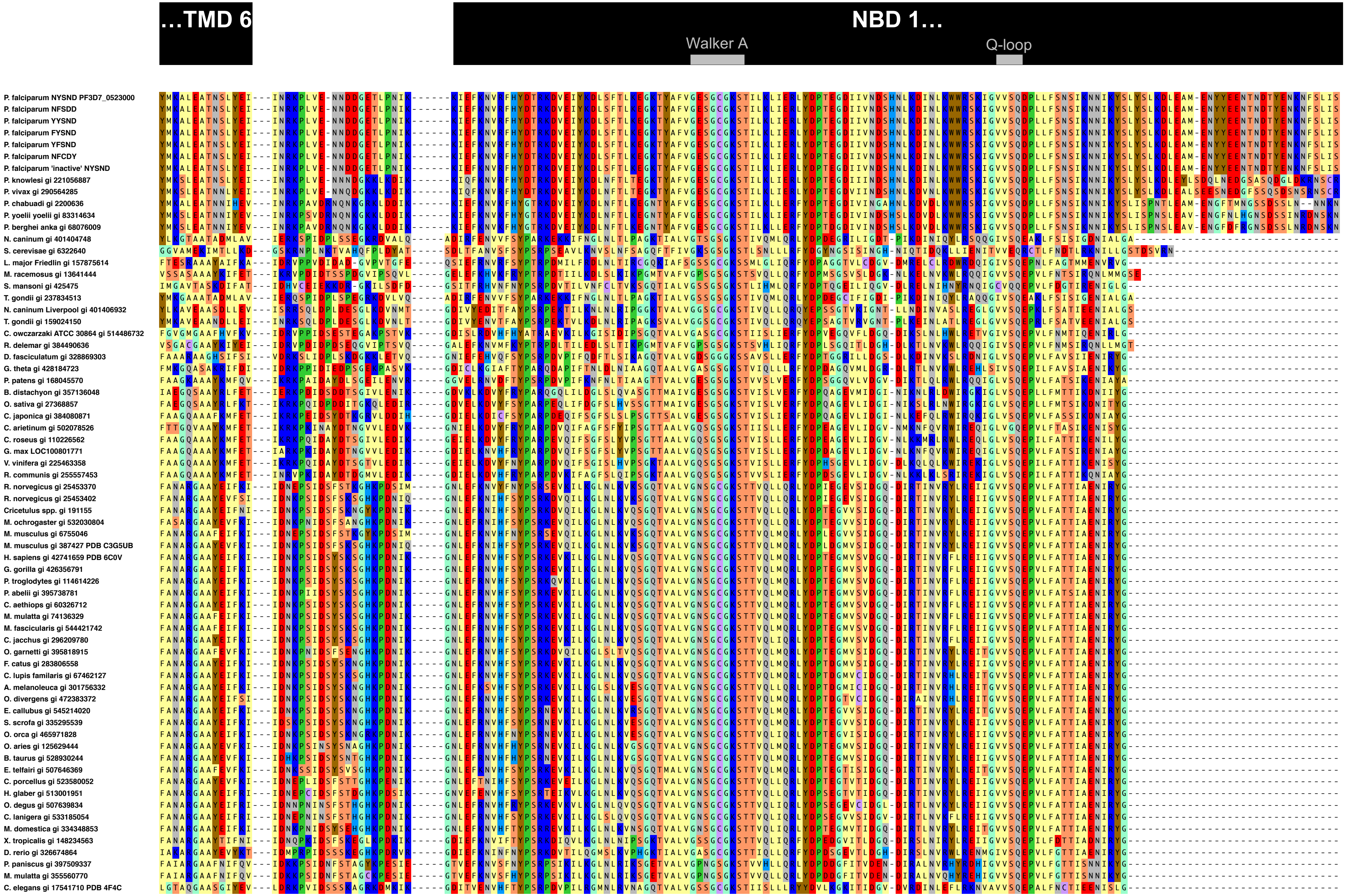
…TMD 6
NBD 1…
Walker A
Q-loop
P. falciparum NYSND PF3D7_0523000
P. falciparum NFSDD
P. falciparum YYSND
P. falciparum FYSND
P. falciparum YFSND
P. falciparum NFCDY
P. falciparum 'inactive' NYSND
P. knowlesi gi 221056887
P. vivax gi 290564285
P. chabaudi gi 2200636
P. yoelii yoelii gi 83314634
P. berghei anka gi 68076009
N. caninum gi 401404748
S. cerevisae gi 6322640
L. major Friedlin gi 157875614
M. racemosus gi 13641444
S. mansoni gi 425475
T. gondii gi 237834513
N. caninum Liverpool gi 401406932
T. gondii gi 159024150
C. owczarzaki ATCC 30864 gi 514486732
R. delemar gi 384490636
D. fasciculatum gi 328869303
G. theta gi 428184723
P. patens gi 168045570
B. distachyon gi 357136048
O. sativa gi 27368857
C. japonica gi 384080871
C. arietinum gi 502078526
C. roseus gi 110226562
G. max LOC100801771
V. vinifera gi 225463358
R. communis gi 255557453
R. norvegicus gi 25453370
R. norvegicus gi 25453402
Cricetulus spp. gi 191155
M. ochrogaster gi 532030804
M. musculus gi 6755046
M. musculus gi 387427 PDB C3G5UB
H. sapiens gi 42741659 PDB 6C0V
G. gorilla gi 426356791
P. troglodytes gi 114614226
P. abelii gi 395738781
C. aethiops gi 60326712
M. mulatta gi 74136329
M. fascicularis gi 544421742
C. jacchus gi 296209780
O. garnetti gi 395818915
F. catus gi 283806558
C. lupis familaris gi 67462127
A. melanoleuca gi 301756332
O. divergens gi 472383372
E. callubus gi 545214020
S. scrofa gi 335295539
O. orca gi 465971828
O. aries gi 125629444
B. taurus gi 528930244
E. telfairi gi 507646369
C. porcellus gi 523580052
H. glaber gi 513001951
O. degus gi 507639834
C. lanigera gi 533185054
M. domestica gi 334348853
X. tropicalis gi 148234563
D. rerio gi 326674864
P. paniscus gi 397509337
M. mulatta gi 355560770
C. elegans gi 17541710 PDB 4F4C

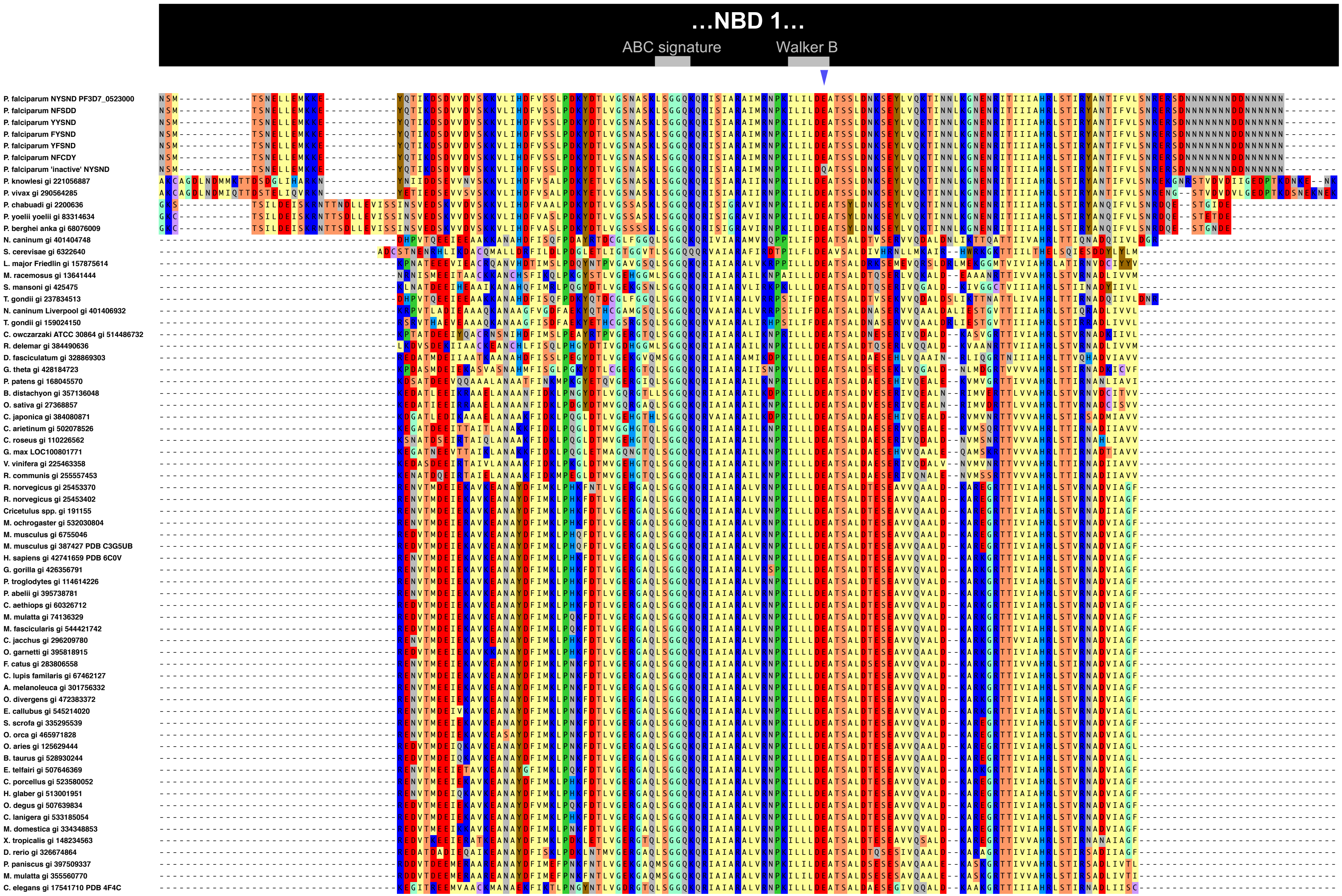
…NBD 1…
ABC signature
Walker B
P. falciparum NYSND PF3D7_0523000
P. falciparum NFSDD
P. falciparum YYSND
P. falciparum FYSND
P. falciparum YFSND
P. falciparum NFCDY
P. falciparum 'inactive' NYSND
P. knowlesi gi 221056887
P. vivax gi 290564285
P. chabaudi gi 2200636
P. yoelii yoelii gi 83314634
P. berghei anka gi 68076009
N. caninum gi 401404748
S. cerevisae gi 6322640
L. major Friedlin gi 157875614
M. racemosus gi 13641444
S. mansoni gi 425475
T. gondii gi 237834513
N. caninum Liverpool gi 401406932
T. gondii gi 159024150
C. owczarzaki ATCC 30864 gi 514486732
R. delemar gi 384490636
D. fasciculatum gi 328869303
G. theta gi 428184723
P. patens gi 168045570
B. distachyon gi 357136048
O. sativa gi 27368857
C. japonica gi 384080871
C. arietinum gi 502078526
C. roseus gi 110226562
G. max LOC100801771
V. vinifera gi 225463358
R. communis gi 255557453
R. norvegicus gi 25453370
R. norvegicus gi 25453402
Cricetulus spp. gi 191155
M. ochrogaster gi 532030804
M. musculus gi 6755046
M. musculus gi 387427 PDB C3G5UB
H. sapiens gi 42741659 PDB 6C0V
G. gorilla gi 426356791
P. troglodytes gi 114614226
P. abelii gi 395738781
C. aethiops gi 60326712
M. mulatta gi 74136329
M. fascicularis gi 544421742
C. jacchus gi 296209780
O. garnetti gi 395818915
F. catus gi 283806558
C. lupis familaris gi 67462127
A. melanoleuca gi 301756332
O. divergens gi 472383372
E. callubus gi 545214020
S. scrofa gi 335295539
O. orca gi 465971828
O. aries gi 125629444
B. taurus gi 528930244
E. telfairi gi 507646369
C. porcellus gi 523580052
H. glaber gi 513001951
O. degus gi 507639834
C. lanigera gi 533185054
M. domestica gi 334348853
X. tropicalis gi 148234563
D. rerio gi 326674864
P. paniscus gi 397509337
M. mulatta gi 355560770
C. elegans gi 17541710 PDB 4F4C

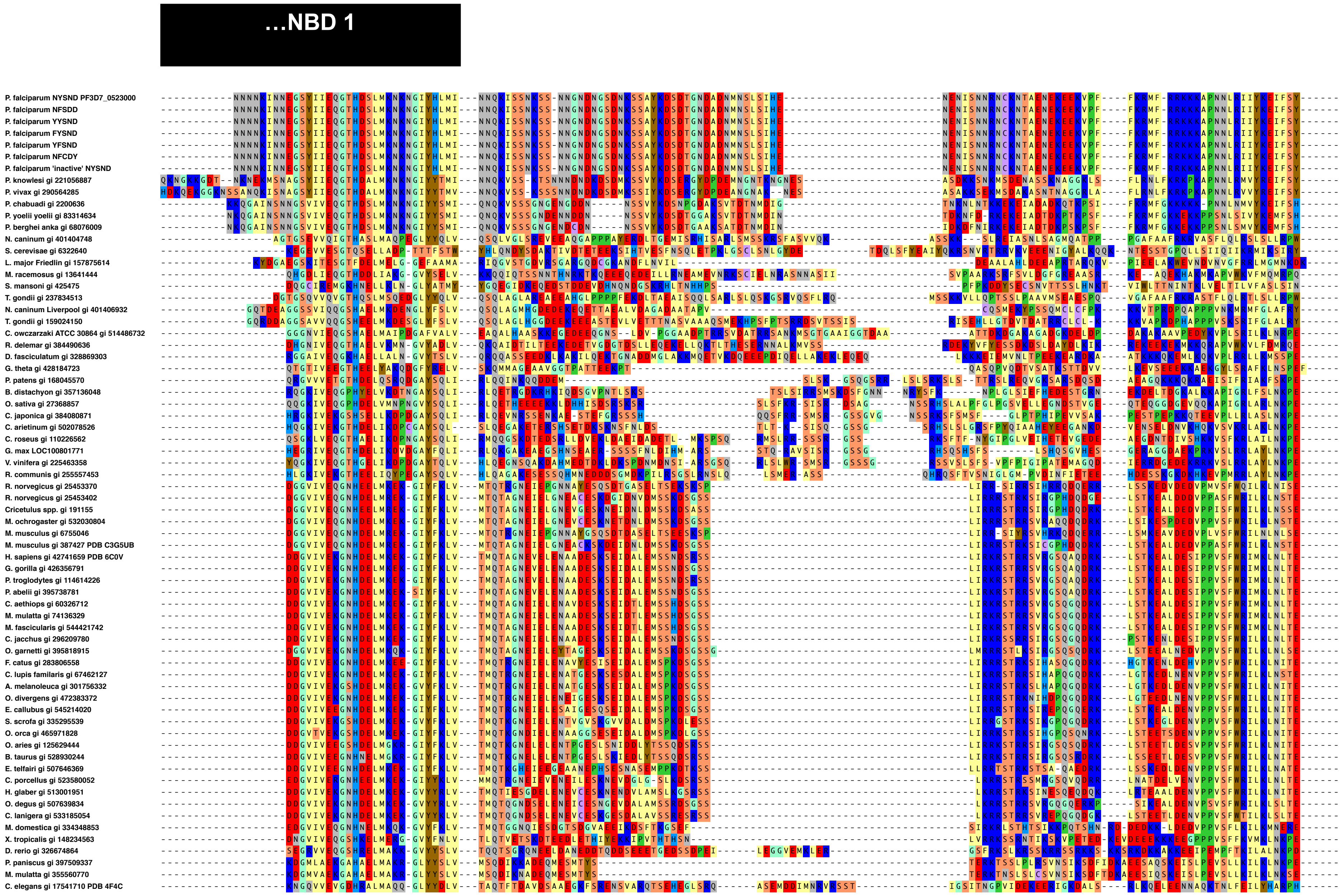
…NBD 1
P. falciparum NYSND PF3D7_0523000
P. falciparum NFSDD
P. falciparum YYSND
P. falciparum FYSND
P. falciparum YFSND
P. falciparum NFCDY
P. falciparum 'inactive' NYSND
P. knowlesi gi 221056887
P. vivax gi 290564285
P. chabaudi gi 2200636
P. yoelii yoelii gi 83314634
P. berghei anka gi 68076009
N. caninum gi 401404748
S. cerevisae gi 6322640
L. major Friedlin gi 157875614
M. racemosus gi 13641444
S. mansoni gi 425475
T. gondii gi 237834513
N. caninum Liverpool gi 401406932
T. gondii gi 159024150
C. owczarzaki ATCC 30864 gi 514486732
R. delemar gi 384490636
D. fasciculatum gi 328869303
G. theta gi 428184723
P. patens gi 168045570
B. distachyon gi 357136048
O. sativa gi 27368857
C. japonica gi 384080871
C. arietinum gi 502078526
C. roseus gi 110226562
G. max LOC100801771
V. vinifera gi 225463358
R. communis gi 255557453
R. norvegicus gi 25453370
R. norvegicus gi 25453402
Cricetulus spp. gi 191155
M. ochrogaster gi 532030804
M. musculus gi 6755046
M. musculus gi 387427 PDB C3G5UB
H. sapiens gi 42741659 PDB 6C0V
G. gorilla gi 426356791
P. troglodytes gi 114614226
P. abelii gi 395738781
C. aethiops gi 60326712
M. mulatta gi 74136329
M. fascicularis gi 544421742
C. jacchus gi 296209780
O. garnetti gi 395818915
F. catus gi 283806558
C. lupis familaris gi 67462127
A. melanoleuca gi 301756332
O. divergens gi 472383372
E. callubus gi 545214020
S. scrofa gi 335295539
O. orca gi 465971828
O. aries gi 125629444
B. taurus gi 528930244
E. telfairi gi 507646369
C. porcellus gi 523580052
H. glaber gi 513001951
O. degus gi 507639834
C. lanigera gi 533185054
M. domestica gi 334348853
X. tropicalis gi 148234563
D. rerio gi 326674864
P. paniscus gi 397509337
M. mulatta gi 355560770
C. elegans gi 17541710 PDB 4F4C

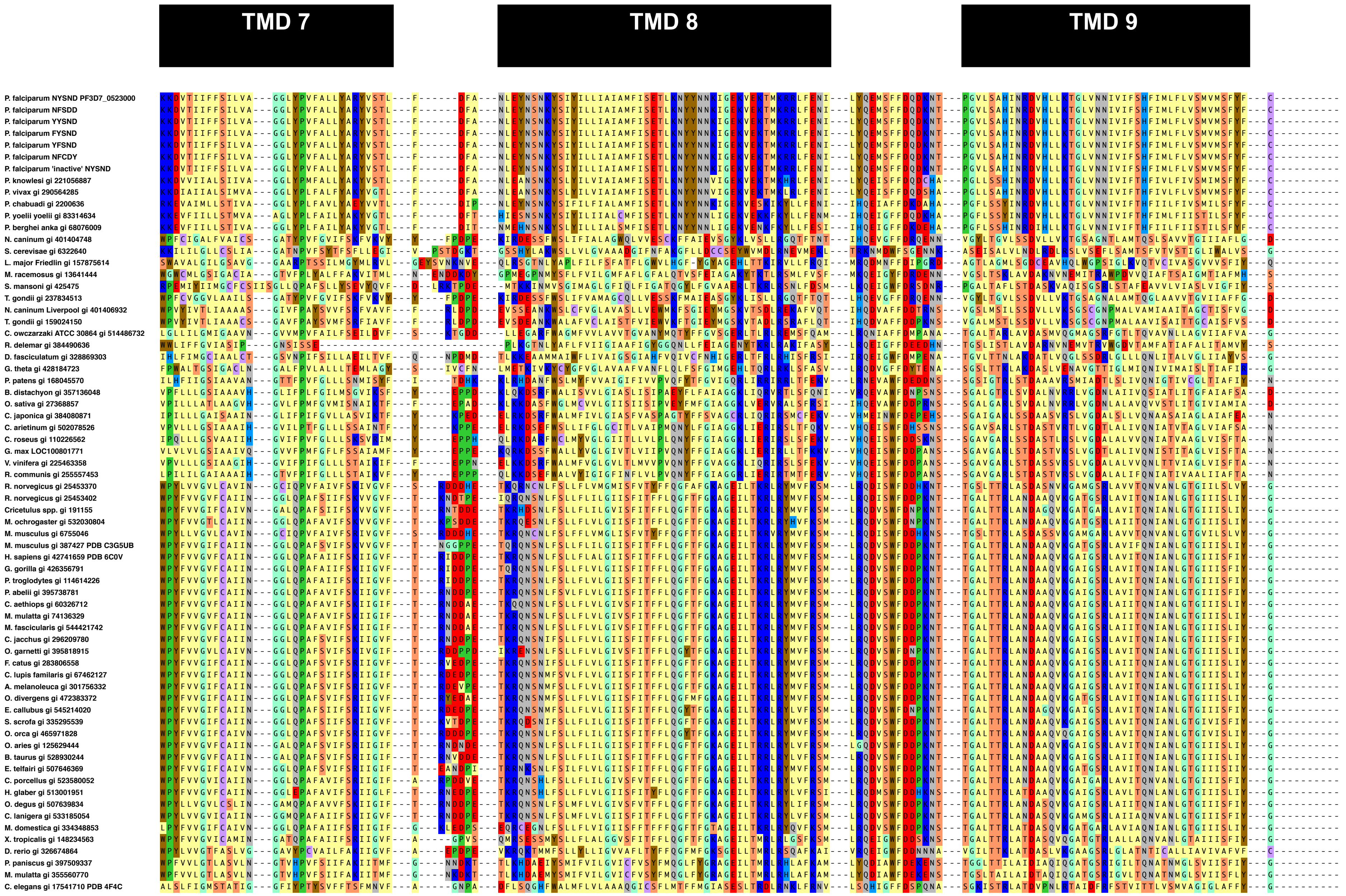

TMD 7
TMD 8
TMD 9
P. falciparum NYSND PF3D7_0523000
P. falciparum NFSDD
P. falciparum YYSND
P. falciparum FYSND
P. falciparum YFSND
P. falciparum NFCDY
P. falciparum 'inactive' NYSND
P. knowlesi gi 221056887
P. vivax gi 290564285
P. chabuadi gi 2200636
P. yoelii yoelii gi 83314634
P. berghei anka gi 68076009
N. caninum gi 401404748
S. cerevisae gi 6322640
L. major Friedlin gi 157875614
M. racemosus gi 13641444
S. mansoni gi 425475
T. gondii gi 237834513
N. caninum Liverpool gi 401406932
T. gondii gi 159024150
C. owczarzaki ATCC 30864 gi 514486732
R. delemar gi 384490636
D. fasciculatum gi 328869303
G. theta gi 428184723
P. patens gi 168045570
B. distachyon gi 357136048
O. sativa gi 27368857
C. japonica gi 384080871
C. arietinum gi 502078526
C. roseus gi 110226562
G. max LOC100801771
V. vinifera gi 225463358
R. communis gi 255557453
R. norvegicus gi 25453370
R. norvegicus gi 25453402
Cricetulus spp. gi 191155
M. ochrogaster gi 532030804
M. musculus gi 6755046
M. musculus gi 387427 PDB C3G5UB
H. sapiens gi 42741659 PDB 6C0V
G. gorilla gi 426356791
P. troglodytes gi 114614226
P. abelii gi 395738781
C. aethiops gi 60326712
M. mulatta gi 74136329
M. fascicularis gi 544421742
C. jacchus gi 296209780
O. garnetti gi 395818915
F. catus gi 283806558
C. lupis familaris gi 67462127
A. melanoleuca gi 301756332
O. divergens gi 472383372
E. callubus gi 545214020
S. scrofa gi 335295539
O. orca gi 465971828
O. aries gi 125629444
B. taurus gi 528930244
E. telfairi gi 507646369
C. porcellus gi 523580052
H. glaber gi 513001951
O. degus gi 507639834
C. lanigera gi 533185054
M. domestica gi 334348853
X. tropicalis gi 148234563
D. rerio gi 326674864
P. paniscus gi 397509337
M. mulatta gi 355560770
C. elegans gi 17541710 PDB 4F4C

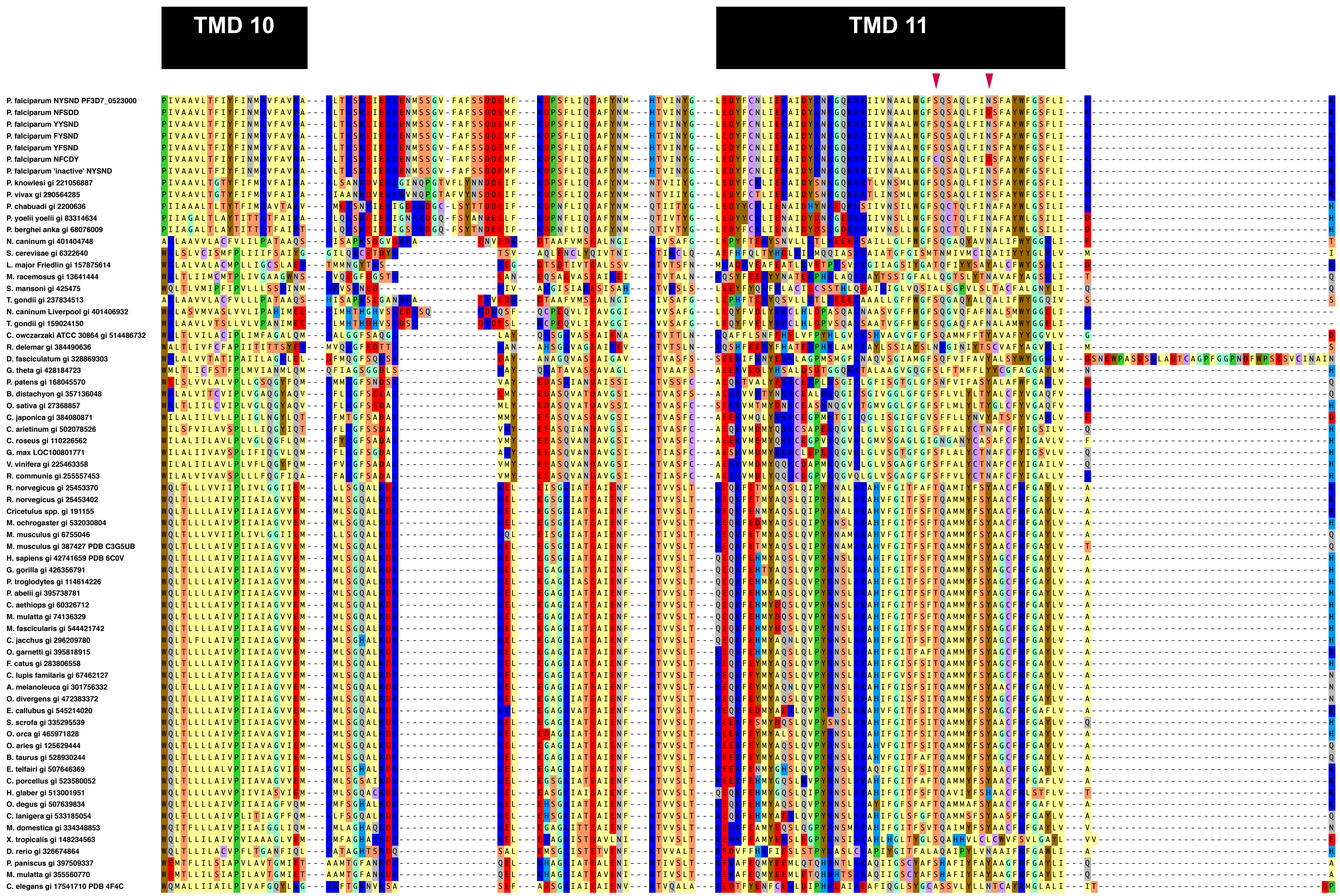
TMD 10
TMD 11
P. falciparum NYSND PF3D7_0523000
P. falciparum NFSDD
P. falciparum YYSND
P. falciparum FYSND
P. falciparum YFSND
P. falciparum NFCDY
P. falciparum 'inactive' NYSND
P. knowlesi gi 221056887
P. vivax gi 290564285
P. chabuadi gi 2200636
P. yoelii yoelii gi 83314634
P. berghei anka gi 68076009
N. caninum gi 401404748
S. cerevisae gi 6322640
L. major Friedlin gi 157875614
M. racemosus gi 13641444
S. mansoni gi 425475
T. gondii gi 237834513
N. caninum Liverpool gi 401406932
T. gondii gi 159024150
C. owczarzaki ATCC 30864 gi 514486732
R. delemar gi 384490636
D. fasciculatum gi 328869303
G. theta gi 428184723
P. patens gi 168045570
B. distachyon gi 357136048
O. sativa gi 27368857
C. japonica gi 384080871
C. arietinum gi 502078526
C. roseus gi 110226562
G. max LOC100801771
V. vinifera gi 225463358
R. communis gi 255557453
R. norvegicus gi 25453370
R. norvegicus gi 25453402
Cricetulus spp. gi 191155
M. ochrogaster gi 532030804
M. musculus gi 6755046
M. musculus gi 387427 PDB C3G5UB
H. sapiens gi 42741659 PDB 6C0V
G. gorilla gi 426356791
P. troglodytes gi 114614226
P. abelii gi 395738781
C. aethiops gi 60326712
M. mulatta gi 74136329
M. fascicularis gi 544421742
C. jacchus gi 296209780
O. garnetti gi 395818915
F. catus gi 283806558
C. lupis familaris gi 67462127
A. melanoleuca gi 301756332
O. divergens gi 472383372
E. callubus gi 545214020
S. scrofa gi 335295539
O. orca gi 465971828
O. aries gi 125629444
B. taurus gi 528930244
E. telfairi gi 507646369
C. porcellus gi 523580052
H. glaber gi 513001951
O. degus gi 507639834
C. lanigera gi 533185054
M. domestica gi 334348853
X. tropicalis gi 148234563
D. rerio gi 326674864
P. paniscus gi 397509337
M. mulatta gi 355560770
C. elegans gi 17541710 PDB 4F4C

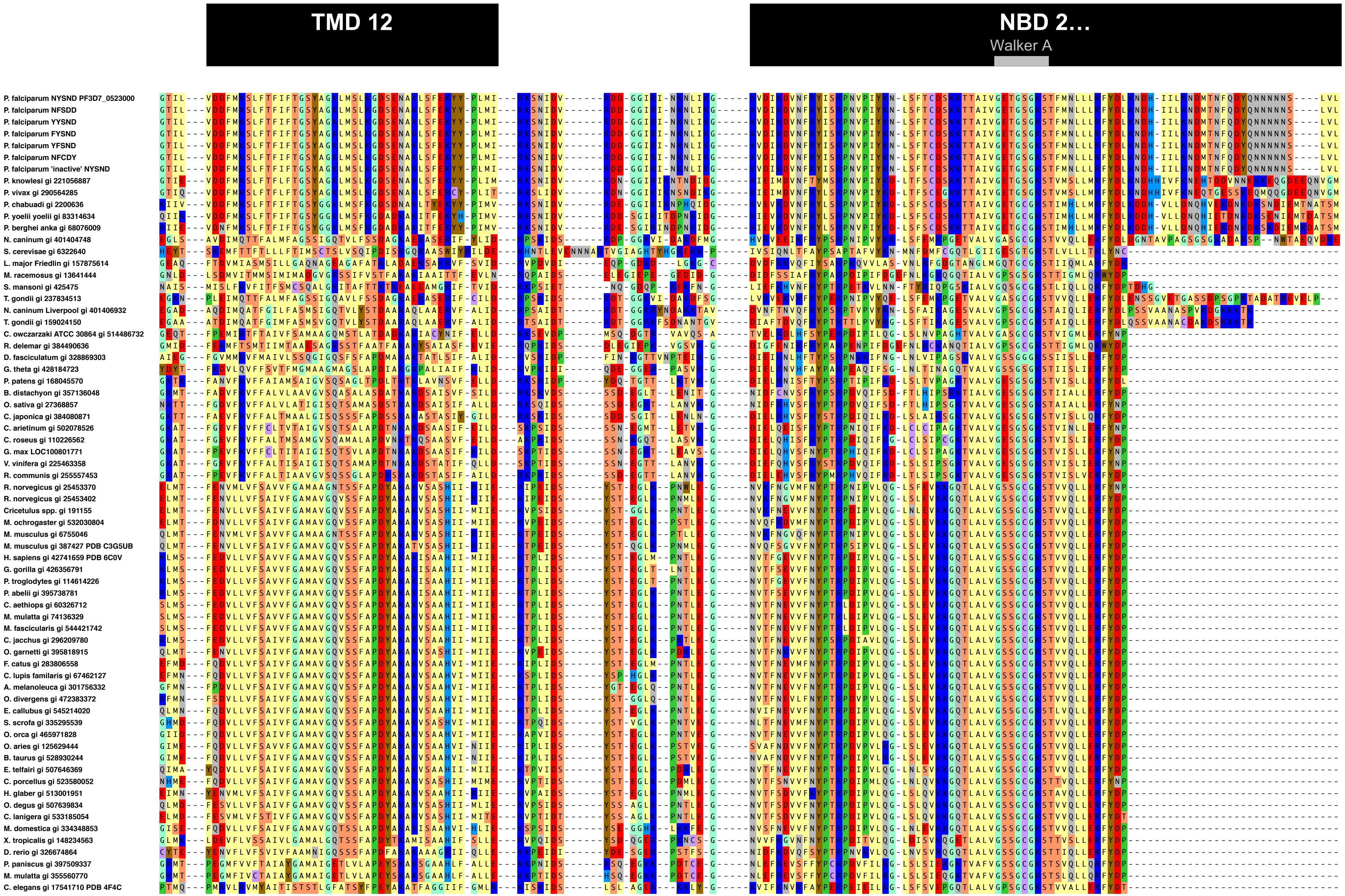
TMD 12
NBD 2...
Walker A
P. falciparum NYSND PF3D7_0523000
P. falciparum NFSDD
P. falciparum YYSND
P. falciparum FYSND
P. falciparum YFSND
P. falciparum NFCDY
P. falciparum 'inactive' NYSND
P. knowlesi gi 221056887
P. vivax gi 290564285
P. chabuadi gi 2200636
P. yoelii yoelii gi 83314634
P. berghei anka gi 68076009
N. caninum gi 401404748
S. cerevisae gi 6322640
L. major Friedlin gi 157875614
M. racemosus gi 13641444
S. mansoni gi 425475
T. gondii gi 237834513
N. caninum Liverpool gi 401406932
T. gondii gi 159024150
C. owczarzaki ATCC 30864 gi 514486732
R. delemar gi 384490636
D. fasciculatum gi 328869303
G. theta gi 428184723
P. patens gi 168045570
B. distachyon gi 357136048
O. sativa gi 27368857
C. japonica gi 384080871
C. arietinum gi 502078526
C. roseus gi 110226562
G. max LOC100801771
V. vinifera gi 225463358
R. communis gi 255557453
R. norvegicus gi 25453370
R. norvegicus gi 25453402
Cricetulus spp. gi 191155
M. ochrogaster gi 532030804
M. musculus gi 6755046
M. musculus gi 387427 PDB C3G5UB
H. sapiens gi 42741659 PDB 6C0V
G. gorilla gi 426356791
P. troglodytes gi 114614226
P. abelii gi 395738781
C. aethiops gi 60326712
M. mulatta gi 74136329
M. fascicularis gi 544421742
C. jacchus gi 296209780
O. garnetti gi 395818915
F. catus gi 283806558
C. lupis familaris gi 67462127
A. melanoleuca gi 301756332
O. divergens gi 472383372
E. callubus gi 545214020
S. scrofa gi 335295539
O. orca gi 465971828
O. aries gi 125629444
B. taurus gi 528930244
E. telfairi gi 507646369
C. porcellus gi 523580052
H. glaber gi 513001951
O. degus gi 507639834
C. lanigera gi 533185054
M. domestica gi 334348853
X. tropicalis gi 148234563
D. rerio gi 326674864
P. paniscus gi 397509337
M. mulatta gi 355560770
C. elegans gi 17541710 PDB 4F4C

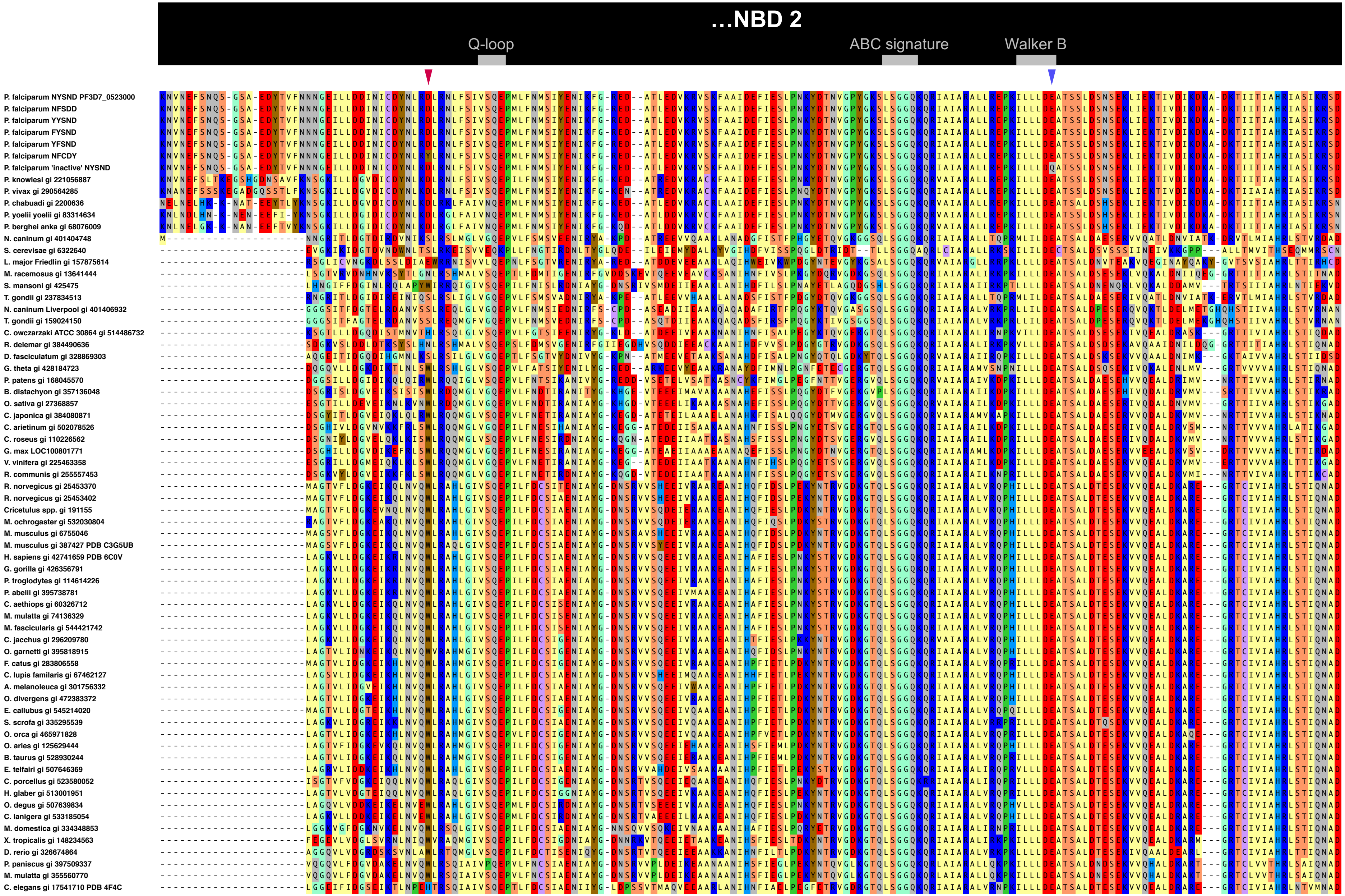

...NBD 2
Q-loop
ABC signature
Walker B
P. falciparum NYSND PF3D7_0523000
P. falciparum NFSDD
P. falciparum YYSND
P. falciparum FYSND
P. falciparum YFSND
P. falciparum NFCDY
P. falciparum 'inactive' NYSND
P. knowlesi gi 221056887
P. vivax gi 290564285
P. chabaudi gi 2200636
P. yoelii yoelii gi 83314634
P. berghei anka gi 68076009
N. caninum gi 401404748
S. cerevisae gi 6322640
L. major Friedlin gi 157875614
M. racemosus gi 13641444
S. mansoni gi 425475
T. gondii gi 237834513
N. caninum Liverpool gi 401406932
T. gondii gi 159024150
C. owczarzaki ATCC 30864 gi 514486732
R. delemar gi 384490636
D. fasciculatum gi 328869303
G. theta gi 428184723
P. patens gi 168045570
B. distachyon gi 357136048
O. sativa gi 27368857
C. japonica gi 384080871
C. arietinum gi 502078526
C. roseus gi 110226562
G. max LOC100801771
V. vinifera gi 225463358
R. communis gi 255557453
R. norvegicus gi 25453370
R. norvegicus gi 25453402
Cricetulus spp. gi 191155
M. ochrogaster gi 532030804
M. musculus gi 6755046
M. musculus gi 387427 PDB C3G5UB
H. sapiens gi 42741659 PDB 6C0V
G. gorilla gi 426356791
P. troglodytes gi 114614226
P. abelii gi 395738781
C. aethiops gi 60326712
M. mulatta gi 74136329
M. fascicularis gi 544421742
C. jacchus gi 296209780
O. garnetti gi 395818915
F. catus gi 283806558
C. lupis familaris gi 67462127
A. melanoleuca gi 301756332
O. divergens gi 472383372
E. callubus gi 545214020
S. scrofa gi 335295539
O. orca gi 465971828
O. aries gi 125629444
B. taurus gi 528930244
E. telfairi gi 507646369
C. porcellus gi 523580052
H. glaber gi 513001951
O. degus gi 507639834
C. lanigera gi 533185054
M. domestica gi 334348853
X. tropicalis gi 148234563
D. rerio gi 326674864
P. paniscus gi 397509337
M. mulatta gi 355560770
C. elegans gi 17541710 PDB 4F4C

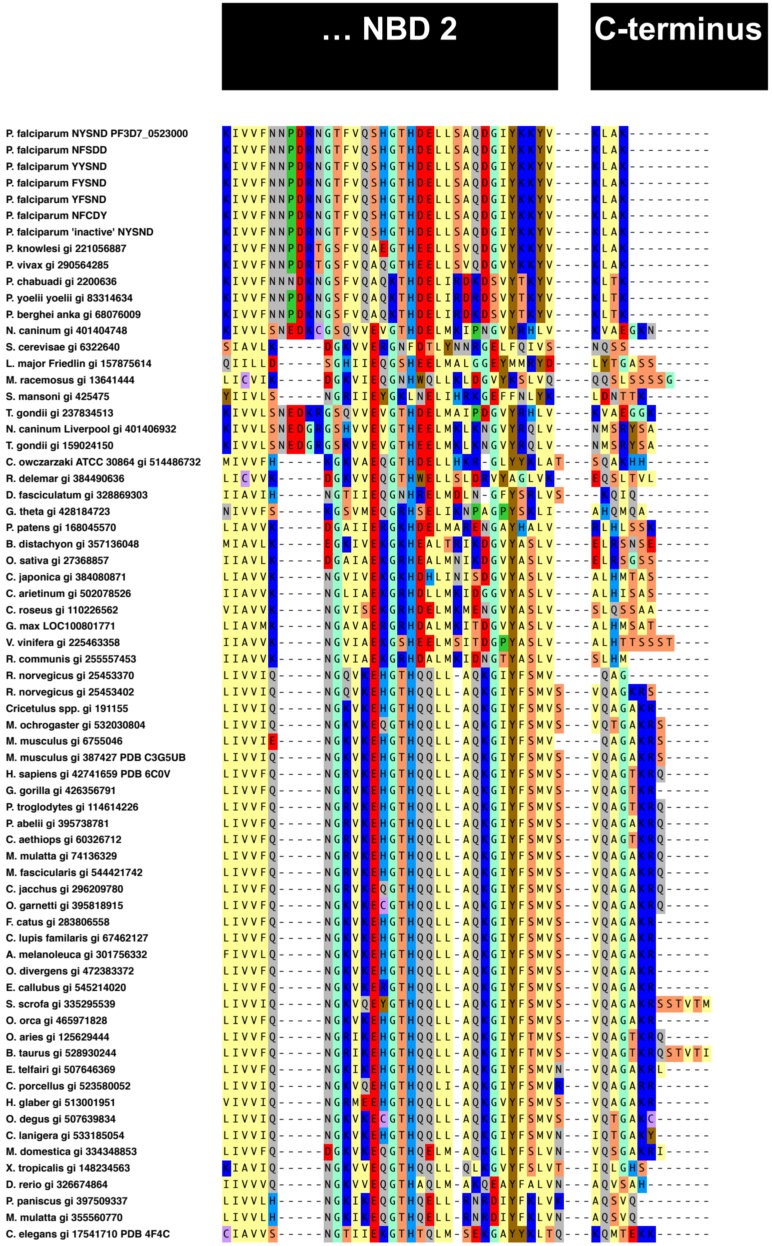

Alignment of MDR1 proteins used to generate the PfMDR1 homology model.
A selection of MDR1 proteins from different organisms, including the *H. sapiens, M. musculus*, and *C. elegans* homologues that have had their structures determined [1, 2, 3, 4], were aligned using ClustalW and the resulting alignment was manually edited in MacVector 12.7.5. The gene identifier (NCBI accession number — PF3D7, gi, or LOC) of each protein is given. The sequence of PfMDR1^{NYSND} is at the top of the alignment followed by the sequences of the other PfMDR1 isoforms included for study. The regions predicted to span the transmembrane domains (TMDs), the nucleotide-binding domains (NBDs), and the N- and C-termini are marked with black bars. Residues are shaded as follows: positively charged (arginine and lysine), dark blue; negatively charged (aspartic acid and glutamic acid), red; serine and threonine, orange; aromatic (tyrosine and tryptophan), brown; asparagine and glutamine, gray; cysteine, purple; proline, green; histidine, blue; glycine, turquoise; non-polar (methionine, alanine, isoleucine, leucine, valine, and phenylalanine), yellow. Pink triangles denote the globally prevalent polymorphisms found at positions 86, 184, 1034, 1042, and 1246 in PfMDR1. Blue triangles denote the mutations made within the Walker B motif of each NBD to generate a catalytically-inactive version of PfMDR1^{NYSND}. Consensus sequences within the NBDs are annotated with gray boxes to indicate the positions of the Walker A and B motifs, the ABC signature sequences, and the Q-loops.

1. Jin MS, Oldham ML, Zhang Q, Chen J. Crystal structure of the multidrug transporter P-glycoprotein from *Caenorhabditis elegans*. Nature. 2012;490: 566-569.

2. Kim Y, Chen J. Molecular structure of human P-glycoprotein in the ATP-bound, outward-facing conformation. Science (New York, NY). 2018;359: 915-919.

3. Li J, Jaimes KF, Aller SG. Refined structures of mouse P-glycoprotein. Protein science: a publication of the Protein Society. 2014;23: 34-46.

4. Ward AB, Szewczyk P, Grimard V, Lee C, Martinez L, Doshi R, et al. Structures of P-glycoprotein reveal its conformational flexibility and an epitope on the nucleotide-binding domain. Proc Natl Acad Sci USA. 2013;110: 13386-13391.
